# Energy demand in an active videogame session and the potential to promote hypotension after exercise in hypertensive women

**DOI:** 10.1371/journal.pone.0207505

**Published:** 2018-12-13

**Authors:** Tais Feitosa da Silva, Maria do Socorro Cirilo-Souza, Marizângela Ferreira de Souza, Geraldo Veloso Neto, Marcos Antônio Pereira dos Santos, Alexandre Sérgio Silva

**Affiliations:** 1 Physical Education Department, Federal University of Paraíba, Paiaíba, Brazil; 2 Biophysics and Physiology Department, Federal University of Piauí, Piauí, Brazil; Sao Paulo State University - UNESP, BRAZIL

## Abstract

The aim of this study was to determine the energy demand of one session of active video game (AVG) and its potential to reduce blood pressure (BP). Fourteen hypertensive (56.4±7.5 years) individuals performed sessions of AVG, a traditional sedentary video game sessions (SVG) and walking (WAL), as negative and positive controls, in a randomly determined order. Oxygen consumption and energy expenditure (EE) were measured during sessions. BP and cardiac autonomic modulation (CAM) were measured at rest and every 15 minutes of a 60-minute period of recovery from activities. A rating of enjoyment scale was also applied. AVG and WAL resulted in higher oxygen consumption (10.0±0.5 ml/kg/min and 16.6±3.1 ml/kg/min, respectively) and EE (3.5±0.2 kcal/min and 4.2±0.5 kcal/min) compared to 4.1±0.8 ml/kg/min and 1.4±0.1 kcal/min in SVG. A reduction in systolic and diastolic BP was evident following AVG sessions (-11.6±2.5 mmHg and -8.7±2.5 mmHg) and WAL (-10.8±2.8 mmHg and -8.6±2.3 mmHg) compared to pre-experiment value, and the same did not occur in SVG. All sessions promoted a feeling of enjoyment, with no difference between them. The parasympathetic activity was significantly lower at 30 and 45 minutes in post-WAL recovery (34.6±15.0 ms^2^ and 34.4±16.0 ms^2^) in the frequency domain (HF) in relation to both AVG (195.5±67.0 ms^2^ and 164.5±55.0 ms^2^) and the SVG (158.9±45.0 and 281.3±98.0 ms^2^). It is concluded that an AVG session promotes increased metabolic activity and is able to promote acute reduction of BP in hypertensive individuals similar to traditional walking exercise.

## Introduction

Post-exercise hypotension (PEH) is a clinically relevant phenomenon, characterized by reduced blood pressure that occurs in the first minutes after an exercise session. It has been demonstrated since 1981 [1] and has been confirmed with a high level of evidence in recent decades [2]. This high level of evidence was obtained based on studies with aerobic exercise, especially in terms of walking and running. Afterwards, PEH was tested and confirmed in several other modalities, mainly in resistance exercises [3] and high-intensity interval training [4], although with values of lesser magnitude than those promoted by aerobic exercise. Even modalities with low metabolic demands have proved to be capable of promoting PEH, such as yoga [5] and tai chi chuam [6].

Although videogames are a form of modern entertainment and due to the increase in screen time and, consequently in sedentarism, the emergence of consoles that allow the practice of physically active videogames has a greater possibility of drawing more interest. In fact, cardiometabolic demand has been considered comparable to physical exercise, particularly in children and adolescents, who are the most investigated people in this form of entertainment [7–9].

This increased cardiometabolic demand in children and adolescents lead to our team hypothesizing that it could be reproduced in middle-aged hypertensive patients and would result in post-game blood pressure reduction, similar to the PEH phenomenon. Therefore, his hypothesis was tested in this study with a group of hypertensive middle-aged women. The aim was to compare blood pressure responses following an active video game (AVG) session to sedentary video game sessions (SVG), with a walking session (WAL) as positive control. An enjoyment scale was further introduced into the study to determine possible influences of game entertainment on the pressor response. Autonomic activity measures were also adopted to perform an initial check of possible mechanisms involved in any reduction in pressure promoted by AVG.

## Materials and methods

### Participants

**Fig 1:**
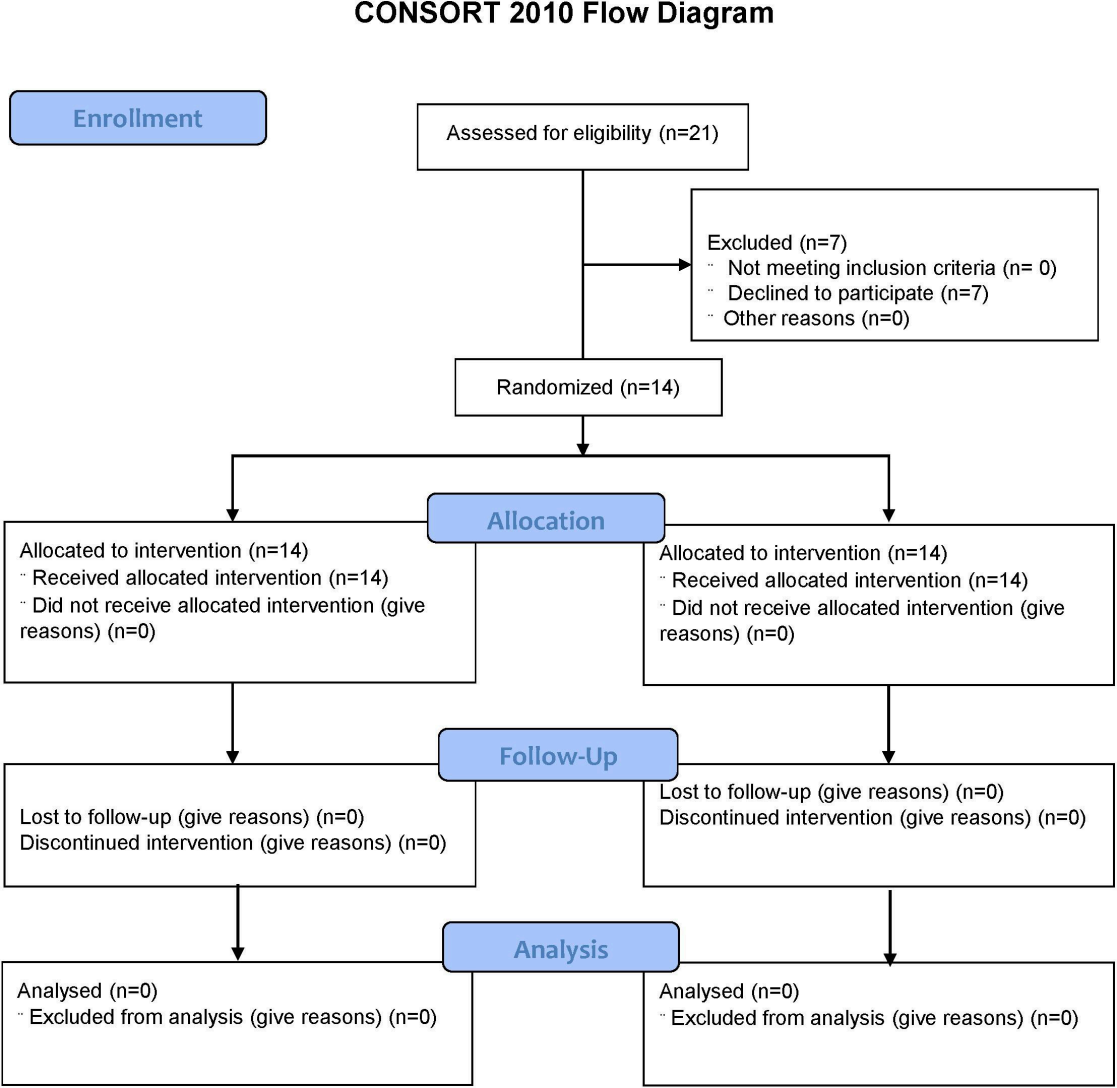
Consort flowchart for inclusion, allocation, sample tracking, and data analysis. n = sample size. Source: Silva, 2015.

In a previous pilot study, seven women with resting systolic Blood Pressure (BP) of 128.9±9.4 mmHg obtained a reduction to 116.9 ± 7.0 mmHg, 60 minutes post-exercise with AVG. Using the difference between two dependent means on Gpower software (version 3.1.9), these data resulted in an effect size of 1.41. Then, a minimum sample size of 8 subjects was estimated, considering an α error of 0.05 (one sided) and statistical power of 0.95. Twenty-one participants were included in the study, however seven participants withdrew. So, 14 hypertensive women composed the final sample (Fig 1), aged between 47 and 68 years (56.4±7.5). This sample size achieved a sample power of 0.99 to an α error of 0.01.

None of the women had symptoms or labyrinthitis diagnosis. They had blood pressure values of at least 130/90 mmHg and not higher than 160/110 mmHg in measurements made in days prior to the study and on the day of the data collection. All women were post-menopausal, overweight or 1^st^ degree obesity (BMI between 25 and 34.9 Kg/m^2^), and had not practiced systematic exercise for at least three months. Volunteers who initiated any anti-hypertensive treatment during the study period, who presented difficulties in carrying out AVG tests or who took more than four weeks to complete all the experimental procedures were excluded from the study. No participants were excluded during the statistical analysis procedure (Fig 1). Participants were studied between February and April 2015.

This research was approved by the Research Ethics Committee of the Health Science Center—UFPB under the protocol n° 0621/14. The volunteers provided a written consent form after having been informed about all the procedures, and in agreement with Resolution 466/12 of the National Health Council of Brazil (Clinical Trials n° NCT02924012).

### Study design

**Fig 2:**
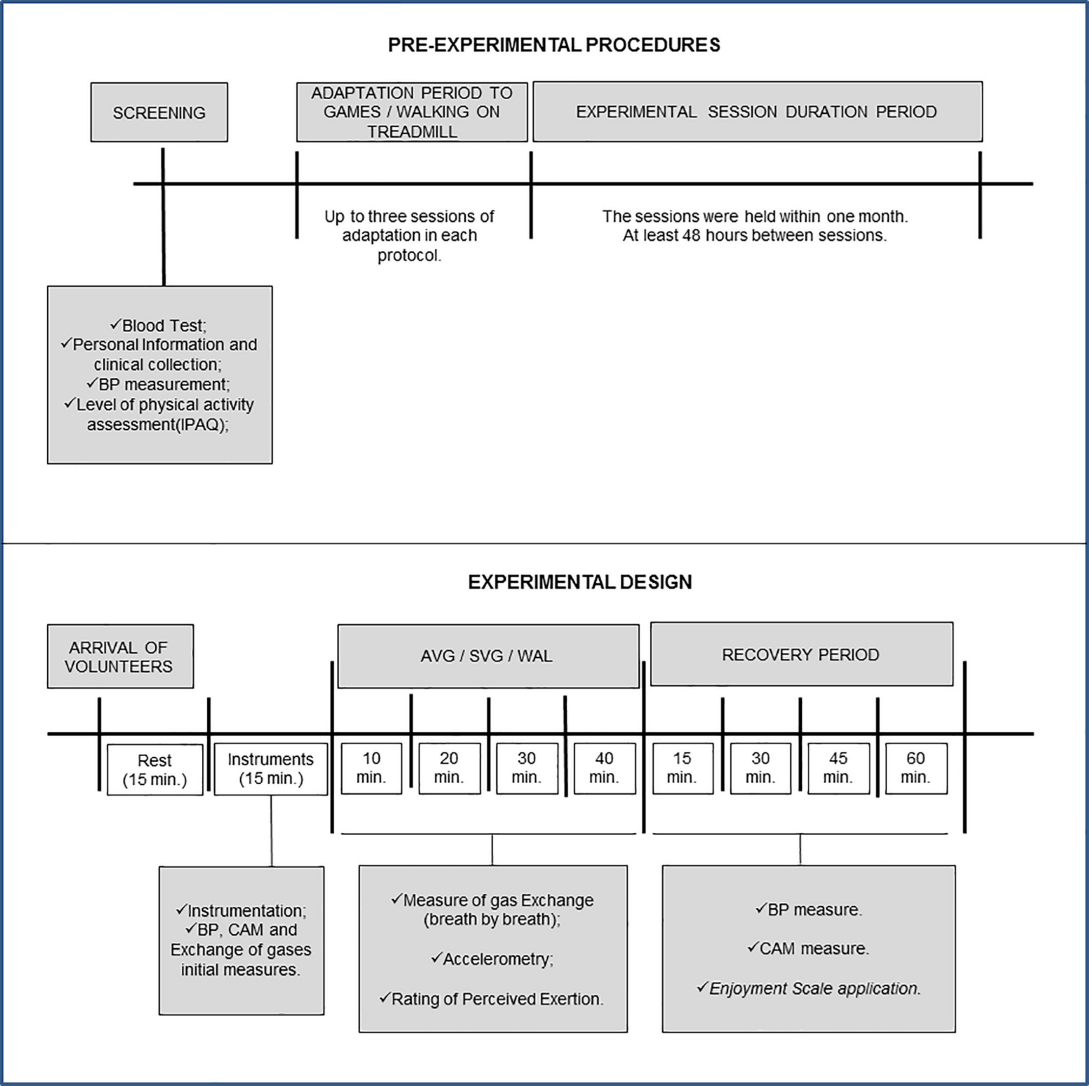
**Pre-experimental procedures and experimental design of the sessions.** AVG = active video game; SVG = sedentary video game; WAL = walking; BP = blood pressure; CAM = cardiac autonomic modulation.

The volunteers performed an AVG session, an internal control session consisting of SVG game and an external positive control session consisting of a walking exercise (WAL) with a minimum interval of 48 hours between sessions and randomly determined order. Prior activities in all sessions, they were instrumented with a portable gas analyzer for measuring energy expenditure (EE), an accelerometer to quantify performed movements and a heart rate monitor to evaluate the behavior of the heart rate (HR). BP and cardiac autonomic modulation (CAM) measurements were made at rest and every 15 minutes of a 60-minutes recovery period from activities. A rating scale of enjoyment in activities (enjoyment scale) was applied in these same times (Fig 2).

The researcher responsible for researching and conducting data collection performed a randomization of the sessions at *www*.*randomizer*.*org*. Data collection was carried out in the Laboratory of Applied Physics for Performance and Health (LETFADS) located in the Department of Physical Education—Federal University of Paraíba, Brazil. Data collection was carried out in the year 2015 between the months of March and June.

### Pre-experimental procedures

Prior to the experimental sessions, BP, physical activity assessments and blood collection were carried out to characterize the participants. Personal information on the drugs used was collected and they were given a list containing which types of foods and caffeinated products that could not be ingested 24 h before each experimental session.

### Familiarization activities

The participants performed three sessions of familiarization, learning the handling of active and sedentary games and walking on a treadmill. The sessions lasted from 10 to 40 minutes, with an interval of 48 hours between sessions and at least 72 hours between the last familiarization session and the first test session. The intensity adopted in these sessions was free, but participants were encouraged to achieve moderate intensity on the treadmill exercise (60% to 85% of maximum HR). They were also taught to respond to the subjective perception questionnaires on the effort and enjoyment scale.

### Active video game

The console used was an Xbox 360 with Kinect sensor (Foxconn, New Taipei, China) and the game used was Dance Central (Harmonix Music Systems—Microsoft Games Studios). The difficulty level set was beginner, since the participants had no previous experience with AVG. In the game, body movements were similar to dance steps with the use of different pop songs, from the 1970´s, 1980´s, 1990´s and 2000´s. The session lasted 40 minutes.

### Sedentary video game

The Dance Dance Revolution game was used (SSD Company Ltd., Shiga, Japan) for Playstation 2 (San Mateo, CA, USA), where the practitioner simulated synchronized dances of similar songs to those used in gyms, but using the traditionally-known joystick, so that players remained seated throughout the session. The duration of the game was 40 minutes.

### Walking exercise

The participants performed a walking session on a treadmill (Moviment, Brudden Ltda, São Paulo, Brazil) for 40 minutes at a previously prescribed intensity, between 60% and 80% of maximum HR.

### Accelerometry

An Actigraph GT3X (Pensacola, USA) accelerator was used to assess body acceleration, calibrated according to the manufacturer's specifications. The time interval adopted was 60 seconds and the output data were expressed as mean counts per minute. The unit was set at waist height on the right hand by an elastic strap and adjustable buckle. At the end of the recording, data were transferred to a computer and analyzed using the SAS 9.2 software (SAS Institute Inc., Cary, NC 25513). The reference values adopted were 0–99 counts / min, considered as sedentary activity; 100–1951 counts / min, considered as light activity; 1952–5724 counts / min, considered as moderate activity, and from 5725 counts / min considered as vigorous activity, according to Freedson et al. [10].

### Energy demand

Energy demand was measured by gas analysis through a portable analyzer of K4b2 pulmonary gases (Cosmed Copyritgh, Rome, Italy), validated in the study by McLaughlin et al. [11]. This equipment enables verification of respiratory parameters in each breath, measuring the oxygen consumption (VO_2_) and carbon dioxide production during exercise. The device was calibrated according to the manufacturer's recommendations. The variables measured by this instrument were the EE and VO_2_. The measurements were performed at rest (for five minutes) and during experimental sessions.

### Heart rate and perceived exertion

HR was measured using a RS800CX Polar heart rate monitor (Polar ElectroOy, Kempele, Finland). The measurements were made after 10 minutes at rest and every 5 minutes during exercises. The Subjective Perception Scale of Borg effort [12] with a scale ranging between 6–20 points was presented to the participants in the same times when HR was measured; they were asked to say whether they had any feelings of stress.

### Enjoyment scale

This scale has been validated for use in the adult population according to Graves et al. [13]. It consisted of 18 items that rated the level of pleasure from levels ranging from "I like it" to "I hate it", where the higher the sum of these points, the higher the pleasure in the performed activity. This scale was presented to participants at the beginning of each study session, and was answered at the end of each study session.

### Blood pressure

Participants were asked to rest for 15 minutes in a seated position before each session. Resting BP was then measured after this period. Further measures were taken immediately at the end of the experimental session and every 15 minutes during a 60-minute recovery period, with the volunteers seated. These measurements were performed by the oscillometric method with the use of a BP ambulatory monitoring device (DYNAMAPA), following the V Brazilian Guidelines for Ambulatory BP (MAP) [14]. Each blood pressure measurement was done in triplicate and the two closest values were considered.

### Cardiac autonomic modulation

Immediately after each BP measurement, the CAM was measured through HR variability using a Polar RS800CX monitor (PolarElectroOy, Kempele, Finland). The volunteers were seated and the record was made for a minimum of 10 minutes. Data were transferred to a computer provided with the software of the same manufacturer and then transferred to the Kubios HRV software, version 2.0 (University of Kuopio, Finland). Data were analyzed in the time domain, considering the average (MRR) and the standard deviation of the RR (SDNN) individual intervals, the root mean square of successive differences between adjacent RR (RMSSD) and the percentage of successive differences between RR intervals higher than 50 ms (pNN50). In the frequency domain, 0.04 to 0:15 Hz was considered as the low-frequency band (LF) and 0.15 to 0.4 Hz as high-frequency band (HF). The low-ratio frequency/high-ratio frequency were also adopted as sympathetic-vagal balance (LF/HF).

### Statistical analysis

Data are presented as mean and standard error of the mean. The Shapiro-Wilk test was used to verify the normality, and Levene test to check homogeneity. One-way ANOVA was used to compare the resting values. Two-way ANOVA for repeated measures was adopted to compare experimental protocols considering the interaction time and procedures. In the absence of sphericity, the Greenhouse-Geisser test was considered. Post hoc Bonferroni test was adopted. Data were analyzed using IMB SPSS. Version 24, at a significance of p <0.05.

## Results

In addition to grade-I hypertensive, the volunteers were hyperglycemic, hyperlipidemic, and were at the boundary between grade-I overweight and obesity, as can be seen in the mean values for these variables in Table 1. They were medically treated with diuretics, angiotensin-converting enzyme inhibitors and AT1 receptor blockers. Most of the subjects (57.2%) were pharmacologically treated for monotherapeutic form and 42.8% used both drugs simultaneously.

**Table 1:** Hemodynamic, metabolic and autonomic characteristics of the study participants at the beginning of each experimental session (rest).

Variables	Values		
**Age (years)**	56.4±7.5		
**Body Mass Index (kg/m^2^)**	30.1±1.4		
**Glycemia (mg/dL)**	143.9±29.0		
**Triglycerides (mg/dL)**	219.0±55.8		
**Total Cholesterol total (mg/dL)**	244.4±28.8		
**LDL Cholesterol (mg/dL)**	171.6±33.1		
**HDL Cholesterol (mg/dL)**	46.3±3.4		
**VLDL Cholesterol (mg/dL)**	43.8±11.1		
	**SVG**	**AVG**	**WAL**
**SBP (mmHg)**	132.7±1.9	136.2±5.4	135.1±1.6
**DBP (mmHg)**	87.5±1.8	88.4±2.6	92.9±0.8
**EE (kcal/min)**	1.2±0.0	1.8±0.0	1.3±0.2
**VO**_**2**_ **(ml/kg/min)**	3.6±0.2	3.8±0.1	3.7±0.1
**HR (spm)**	84.2±2.4	82.4±2.6	90.3±4.7
**MRR (ms^2^)**	655.4±40.4	655.2±75.7	667.1±35.7
**SDNN (ms^2^)**	42.6±9.1	22.4±5.2	44.8±14.0
**RMSSD (ms)**	19.4±7.4	14.5±2.6	16.4±3.8
**pNN50 (%)**	3.4±1.9	1.9±0.7	3.8±1.3
**LF (ms^2^)**	380.3±249.0	116.8±47.0	162.3±65.0
**HF (ms^2^)**	90.6±34.0	64.5±21.0	55.6±25.0
**LF/HF**	1.7±0.4	1.7±0.2^@^	2.9±0.5

Data are mean and standard error of the mean. SBP—systolic blood pressure; DBP—diastolic blood pressure; EE—energy expenditure at rest; HR—heart rate; MRR—average of RR segments; SDNN—the standard deviation of RR segments; RMSSD—root mean square of successive differences between adjacent R-R; pNN50—percentage of differences between successive R-R intervals greater than 50 ms; LF—low frequency spectrum; HF—High-Frequency Spectrum; LF / HF—sympathetic-vagal balance. (N = 14) one-way ANOVA followed by Tukey's post-test, p <0.05.

@ = Difference with WAL. Source: Silva, 2015.

The volunteers presented similar values for all variables that would be monitored in the study at the beginning of each experimental session, except for the sympathetic-vagal balance (LF/HF), which was significantly higher in WAL procedure in relation to AVG (Table 1).

Physical activity demanded by experimental protocols is presented in Table 2. It may be observed that AVG session resulted in a slightly larger uptime period, more moderate and vigorous than SVG, which in turn was marked by wide prevalence of sedentary behavior. Compared to WAL, AVG had longer moderate activity and shorter vigorous activity.

**Table 2:** Physical activity measures over 40 minutes of active video game practice, sedentary video game and walking exercise.

	SVG (n = 14)	AVG(n = 14)	WAL(n = 14)
**Sedentary state (min)**	39.4±0.2^#^ (98.5%)	2.0±0.5* (5.3%)	1.8±0.7 (5%)
**Light activity (min)**	0.6±0.2 (1,5%)	34.3±1.0^#^*^@^ (85.7%)	17.3±2.8 (43.3%)
**Moderate / vigorous activity (min)**	0 (0%)	3.6±1.5*^@^ (9.0%)	20.7±2.8^#^ (51.7%)

Data are mean and standard error of the mean. (N = 14) one-way ANOVA followed by Tukey's post-test. Sedentary state = 0 to 99 counts / min; light activity = 100–1951 counts/min; Moderate activity/strong = >1951 counts / min.

# = Intra-group difference

* = Difference in relation to the SVG

@ = Difference with WAL. p <0.05. Source: Silva, 2015.

**Fig 3:**
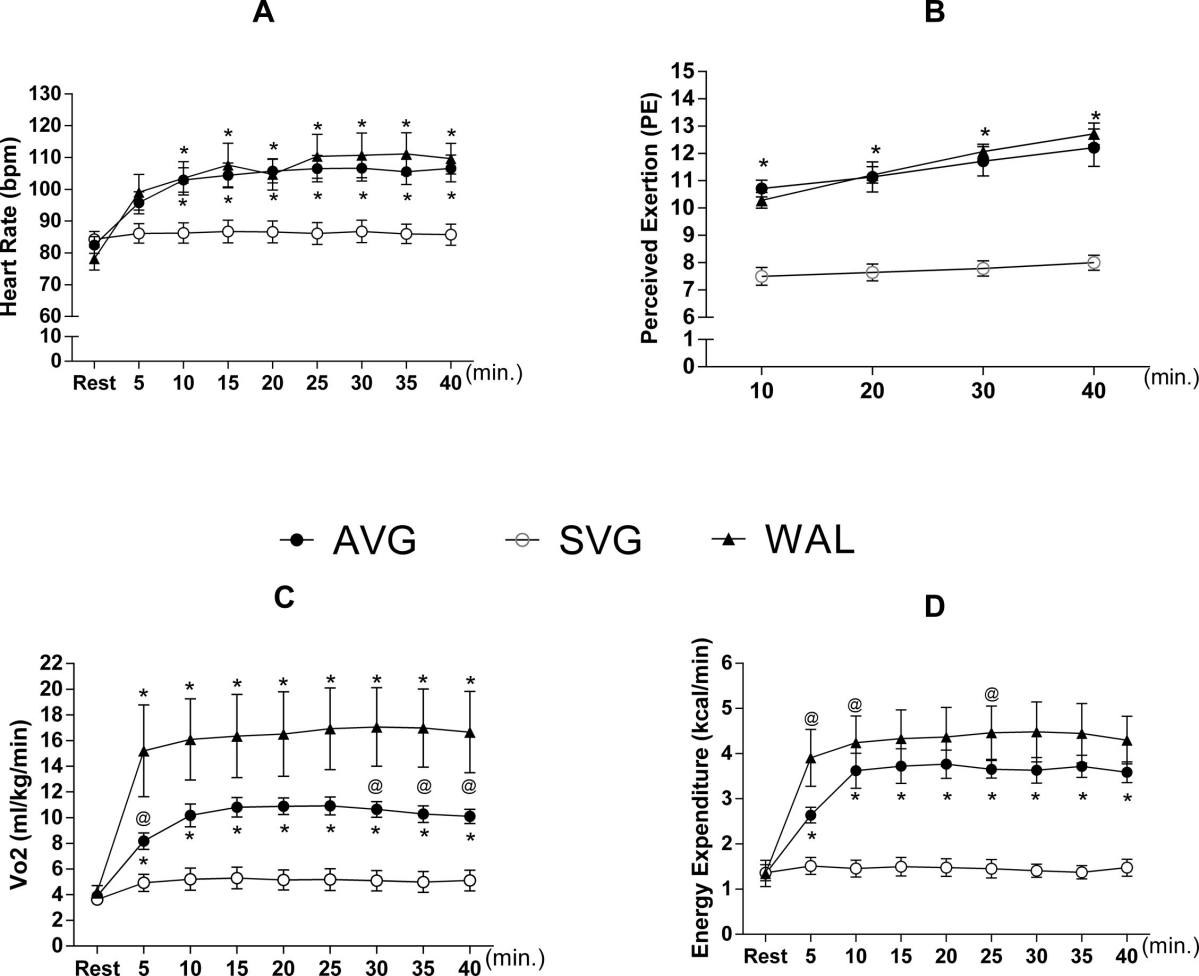
(A) Heart rate values (B) perceived exertion, (C) oxygen consumption values (VO_2_) and (D) energy expenditure (EE) measured breath by breath. Data are mean and standard error of the mean. (N = 14). Repeated measures two-way ANOVA with Bonferroni Post hoc. * = Difference in relation to the SVG; @ = Difference between AVG and WAL. Source: Silva, 2015.

The intensity is presented according to the HR recording data, the perceived exertion and the energy demand of the experimental sessions, as found in Fig 3. While HR was maintained with minimal changes during the SVG session in relation to rest, there was a significant increase in the AVG and WAL sessions (Fig 3A), without difference in the WAL versus AVG interaction. However, the average HR reached by volunteers in the WAL session was only 32.4±4.3% of maximum HR, far from the minimum of 60% expected to characterize walking as a moderate-intensity exercise. Even with this intensity, the volunteers reported having perceived exertion within what was regarded as a moderate-intensity exercise (between 11 and 14 points), with similar values between AVG and WAL, and significantly higher regarding SVG (Fig 3B). For this cardiovascular behavior and subjective perception, the ergospirometric data revealed a respiratory quotient of 0.86 and 0.97 for AVG and 0.95 and 0.97 for WAL during the accomplishment of the activities, indicating metabolic activity below the anaerobic threshold in these two experimental sessions.

AVG and WAL resulted in a significant increase in oxygen consumption during the sessions with effect size of 3.40 and 2.82 respectivelly, while SVG resulted in an increase that was discreet and not statistically different from the rest (effect size = 0.55). Oxygen consumption was greater in WAL and AVG compared to SVG at 5 minutes and remained statistically similar for the remaining activity (Fig 3C). AVG and WAL showed similar oxygen consumption at 10, 20 and 25 minutes, while WAL resulted in higher oxygen consumption than AVG at 5, 30, 35 and 40 min. The EE during the sessions had the same behavior of oxygen consumption, with a significant increase compared to rest on AVG (effect size = 3.06) and WAL (effect size = 3.74), only a slight increase and no statistical significance in SVG (effect size = 0.60) and increased WAL EE regarding AVG in 5, 10 and 25 minutes of the session (Fig 3D).

**Fig 4:**
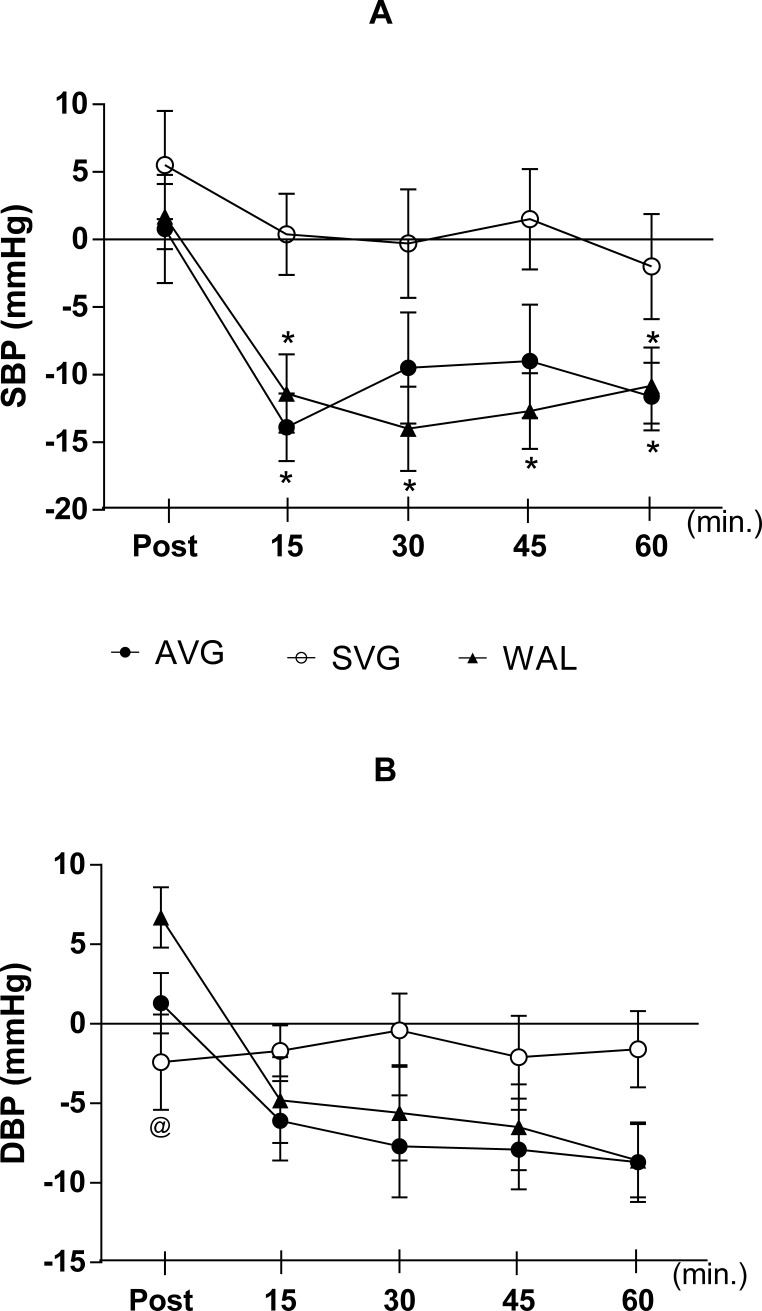
(A) Delta values of Systolic and (B) Diastolic blood pressure (60-minute recovery). Data are mean and standard error of the mean. (N = 14). Repeated measures two-way ANOVA followed by Bonferroni post-test. P <0.05. * = Difference in relation to the SVG; @ = Difference between AVG and WAL. Source: Silva, 2015.

Pressure responses to the experimental procedures are shown in Fig 4. While systolic BP remained unchanged during the 60 minutes following SVG session in relation to pre-experiment value, a reduction in pressure in AVG sessions (between 9 and 14 mmHg, with effect size reaching 0.63 compared to SVG) and WAL (between 11 and 14 mmHg, with effect size reaching 0.73 compared to SVG) was evident 15 to 60 minutes after exercise. The reduction values in systolic BP in WAL were significantly higher compared to SVG in all measurements from 15 to 60 minutes after experimental sessions. For AVG, the blood pressure reduction was significantly higher than in SVG at 15 and 60 minutes after procedures. There were no differences in any of the five measures after the game or walking between AVG and WAL.

AVG and WAL resulted in a clear reduction in diastolic BP 15 to 60 minutes after sessions, and diastolic BP after SVG remained virtually unchanged throughout the period after the game in relation to the present value (Fig 4). Compared to SVG, AVG and WAL effect sizes were 0.81 and 0.83 respectivelly. However, there were no statistical differences among the three procedures, except for the measurement made immediately after the SVG compared to WAL.

**Fig 5:**
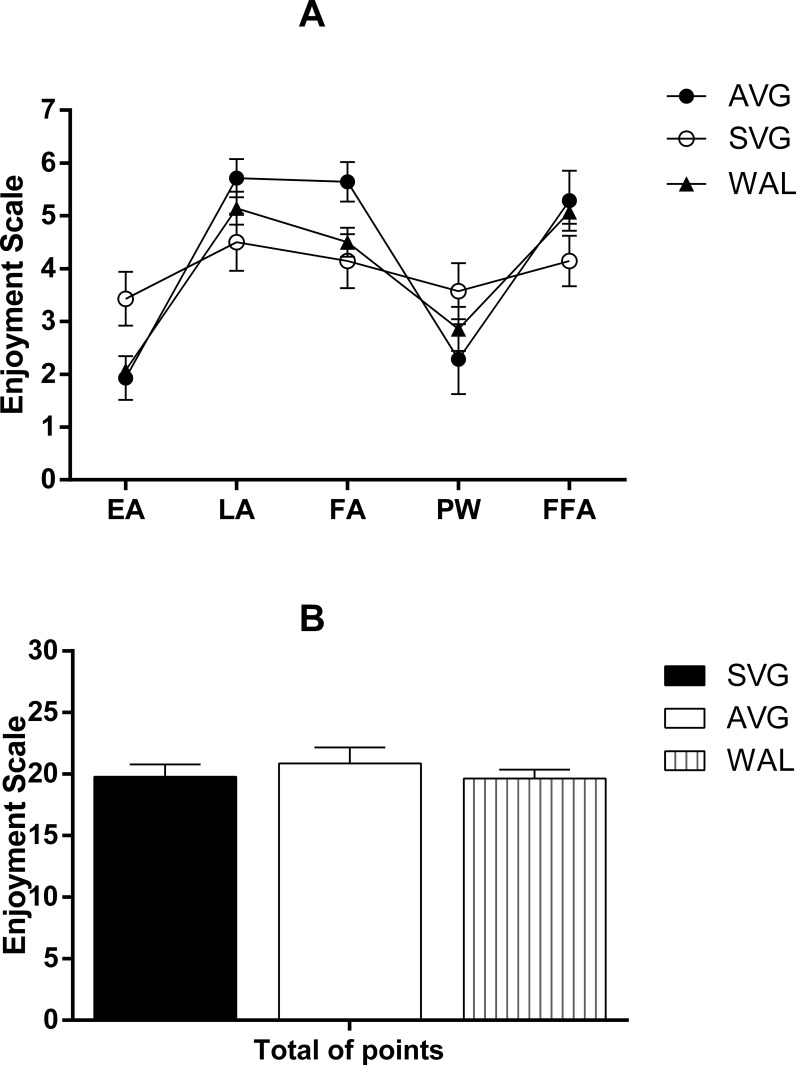
**Values that describe the Enjoyment Scale (A) values obtained in each of the points raised in the scale and (B) the sum of these points for each study session.** EA–enjoy the activity; LA–like the activity; FA–had fun with the activity; PW–physically well to perform the activity; FFA–feel frustrated when performing the activity; Data are mean and standard error of the mean. (N = 14). One-way Anova. No significant differences were found (p> 0.05). Source: Silva, 2015.

The enjoyment shown by study participants in relation to activities carried out is expressed in Fig 5. All sessions promoted a feeling of enjoyment, but no differences were found between the sessions for the variables of scale enjoyment or for the sum of points of all scales.

**Fig 6:**
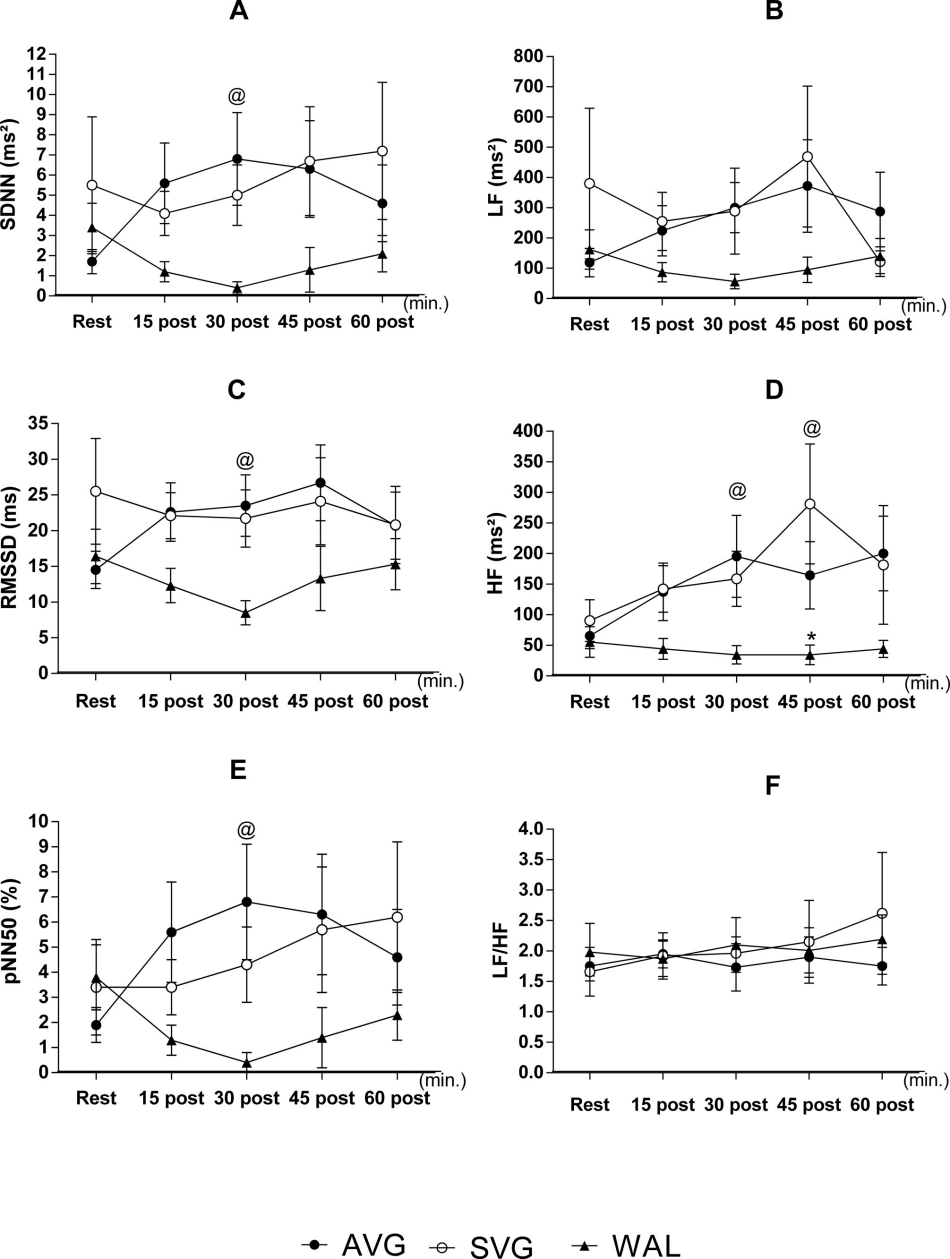
(A) Average standard deviation values of RR intervals (SDNN), (C) the root mean square of successive differences between adjacent R-R (RMSSD) and (E) the percentage difference between successive R-R intervals greater than 50 ms (pNN50), as (B) low values of variables (LF) and (D) high frequency (HF) and (F) the sympathetic-vagal balance (LF/HF) during rest and during activities of the recovery period (60 minutes). Data are mean and standard error of the mean. (N = 8). Repeated measures two-way ANOVA followed by Bonferroni post-test. P <0.05. * = Difference in relation to the SVG; @ = Difference between AVG and WAL. Source: Silva, 2015.

There were sample losses for the variables of the autonomic modulation, so that the analyses were only conducted for eight volunteers who had data collected from all planned moments. For this sample size, it was observed that AVG and SVG had very similar behavior to the autonomic parasympathetic nervous activity indicators in the time domain (SDNN, RMSSD and PNN50) and frequency (HF) in the recovery period after exercise or playing, while WAL resulted in consistently lower values in the same period, although statistical differences were only noted at 30 minutes after exercise between AVG and WAL (Fig 6). Parasympathetic activity was still significantly lower at 45 minutes after WAL recovery in HF regarding both AVG and SVG.

## Discussion

This study showed that an AVG session promotes significant increase in HR, oxygen consumption and energy expenditure in relation to the pre-game resting state and in comparison with SVG session. It was also shown to be comparable to the cardiometabolic behavior of a WAL session, although half of the measurements made, the metabolic demand was higher in WAL. For this cardiometabolic demand, it was observed that a session of AVG is able to promote significant post-game pressure reduction compared to pre-game values and an SVG session and similar hypotension after a walking exercise session.

The pressure reduction promoted by the walking exercise session was descriptively, but not significantly greater than that obtained by AVG session. In the present study, the subjects failed to maintain a moderate WAL session (at least 60% of the maximum RH), although we reported moderate intensity based on the scale of perceived exertion used. This could limit the comparison of reduced pressure occurring in these two modes, but the hypotensive magnitude obtained in this study was similar to what has been observed for the aerobic exercise protocols (walking or running) of moderate intensity that are widely available in the literature. Carvalho et al.[15] found hypotension of 10.3 mmHg for systolic BP and 11.2 mmHg for diastolic BP after a WAL session on a treadmill with 42 minutes duration in the anaerobic threshold intensity. A meta-analysis and review indicated a reduction from 4 to 19 mmHg for systolic component and from 2 to 9 for the diastolic component in response to an aerobic workout [16–18], including those values that were obtained in this study for AVG.

An increased energy demand in the AVG session, yet no significant statistical differences in relation to the session WAL, gives this game an exercise status. It should be noted that the achieved intensity was short of what can be considered moderate, since the volunteers reached 32.4 ± 4.3% of maximum HR. The consensus that has been formed by previous studies on post aerobic exercise hypotension is that this phenomenon is best observed after moderate-intensity exercise. However, in the review by Annunciation and Polito [19], which considered studies that adopted intensities between 30% and 75% of maximal aerobic capacity and a duration of 15–50 minutes of aerobic exercise, there was post-exercise hypotension for systolic BP and BP diastolic even for the lower intensities, which includes the intensity reached by the volunteers of this study in AVG. These data lead us to understand the AVG as a game that is able to promote comparable metabolic demand to systematic exercise sessions that are traditionally practiced by the population.

It should be noted, however that even though volunteers performed WAL at only 32% of maximal heart rate, this intensity was still higher than that observed in AVG in half of the measures measured. This confirms the premise that AVG practiced even at low intensity is able to promote acute reduction of post-game blood pressure.

The entertainment notoriously promoted by electronic games could act as an intervening variable, mistaking the pressor responses obtained in this study. However, it was noted that the feeling of pleasure shown by hypertension in the present study did not differ between video games and the walking exercise. In addition, the SVG procedure did not cause any hypotensive response, despite having promoted the same sense of AVG pleasure. Thus, we can maintain the proposition that the increased metabolic demand promoted by AVG is what really promoted the post game hypotensive response.

As we have demonstrated AVG's ability to promote reduction in BP, future investigations are required to determine the mechanisms involved in this phenomenon. Reduced sympathetic nerve activity, increased parasympathetic activity, increased histamine H1 and H2 activity and decreased cardiac output [20] are among the most accepted mechanisms. In our study, we tried to anticipate assessing autonomic nervous activity through cardiac autonomic modulation. In addition to the sample loss which occurred, the implemented method is limited, since it indirectly checks the modulation addressed to the heart, when the sympathetic activity to the vessels could best explain the pressure behavior. Either way, the data provide evidence that the autonomic modulation does not seem to participate in the pressure reduction promoted by the AVG game.

The absence of a washout for medication is a limitation of this study to evidence the antihypertensive role of active video games in antihypertensive treatment without the confounding agent of the medication. Our laboratory has tried to minimize the ethical difficulty to request abstinence from medication on the days of the experimental procedure by standardizing the time between the last medication and the beginning of the experimental procedures for the longest time possible. Considering the routine of the people (time to wake up, take medication and practice exercise) we were able to establish six hours between the medication and the experimental treatments.

The data obtained from this study allow us to suggest that AVG can be considered as a clinically relevant option for hypertension treatment. Furthermore, the observed increase in energy demand (similar to mild walking exercises) is not only useful in the prevention of hypertension [21], but also in other chronic diseases [22].

The practical implication of the findings in this study depends on the people’s adherence to practice AVG. Being that AVG is an activity associated with entertainment, it is expected that it has the capacity to promote loyalty among adult users. The possibility of the game to be played at home can mean the elimination of the barriers reported by people to exercise [23]. However, adult adherence to daily AVG practice even knowing the health benefits still needs to be investigated, since this type of game is more practiced by children and adolescents. Also, it is still necessary to check the effect of the everyday AVG practice in chronic response of BP to better determine the antihypertensive clinical potential of these games. Another suggestion regarding future studies is the use of other games, since the data from this study can only be considered precise regarding the Dance Central game.

## Conclusion

Active video games can be considered as a tool with important therapeutic power since it resulted in similar behavior to walking, and was different from traditional video games, in regards to physiological demand and blood pressure responses in hypertensive women. However, investigations need to be developed in order to observe whether its users will really practice it, because of video game availability at home, and given this therapeutic power. Moreover, it is necessary to observe the effects of daily video game practice, not only with the game used in our study, but with other types of active games in order to determine the antihypertensive potential of these games.

## Supporting information

S1 Text Consort 2010 flow diagram. (DOC)

S2 Text Trial study protocol. (DOCX)

S1 Table Raw data of the study. (XLSX)
