# Supplementary material for: Energy demand in an active videogame session and the potential to promote hypotension after exercise in hypertensive women
Source: PLoS One. 2018 Dec 13;13(12):e0207505. doi: 10.1371/journal.pone.0207505 (PMC6292658; doi:10.1371/journal.pone.0207505)
Supplement: S2 Text — (DOCX) [file pone.0207505.s002.docx]

UNIVERSIDADE FEDERAL DA PARAÍBA

PROGRAMA ASSOCIADO DE PÓS-GRADUAÇÃO EM EDUCAÇÃO FÍSICA

UPE/UFPB

**Eficácia do vídeo game ativo na pressão arterial e glicemia em hipertensas diabéticas: efeitos agudos e crônicos**

JOÃO PESSOA

2014

TAÍS FEITOSA DA SILVA

Trabalho apresentado ao Comitê de Ética em Pesquisa, sob orientação do Professor Dr. Alexandre Sérgio Silva para desenvolvimento de projeto de pesquisa no Laboratório de Estudos do Treinamento Físico Aplicado ao Desempenho e à Saúde - LETFADS, departamento de Educação Física

**Eficácia do vídeo game ativo na pressão arterial e glicemia em hipertensas diabéticas: efeitos agudos e crônicos**

**Orientador: Alexandre Sergio Silva**

JOÃO PESSOA

2014

1. **INTRODUÇÃO**

É um consenso na literatura a importância da prática da atividade física realizada de maneira regular para a saúde humana (HASKELL et al., 2007; SBC, SBH, SBN, 2010; HENRIQUES, 2013). Entretanto, o desenvolvimento de recursos tecnológicos tem contribuído para a prática do comportamento sedentário, inclusive no âmbito do lazer, onde atividades como jogar vídeo game substituem atividades como a prática de esportes (PITANGA e LESSA, 2005). No entanto, surgiu uma tendência do mercado para o desenvolvimento de jogos de vídeo ativos, ferramenta que pode ser considerada alternativa à prática de atividades de lazer sedentário (STAIANO e CALVERT, 2011).

O vídeo game ativo (VGA), também denominado *exergame* ou vídeo game fisicamente interativo, é conceituado como um jogo em que o praticante substitui o *joystick* tradicional por um sensor que o leva a realizar os movimentos que deseja que sejam realizados pelo *avatar* existente na tela (STAIANO e CALVERT, 2011). O gasto energético resultante da prática destes jogos é aumentado de maneira importante quando comparado ao vídeo game sedentário (VGS) conforme tem sido demonstrado em crianças, adolescentes (GRAVES et al., 2008; PEREIRA et al., 2012), adultos jovens e idosos (GRAVES et al., 2010).

Apesar da maior demanda energética do VGA quando comparado ao VGS e de sua semelhança com o exercício físico, esta demanda ainda se situa abaixo ou nos limites mínimos do recomendado pelas principais associações que estudam o exercício físico (HASKELL et al., 2007). Enquanto estas instituições recomendam exercícios com intensidade entre moderada e vigorosa, o que seria em torno de 60% e 85% da frequência cardíaca máxima (FCM), os VGA’s impõem, na maioria dos estudos, uma intensidade abaixo de 55% da FCM (GRAVES et al., 2008; O’DONOVAN e HUSSEY, 2012; O’DONOVAN et al., 2012; MELLECKER e MACMANNUS, 2013; MILLS et al., 2013) e apenas em alguns estudos se atingiu intensidade superior a 60% da FCM, não passando de 75% da FCM (SIEGEL et al., 2009; O’DONOVAN e HUSSEY, 2012).

Por causa da baixa demanda fisiológica destes jogos, estudos têm sido conduzidos para testar o poder terapêutico dos VGA´s com pessoas com baixa aptidão física. Em estudos de Broeren et al. (2008) e Garcia et al. (2010) ocorreu melhoria do desempenho motor quando o VGA foi adotado na reabilitação fisioterapêutica após acidente vascular encefálico em adultos e idosos. Além disso, estudo de Maddison et al. (2011) que avaliaram a prática destes jogos, durante 24 semanas, sobre a composição corporal em adolescentes com sobrepeso e obesidade, mostraram redução do índice de massa corporal (IMC) e da gordura corporal e o estudo de Madsen et al. (2007) que também avaliaram adolescentes com sobrepeso durante o mesmo período de prática, não encontraram redução do IMC.

Do ponto de vista do tratamento da hipertensão e da diabetes, sabe-se que a intensidade é uma variável de grande importância. Entende-se que os melhores efeitos de redução da pressão arterial (PA) são observados em exercícios realizados inicialmente com intensidade entre 60% e 80% da FCM, enquanto que para o controle da glicemia são necessários exercícios realizados com intensidade entre 50% e 80% da FCM, conforme as diretrizes de tratamento destas doenças (SBC, SBH, SBN, 2010; SBD, 2013/2014).

Apesar das recomendações para o tratamento da hipertensão proporem uma intensidade mínima de 60% da FCM (SBC, SBH, SBN, 2010), existem estudos onde se demonstra que exercícios com intensidades mais leves são suficientes para promover redução da PA, como é apontado no estudo de Anunciação e Polito (2010), que afirmou que exercício físico aeróbio realizado em intensidade de aproximadamente 30% da FCM promoveu hipotensão pós exercício (HPE). Acerca da diabetes, convém notar que é possível encontrar jogos de VGA que imponham a demanda mínima necessária para que se promova redução da glicemia. Estes pressupostos oferecem as bases que fundamentam a hipótese de que uma sessão de VGA é capaz de promover HPE e um programa de treinamento baseado em jogos de VGA é capaz de promover redução da PA de repouso e da glicemia em mulheres hipertensas e diabéticas.

Sabe-se que diversos marcadores influenciam no controle destas doenças, como o sistema nervoso autônomo, que está relacionado ao débito cardíaco e resistência vascular periférica (DE ANGELIS et al., 2004; MOSTARDA et al., 2009), além de ser um responsável pela ação dos órgãos vitais como o pâncreas que produz a insulina (REFERÊNCIA). Outro influenciador é a ocorrência do estresse oxidativo que se caracteriza como um desbalanço no sistema redox que leva a danos nos vasos sanguíneos e órgãos importantes (GOTTLIEB et al., 2010). Assim como estes, a inflamação sistêmica é um ponto em comum das doenças crônicas, uma vez que estas se originam de uma inflamação no organismo (VOLP et al., 2008).

Quando relacionada a prática do exercício físico a estes marcadores, pode-se encontrar estudos que mostram uma modulação da atividade autonômica cardíaca após o exercício (LUNZ et al., 2013), assim como uma regulação da glicemia sanguínea independente da produção de insulina (ARSA et al., 2009), além de uma redução do estresse oxidativo, com o aumento de substâncias antioxidantes circulantes (ZANELLA et al., 2007) e redução dos marcadores da inflamação sistêmica (LEANDRO et al., 2002). Sendo assim, neste estudo também será testada a hipótese de que o VGA pode modificar o comportamento destes marcadores, podendo explicar possíveis HPE e redução da glicemia em resposta a sessões ou treinamento com VGA.

Portanto a proposta deste estudo é avaliar a eficácia de uma sessão e de um programa de treinamento com VGA sobre a PA, modulação autonômica cardíaca, perfis glicêmico e lipídico, inflamação sistêmica e estresse oxidativo em mulheres hipertensas diabéticas.

1. **OBJETIVOS**

**2.1 Geral**

Investigar a eficácia de uma sessão e de um programa de treinamento com VGA sobre a pressão arterial e perfil glicêmico de hipertensas diabéticas avaliando alguns aspectos norteadores, sendo eles, demanda fisiológica, modulação autonômica cardíaca, perfil lipídico, inflamação sistêmica e estresse oxidativo.

**2.2 Específicos**

- Avaliar a demanda energética e a sensação de prazer em uma sessão com VGA e no início, meio e fim de um programa de treinamento;
- Avaliar a reposta da pressão arterial clínica a uma sessão de VGA;
- Analisar a resposta pressórica clínica e ambulatorial de hipertensas diabéticas a um treinamento com VGA;
- Analisar a resposta da modulação autonômica cardíaca de hipertensas diabéticas após uma sessão com VGA e no início, meio e fim de um programa de treinamento;
- Analisar reposta glicêmica e hemoglobina glicada de hipertensas diabéticas no início, meio e fim de um programa de treinamento com VGA;
- Analisar o efeito de um treinamento com VGA nos marcadores inflamatórios sistêmicos;
- Analisar o efeito de um treinamento com VGA nos marcadores de estresse oxidativo.

1. **PERGUNTA DO ESTUDO**

Uma sessão e/ou um protocolo de treinamento com VGA são capazes de promover redução da PA, modulação autonômica cardíaca, redução da glicemia, da inflamação sistêmica e modificar marcadores de estresse oxidativo em hipertensas diabéticas?

1. **HIPÓTESES**

**H0:** Uma sessão com VGA NÃO é capaz de promover modificações na demanda energética ou sensação de prazer ou HPE ou modulação autonômica cardíaca em hipertensas diabéticas.

Um programa de treinamento com VGA NÃO é capaz de promover redução da PA de repouso ou da modulação autonômica cardíaca ou controle da glicemia ou modificações inflamatórias de hipertensas diabéticas.

**H1:** Uma sessão com VGA é capaz de promover modificações na demanda energética ou sensação de prazer ou HPE ou modulação autonômica cardíaca em hipertensas diabéticas.

Um programa de treinamento com VGA é capaz de promover redução da PA de repouso ou da modulação autonômica cardíaca ou controle da glicemia ou modificações inflamatórias de hipertensas diabéticas.

1. **REVISÃO DE LITERATURA**

*5.1* *Eficácia do exercício físico para tratamento da hipertensão arterial*

A hipertensão arterial é uma condição na qual há permanente aumento dos valores pressóricos sistólicos e diastólicos (SBC, SBH, SBN, 2006), com prevalência de aproximadamente 7,6 milhões de mortes no mundo (WILLIAMS, 2010). O tratamento desta doença envolve medidas farmacológicas e não-farmacológicas (MORAIS et al., 2011). Entre os métodos não-farmacológicos, o exercício físico tem sido indicado como a mais importante intervenção para prevenção, tratamento e controle da PA elevada (MACDONALD et al., 2002; MORAIS et al., 2011).

A prática do exercício físico tem importante ação sobre o controle da PA para manutenção de valores considerados normais. Esta ação ocorre logo ao final da realização de uma sessão de exercício físico, onde os valores pressóricos diminuem e permanecem abaixo dos valores pré-exercício, podendo esta redução perdurar por até 22h, sendo denominada na literatura como hipotensão pós exercício (HPE) (LATERZA, RONDON e NEGRÃO, 2007). A redução pressórica pode ser observada em pessoas normotensas, mas principalmente em pessoas hipertensas, como encontrado na revisão de literatura de Casonatto e Polito (2009) que reuniu 53 artigos resultantes de que a literatura confirma a HPE em hipertensos e pré-hipertensos e com menor magnitude em normotensos.

A literatura traz que a redução da pressão arterial sistólica (PAS) e diastólica (PAD) em hipertensos após uma sessão de exercício físico, pode atingir de 18 a 20 mmHg e de 7 a 9 mmHg, respectivamente, para PAS e PAD (KENNEY e SEALS, 1993), embora estudos mais recentes tenham mostrado valores menores, como encontrado em estudo de revisão, uma redução da PAS de 3,84 mmHg e da PAD de 2,58 mmHg (ASH et al., 2013).

A redução da PA pode ser influenciada a partir de diversos fatores relacionados ao exercício físico, como valor de PA inicial, modalidade, intensidade e duração (MACDONALD, 2002). Em se tratando dos valores iniciais de PA, está claro na literatura que, quanto maior o valor de PA de repouso, maior é a queda pressórica após o exercício, como observado no estudo de Pescatello et al. (1991), que avaliou hipertensos e normotensos após exercício aeróbio, onde o grupo de hipertensos reduziu 6 mmHg de PAS e 9 mmHg de PAD após o exercício, enquanto do grupo de normotensos aumentou a PAS, em 5 mmHg e reduziu a PAD em 2 mmHg.

Quanto ao tipo de exercício físico, os aeróbios são reconhecidos de forma consensual acerca de seus efeitos sobre a redução da PA, sendo os mais utilizados como forma de tratamento não farmacológico da hipertensão (BERMUDES et al., 2003; PESCATELO et al., 2004). A eficácia do exercício aeróbio é encontrada no estudo de Muniz, Manuchaquian e Andrade (2010) que avaliou 11 hipertensos de meia idade que realizaram uma sessão de exercício aeróbio com duração de 30 minutos e foram avaliados por 30 minutos após o exercício. Os resultados foram redução da PAS de 10 mmHg e PAD de 6 mmHg ao final do período de recuperação.

Outro tipo de exercício físico é o de força que vem sendo estudado quanto a redução da PA sobre hipertensos (CORNELISSEN e FAGARD, 2005). Em estudo de Melo et al (2006), com hipertensas medicadas, que realizaram exercícios de musculação, em que avaliaram a PA por até 21h, foi encontrada uma redução média da PAS de 12,0±3,0 mmHg e da PAD de 6,0±2,0 mmHg, respectivamente, nos primeiros 120 minutos de recuperação, e a média de PA ao longo das 21h foi menor no grupo exercício (123,0±4,0 mmHg), quando comparada ao grupo controle (128,0±5,0 mmHg).

Além destas modalidades, existem outras menos estudas, mas que podem influenciar no controle da PA, como a prática recreacional de uma partida de futebol, que promoveu redução de 13,8±11,0 mmHg (PAS) e 8,8±5,0 mmHg (PAD), em hipertensos de meia idade (NÓBREGA et al., 2013). Outra modalidade que tem sido bem estudada é a *yoga*, sendo abordada como eficaz na redução da PA de hipertensos (TYAGI e COHEN, 2014). Entretanto, entre os estudos encontrados, a avaliação desta modalidade é realizada de maneira crônica, mostrando uma redução da PAS de 4,17 mmHg e da PAD de 3,26 mmHg (HAGINS et al., 2013). O estudo de Wolff et al. (2013), onde foi realizada a monitorização de 24h de PA após uma sessão de *yoga*, mostrou redução da PAD (4,4 mmHg), não mostrando alterações na PAS.

Uma atividade que tem sido associada com a manutenção da PA é a prática do VGA. No entanto apenas dois estudos foram encontrados na literatura, o primeiro estudo foi realizado com crianças normotensas, trazendo uma tendência a redução da PA após a realização de uma sessão de VGA (RAUBER et al., 2013), o que leva-nos a pensar que esta redução pode ser mais evidente na população hipertensa. O segundo estudo foi realizado com jovens saudáveis (21,0±1,6 anos) e não investigou a HPE, mas comparou os valores de PA obtidos imediatamente após quatro tipos de jogos com o repouso, resultando em mais demanda do sistema cardiovascular, variando a PAS de 110 a 140 mmHg e a PAD de 60 a 80 mmHg para a, entre os tipos de jogo (PERRIER-MELLO et al., 2014).

Em se tratando da intensidade e da duração com que o exercício é realizado, o mais recomendado para o tratamento anti-hipertensivo são os exercícios de intensidade moderada que, de acordo com as VI Diretrizes Brasileiras de Hipertensão, estão entre 60% e 80% da FCM (SBC, SBH, SBN, 2010), e duração de 30 a 60 minutos (FORJAZ et al., 2004). No entanto, artigos de revisão trazem que intensidades tão diversas quanto 40% a 90% da capacidade máxima e durações entre 10 e 170 minutos, como mostrado no estudo de revisão de Casonatto e Polito (2009), levaram a ocorrência de HPE. Outro estudo de revisão encontrou que intensidades entre 30% e 75% da capacidade máxima e duração de 15 a 50 minutos levaram a redução da PAS e/ou PAD (ANUNCIAÇÃO e POLITO, 2010).

Contudo a magnitude e a duração da redução da PA podem variar de acordo com a intensidade e a duração do exercício. Quanto a intensidade, alguns estudos trazem que exercícios realizados em intensidades mais elevadas geram redução da PA por maior período de tempo, quando comparado a exercícios de baixa e moderada intensidade (PIEPOLI et al., 1994; FORJAZ et al., 2004). Acerca deste tema, está existindo uma tendência de realização de sessões de exercício de alta intensidade, onde Brito et al. (2014) mostraram que uma sessão de exercícios de fortalecimento muscular de alta intensidade (80% de 1 RM) levou a redução significativa de 33 mmHg (PAS) e 15 mmHg (PAD), enquanto que a sessão de intensidade moderada (50% de 1RM) reduziu 23 mmHg (PAS) e 7 mmHg (PAD).

Quanto a influência da duração do exercício, alguns autores afirmam que exercícios de curta duração geram menor redução da PA por menos tempo, quando comparado ao exercício mais longos (FORJAZ, 1998). No entanto, Guidry et al. (2006), que realizaram seu estudo com homens hipertensos (43,4±1,5 anos), encontrou redução da PAS de 5,6±2,0 mmHg e 4,3±1,6 mmHg, respectivamente, nas sessões com curta (15 minutos) e longa duração (30 minutos) e intensidade de 40% do consumo máximo de oxigênio e 4,1±1,6 mmHg e 4,9±1,9 mmHg, nas mesmas sessões, porém com intensidade de 60% do consumo máximo de oxigênio. Para PAD as reduções foram 2,1±1,0 mmHg (15 minutos) e 3,6±1,4 mmHg (30 minutos) em 60% e 2,4±1,0 mmHg (30 minutos) em 40%. Sendo assim, a relação intensidade vs. duração parece ser mais determinante na HPE do que a ação isolada de tais variáveis (JONES et al., 2007).

Tendo em vista todas estas evidências de que uma sessão de exercício físico é eficaz na redução da PA, quando é realizado um treinamento físico, o efeito do exercício se dá na redução da PA de repouso, com a realização continuada da prática do exercício físico, sendo utilizado como forma de tratamento não farmacológico da hipertensão (ARAÚJO, 2001; HAMER, 2006). Tem sido demonstrado que o treinamento físico provoca alterações autonômicas e hemodinâmicas como a redução da atividade nervosa simpática, da resistência vascular periférica e do débito cardíaco, fatores que influenciam na redução da PA (HALLIWILL et al., 1996; REZK et al., 2006; HAMER, 2006).

A literatura mostra evidências importantes da eficácia do treinamento físico de diferentes modalidades sobre a PA, descrevendo reduções sistólicas de repouso de 3,8 a 11 mmHg, e diastólicas de 2,6 a 8 mmHg em hipertensos, após treinamento aeróbio de pelo menos duas semanas (HALBERT et al., 1997; HAGBERG et al., 2000; WHELTON et al., 2002). Em estudo de LIU et al. (2012), onde foi realizado um treinamento aeróbio de oito semanas, com frequência semanal de quatro vezes e sessões de 30 minutos a 65% do consumo máximo de oxigênio, foi encontrada redução de 7,0±1,4 mmHg (PAS) e 5,2±1,2 mmHg (PAD), em pré-hipertensos.

Quanto ao treinamento de força, Nascimento et al. (2014) que realizaram um estudo com duração de 14 semanas, frequência semanal de duas sessões e duração de 15 minutos em intensidade moderada, em mulheres hipertensas, mostrou redução da PAS (-18 mmHg) e PAD (-10 mmHg) ao final do treinamento. Já quanto ao treinamento intervalado de alta intensidade, o estudo de Dall et al., (2014) que avaliaram transplantados cardíacos, mostrou uma redução da PAS (-5,2 mmHg) após 12 semanas (3Xsemana), em intensidade de 80% do VO2máx, intervalos de 2 minutos em intensidade de 60% do VO2máx e duração de 32 minutos/sessão.

Assim como estas, uma modalidade alternativa que tem sido investigada, o *tai-chi-chuan*, também tem sido relacionado com a redução de PA, onde Tsai et al. (2003) ao avaliarem a PA de pré-hipertensos e hipertensos antes e após 12 semanas de *tai-chi-chuan*, sendo três sessões semanais com duração de aproximadamente 50 minutos, encontraram redução da PA de repouso de 15,2 mmHg (PAS) e 8,8 mmHg (PAD). Diante destas evidências fica clara a relação de eficácia entre a realização de exercício físico e o controle da PA, sendo esta uma importante ferramenta a ser utilizada no tratamento de pessoas acometidas pela hipertensão arterial.

*5.2 Eficácia do exercício físico para tratamento do diabetes mellitus tipo II*

O diabetes mellitus tipo II (DMII) é uma das doenças crônicas mais frequentes e que tem tido um aumento da prevalência em todo o mundo (DORNAS, OLIVEIRA e NAGEM, 2011). No ano de 2013, o Brasil se encontrou como o quarto país do mundo em número de casos, com quase 13,4 milhões de casos (SBD, 2013-2014). Ao ocorrer ingestão de glicose, a ação da insulina é ligar-se a receptores para mediar a entrada de glicose nas células. Quando a insulina não é produzida ou quando seus receptores ficam dessensibilizados, levando a hiperinsulinemia e consequente resistência à insulina, essa ação não ocorre e a glicose se acumula na corrente sanguínea, gerando a hiperglicemia. (SBD, 2013-2014).

Há evidencias de que alterações no estilo de vida, incluindo a redução dos níveis de atividade física e prática do comportamento sedentário estão associados a acentuado incremento da prevalência da DMII, além disso, esta doença vem geralmente acompanhada por outras patologias crônicas como hipertensão e obesidade. Os programas de prevenção e tratamento primário do DMII baseiam-se em intervenções na rotina alimentar e na prática de atividade física. (SDB, 2013/2014). Resultados do *Diabetes Prevention Program* mostram uma redução de 58% da incidência de casos da DMII mediante o estimulo a uma dieta saudável e prática de atividades física, sendo mais efetiva que o uso de fármacos (BARCELÓ et al., 2003)

O exercício físico apresenta alguns efeitos importantes que influenciam na resistência à insulina, como a redução dos lipídios acumulados, atuação em mediadores inflamatórios, e transporte da glicose e redução do estresso oxidativo (BASSUK e MANSON, 2005). Assim, a prática do exercício estabiliza a glicose sanguínea, reduz resistência à insulina e a hemoglobina glicada em pacientes com DMII, (DELA et al., 1995; BOULÉ et al., 2001; O’HAGAN, DE VITO e BOREHAM, 2013). Sabe-se que a redução de 1% de hemoglobina glicada foi associada a diminuição de 37% dos riscos de complicações microvasculares e reduziu 21% das mortes (STRATTON et al., 2006), por isso o exercício físico é considerado o elemento chave para essa condição (COLBERG et al., 2010).

O tipo de exercício físico mais recomendado pelas diretrizes de saúde são os aeróbios que envolvem grandes grupos musculares, de intensidade moderada (50% da FCM) a vigorosa (>70% da FCM) e frequência semanal de três a sete dias/semana não consecutivos. Os de fortalecimento muscular (resistidos) devem ser multi-articulares, sendo de duas a quatro séries de oito a dez repetições, de intervalo entre as séries de um a dois minutos, em uma sessão contendo cinco a dez exercícios direcionados para os principais grupos musculares, com intensidade de oito a dez repetições máximas. A frequência semanal deve ser de duas a três vezes/semana não consecutivos. Os exercícios de flexibilidade devem ser realizados de forma complementar a estas modalidades (MARWICK et al., 2009; COLBERG et al., 2010; SBD, 2014)

É possível perceber a ação do exercício sobre marcadores da DMII já após uma sessão de exercício físico, com o aumento do consumo de glicose que gera um efeito hipoglicemiante saudável (MERCURI e ARRECHEA, 2001). Os resultados de Van Dijk et al. (2013) que avaliaram 60 diabéticos por 24h após uma sessão de exercício aeróbio (50% da capacidade máxima), foi redução da glicemia média (-16,2 mg/dL) e a não ocorrência de hiperglicemia em 31% dos casos. O estudo de Oberlin et al. (2014) que avaliaram nove diabéticos após uma sessão de 60 minutos de exercício aeróbio a 75% da FCM, mostrou redução da glicose plasmática durante as 24h e redução da glicose pós prandial durante 48h após a sessão.

Van Dijk et al. (2012) também avaliaram 30 diabéticos após uma sessão de força (45 minutos) que levou a redução da glicose sanguínea de forma significativa (de 133,2 mg/dL para 122,4 mg/dL) e da prevalência de hiperglicemia de 35% para 33%, quando comparado ao grupo controle. Já uma sessão de exercícios combinados (aeróbio e de força) resultou em redução da glicemia capilar (179,03 ml/dL para 148,04 ml/dL) (SILVA e LIMA, 2002). Figueira et al. (2013), trouxeram uma redução da glicemia média de 24h tanto após uma sessão de exercício aeróbio (de 151,0±8,0 mg/dL para 124,0±9,0 mg/dL) quanto após uma sessão de exercícios combinados (aeróbio e força) (de 147,0±9,0 mg/dL para 125,0±6,0 mg/dL).

No entanto, para que o exercício seja considerado ferramenta eficaz no tratamento desta doença, os resultados devem ser somados em sessões de treinamento (DUCLOS et al., 2011). A literatura confirma a eficácia do treinamento aeróbio para controle da DMII. Monteiro et al. (2010) avaliaram o efeito de 13 semanas de treinamento aeróbio de caminhada em esteira com duração de 50 minutos/sessão, frequência semanal de três vezes em intensidade de 60% a 80% da FCM, em idosas diabéticas e mostrou redução da glicemia capilar (de 175,3±73,8 mg/dL para 105,3±25,8mg/dL).

Outro estudo que investigou a prática do treinamento aeróbio em diabéticos foi de Bacchi et al. (2012) que compuseram sua amostra de 19 diabéticos que realizaram três sessões/semana com duração de 60 minutos, durante quatro meses e obtiveram redução das concentrações de hemoglobina glicada (-0,40%) e da glicemia (-15,2 mg/dL). Da mesma forma, estudo de Sigal et al. (2007) que avaliaram a resposta ao treinamento aeróbio por seis meses, com frequência de quatro vezes/semana, em diabéticos tipo II de 39 a 70 anos, obteve redução das concentrações da hemoglobina glicada (-0,38%).

Quanto aos exercícios de força, sabe-se também de sua eficácia, porém como tipo de exercício complementar aos aeróbios (SBD, 2013/2014). No entanto existem estudos na literatura que mostram seus efeitos sobre variáveis relacionadas à diabetes, sem que sejam acompanhados dos exercícios aeróbios. Embora a literatura traga que intervenções com 12 semanas são eficazes por melhorar o controle glicêmico, estudos mostram que em oito semanas de treinamento resistido pode-se perceber uma redução de hemoglobina glicada (O’HAGAN, DE VITO e BOHERAM, 2013)

Baldi et al. (2003) avaliaram nove diabéticos durante o treinamento resistido de dez semanas, sendo três sessões/semana, com dez exercícios por sessão, e intensidade progressiva de 10 RM para 15 RM, e foi encontrada uma redução não significativa de 8,9±0,8% para 8,4±0,6% de hemoglobina glicada. No entanto, estudos mais longos também mostram resultados interessantes, como pode ser visto no estudo de Castaneda et al. (2002), onde 31 participantes realizaram treinamento resistido por 16 semanas, sendo três sessões semanais, cinco exercícios por sessão entre 60% e 80% de 1RM, que reduziram também a hemoglobina glicada (de 8,7±0,3% para 7,6±0,2%).

Apesar dos resultados mostrados nos estudos acima possuírem relevância, vale ressaltar que estes exercícios podem ser mais efetivos se realizados de maneira combinada (PRAET et al., 2006). Em estudo mais recente, Sigal et al. (2007) comparam três tipos de treinamento (aeróbio, resistido e combinado) durante seis meses, resultando em uma redução da hemoglobina glicada de 7,46% para 6,99% aos três meses e 6,56% aos seis meses de treinamento combinado. Já o treinamento aeróbio resultou em uma redução de 7,41% para 7,00% aos três meses e 6,98% aos seis meses e o treinamento resistido de 7,48% para 7,35% aos três meses e 7,18% aos seis meses de treinamento.

Além destas modalidades mais estudas, existem as modalidades de exercício menos estudadas que podem ser efetivas como ferramenta de tratamento da DMII, como a *yoga*, onde estudo de revisão mostra modificações da glicose sanguínea após um mês de prática de *pranayma*, um ramo da *yoga* que trabalha respiração (de 148,19±43,13 mg/dL para 108,19±21,05 mg/dL), além de trazer outros estudos mostrando redução da hemoglobina glicada após três meses de prática da *yoga* (-1,96%) e melhora dos receptores de insulina (3,74 ng para 5,81 ng) (SAHAY, 2007). Estudo de Leelayuwat (2013), sugere-se a importância da prática de modalidades alternativas no tratamento da DMII, como o *tai-chi-chuam*, e a dança.

Quanto a estas modalidades, Hung et al. (2009), que avaliaram 28 diabéticos durante 12 semanas (3sessões/semana) praticando tai-chi-chuam, obtiveram redução da glicemia de jejum (-18,0 mg/dL) após a intervenção. Enquanto Manjeri et al. (2014) avaliaram 47 sujeitos diabéticos por seis meses de um programa de aulas de dança (individual e de salão), mostrando ao final do estudo uma redução da hemoglobina glicada (-0,36%).

Outros aspectos importantes acerca da realização do exercício físico como forma de tratamento da DMII é a intensidade utilizada e a duração do exercício, onde a literatura mostra diferentes intensidades abordadas. Estudo de Jeng et al. (2002) que avaliaram a resposta glicêmica após sessões de exercício com diferentes intensidades (40%, 60% e 80% do esforço máximo) e diferentes durações (10, 20, 30 e 40 minutos), resultando em uma significante interação intensidade vs. duração (F = 11.756). Silveira et al. (2014) avaliaram o efeito agudo (10, 20 e 30 minutos após o exercício físico) em intensidade de 60% e 80% de 1RM, resultando em redução da glicose sanguínea de forma similar.

Já meta-análise de Boulé et al. (2003) que avaliaram programas de treinamento de nove estudos com duração média de 20 semanas, 3,4 sessões semanais com intensidades distintas (50% e 75% do consumo de oxigênio máximo), afirmou que exercícios aeróbios de intensidades mais elevadas geram benefícios adicionais aos portadores de DMII como a melhora cardiorrespiratória e das concentrações de hemoglobina glicada. Acerca desta importância, Fex et al. (2014) avaliaram o treinamento intervalado de alta intensidade em diabéticos e percebeu significante redução da glicose sanguínea após 12 semanas. Além disso, o estudo de Gillen et al. (2012) que avaliaram sete diabéticos durante 24 h após uma sessão de exercício intervalado de alta intensidade (60 segundos a 89% do consumo máximo de oxigênio e 60 segundos de recuperação) que levou a redução da glicemia pós-prandial quando comparado ao controle.

Tendo como parâmetros todas as informações acerca da influência do exercício físico na DMII, assume-se o exercício físico cada vez mais como parte fundamental do tratamento e controle desta doença, no entanto deve praticado com regularidade para ter os benefícios continuados.

*5.3 A evolução do vídeo game e sua função como tratamento terapêutico*

O avanço tecnológico e a praticidade da vida moderna propiciam ao homem contemporâneo um estilo de vida sedentário (FLORINTO, GUIMARÃES e CESAR, 2009). O comportamento sedentário, também chamado de tempo sentado (TREMBLAY et al., 2011) tem sido associado ao surgimento de doenças crônico-degenerativas (QUEIROZ et al., 2013). Pessoas que dedicam grande parte do seu dia ao comportamento sedentário possuem risco aumentado de 73% em desenvolver índices antropométricos alterados, dislipidemia e hipertensão arterial (TAVARES et al., 2010).

Por outro lado, a prática de atividade física tem sido recomendada para prevenir e tratar estas doenças (SIGAL et al., 2006; ALVEZ et al., 2007), no entanto, apenas 30,3% dos adultos brasileiros relatam atingir os níveis de atividade física recomendadas em atividades de lazer (MINISTÉRIO DA SAÚDE, 2012). A tecnologia preoculpada com o aumento de doenças relacionadas ao comportamento sedentario e a inatividade física tem proporcionado grande evolução no mundo dos jogos de video game (UNNITHAN, 2006).

Recentemente surgiram os jogos ativos, que também podem ser chamados de *exergames*, jogos interativos e vídeo game ativos e tem sido investigados por pesquisadores que avaliam seu potencial de promoção à saúde (PENG et al., 2012). Os vídeo game ativos (VGA’s) são conceituados como jogos em que o praticante substitui o *joystick* tradicional por um sensor que o leva a realizar os movimentos que deseja que sejam realizados pelo *avatar* existente na tela, onde requerem dos participantes movimentos de segmentos corporais ou de todo o corpo diante da câmera para controle do jogo (STAIANO e CALVERT, 2011; PEREIRA et al., 2012).

Existem vários tipos de VGA, como o *Dance Dance Revolution* (SSD Company Ltd., Shiga, Japão) que utiliza um tapete eletrônico de dança com uma seleção de músicas (LANNINGHAM-FOSTER et al. 2006), o *EyeToy Kinetic* (Sony Computer EntertainmentEurope, Ltd, Londres, Inglaterra) (LANNINGHAM-FOSTER et al., 2006; NI MURCHU et al., 2008), o *XaviX* (SSD Company Ltd., Shiga, Japão), que proporciona atividades esportivas como golfe, tênis, boxe e boliche e inclui um tapete de jogo (*XaviX J-Mat*) permitindo a realização de caminhada ou corrida no mundo virtual (LANNINGHAM-FOSTER, 2006). Além destes, existe ainda o sistema *Nintendo Wii*™ (Nintendo, Consolidated financial high lights), um dispositivo composto por um sensor de movimento e o *Wii Remote*™ que se assemelha a um controle remoto sem fio e permite a execução de atividades desportivas (GRAVES, STRATTON e RIDGERS, 2007).

Outros exemplos de VGA são o *Your Shape Fitness*, o *Just Dance*, o *Zumba,* entre outros, que utilizam o console *Xbox* *Kinect*™ (Foxconn®, Nova Taipé, China), a mais avançada tecnologia, surgida em 2010 e que permite controle completo do corpo dos personagens virtuais animados (BAO et al. 2013), pois captura movimentos para projetar a imagem do jogador em uma tela localizada em um ambiente virtual de jogo (O’DONOVAN et al., 2012; HOLMES et al., 2013), além de ser a preferida dos usuários, provavelmente pela quantidade de movimentos proporcionados por seus sensores (ACSM, 2013; PERRIER-MELO, 2013).

Os estudos mais atuais acerca da utilização dos VGA’s abordam temas como aumento do custo físicológico e dos níveis de ativida de física. O estudo de revisão de Pereira et al., (2012) avaliaram estudos que mensuraram o gasto energético e o nível de atividade física a partir da prática de VGA’s e obtiveram que a prática destes pode ser uma boa opção para o aumento destes recursos em crianças e adolescentes, desde que associados a outros tipos de atividade física. Além disso, dos estudos selecionados, quatro mostraram aumento dos valores de FC durante a prática dos jogos.

Já SIEGEL et al. (2009) que avaliaram o dispêndio energético e a FC em jovens universitários em três tipos diferentes de VGA por 30 minutos e obtiveram 8,10±1,57 kcal.min^-1^ e 159,09±16,96 bpm no simulador de bicicleta, 6,85±2,72 kcal.min^-1^ e 161,04±13,61 bpm no simulador de boxe e 8,62±2,61 kcal.min^-1^ e 166,46±14,28 bpm no simulador de aventuras. Seus resultados mostraram-se aumentados quando comparados aos valores de repouso (1,39±0,33 kcal.min^-1^ e 82,31±11,39 bpm).

Graves et al. (2010) investigaram o dispêndio energético, o consumo de oxigênio e a FC em homens e mulheres saudáveis com idade entre 45 e 70 anos, durante sessões de diferentes tipo de *Nitendo Wii* (*yoga*, condicionamento muscular, equilíbrio, e aeróbica) e resultaram que este jogos promoveram aumento do dispêndio energético (35,5 cal/kg/min; 42,6 cal/kg/min, 36,0 cal/kg/min e 35,8 cal/kg/min, respectivamente) pequeno aumento do consumo de oxigênio (0,57 l/min, 0,68 l/min, 0,57 l/min, e 0,96 l/min, respectivamente), mas não modificaram os valores de FC (83,8 bpm, 86,8 bpm, 84,5 bpm e 94,7 bpm, respetivamente), quando comparado ao repouso (0,32 l/min, 18,7 cal/kg/min e 68,0 bpm).

Apesar de alguns estudos mostrarem uma demanda fisiológica que se assemelha com a prática do exercício físico, esta demanda ainda se situa abaixo ou nos limites mínimos do recomendado pelas principais associações que estudam o exercício físico (HASKELL et al., 2007), além disso, varia muito de um estudo para outro, dependendo ainda, do tipo de jogo. Enquanto estas instituições recomendam exercícios com intensidade entre moderada e vigorosa, o que seria em torno de 60% e 85% da FCM, o estudo de Graves et al. (2008) mostra que o percentual de intensidade atingidas por adolescentes foi de 11% da FCM no Xbox, 24% da FCM no *Wii Bowling*, 27% da FCM no *Wii Tennis* e 49% da FCM no *Wii boxing*.

Ao avaliar sete meninas e oito meninos, Mills et al. (2013) encontraram uma intensidade de 18% da FCM em sessão de VGA. Já Mellecker e Macmannus (2013) que investigaram crianças, encontraram uma intensidade de 44% da FCM no jogo *Gamercize* e 52% no jogo *XaviX*, enquanto O’Donovan et al. (2012) que avaliaram homens saudáveis de 21 anos, obteve uma intensidade de 28% da FCM em uma sessão de *XboX Kinect* *Reller Ridger* e 22% em uma sessão de *Wii Sports Boxing*.

Ainda, em um estudo que avaliou homens de 19 a 27 anos, foi encontrada uma intensidade de 71% da FCM durante uma sessão de *Wii Fit Free* *Jogging*, 58% da FCM durante uma sessão de *Wii Sports Boxing*, 42% da FCM durante o jogo Wii *Sports Baseball* e 42% durante o jogo *Wii* *Sports Tennis* (O’DONOVAN e HUSSEY, 2012). Além deste, Siegel et al. (2009) que avaliaram 33 participantes com média de idade de 26 anos, encontraram uma intensidade de 71% da FCM, durante uma sessão com VGA (contendo três tipos diferentes de jogos).

A intensidade atingida e a demanda fisiológica encontrada ainda podem variar em pessoas com e sem experiência com jogos ativos, como pode ser visto no estudo de Sell, Lillie e Taylor (2013) que ao avaliar 12 universitários com experiência (nível 4 no jogo) e sete sem experiência (nível 1 ou 2 no jogo), em uma sessão com *Dance Dance Revolution* e concluiram que participantes com maior experiência atingiram intensidade mais elevada (161,2±13,8 bpm vs. 95,5±10,5 bpm) e maior gasto energético (10,5±2,0 kcal/min^-1^ vs. 4,8±1,0 kcal/min^-1^.

Por essa grande variação de intensidades e pelo fato de muitos estudos encontrarem uma intensidade leve com a prática do VGA, outros aspectos tem sido abordados em estudos que investigam a prática dos VGA’s com pessoas com baixa aptidão física. Podem ser encontrados estudos com a utilização deste recurso em indivíduos obesos, avaliando variáveis antropométricas, como IMC, pesso corporal, além destes, estudos abordando controle cognitivo, equilíbrio e reabilitações cardíacas.

No estudo de Maddison et al. (2011) onde foi avaliada a prática destes jogos, durante 24 semanas, sobre a composição corporal em adolescentes com sobrepeso e obesidade, e foram encontradas reduções do índice de massa corporal (IMC) e da gordura corporal. Entretanto o estudo de Madsen et al. (2007) que avaliaram 30 crianças e adolescentes de 9 a 18 anos com sobrepeso, durante o mesmo período de prática com Dance Dance Revolution não encontraram redução do IMC.

Em se tratando do controle cognitvo, Kevin et al. (2011) investigaram a eficácia de uma sessão de 20 minutos com *Wii Fit* sobre o controle congnitivo em adultos jovens (18 a 25 anos), encontrando que os VGA’s não são eficázes o controle cognitivo atraves do aumento de recurso da atenção e maior controle de interferência durante atividades cognitivamente exigentes, como é possível perceber em uma sessão de exercício em esteira. Já acerca do equilíbrio, é possível encontrar um artigo de revisão de Perrier-Melo et al. (2013) sobre equilíbrio em idosos, onde ao avaliar oitos estudos que utilizaram o VGA como ferramenta fisioterapêutica, concluiu que os VGA’s são capazes de melhorar o equilíbrio nesta população.

Acerca da reabilitação cardíaca, em estudo de Broeren et al. (2008), onde 11 pacientes com idade média de 68 anos após acidente vascular cerebral, realizaram intervenção de quatro semanas, sendo três sessões/semana com VGA, obtendo melhora no desempenho motor. Assim como este, Garcia et al. (2010) que avaliaram adultos e idosos, também obteve melhoria do desempenho motor após intervenção fisioterapêutica com VGA.

Apenas um estudo investigou a influencia do *Dance Dance Revolution* (sessão com duração de 30 minutos) sobre a PA em crianças com idade média de 9,8 anos, onde ocorreu um aumento significativo de PAS e PA média durante o jogo, quanto comparado a sessão controle (TV) (PAS – 116,9±6,9 mmHg vs. 102,8±7,2 mmHg; PA média – 89,6±4,6 mmHg vs. 81,1±4,9 mmHg) e uma tendência à ocorrência de HPE sistólica (103,5± 4,7 mmHg em repouso e 99,5± 3,7 mmHg aos 40 minutos pós VGA) (RAUBER et al., 2013).

Segundo estudo que avaliou a influencia do VGA sobre a PA, utilizou o console *XboX kinect* e os jogos *Dance Central* e o *Kinect Sports* (boxe, voleibol, tênis e dança), jogados 10 minutos cada um. Foram avaliados oito adultos jovens, e foi encontrado que os quatro tipos de VGA alteraram o sistema cardiovascular, com a PA variando de 110 a 140 mmHg (sistólica) e de 60 a 80 mmHg (diastólica) (PERRIER-MELLO et al., 2014).

No entanto ao estudar grande quantidade de trabalhos utilizando os diversos VGA’s pode-se perceber que a maioria dos estudos realizados utilizam as populações infantil, juvenil e adultos jovens. Poucos estudos abordam a população idosa, e nenhum estudo foi realizado com a população adulta de meia idade, principalmente quando se trata de pessoas acometidas por doenças crônicas como hipertensão e diabetes.

1. **PROCEDIMENTOS METODOLÓGICOS**

*6.1 Tipo de pesquisa:* de acordo com os objetivos traçados, este estudo pode ser caracterizado como sendo do tipo quase experimental, segundo Gaya, 2008.

*6.2 Voluntários do estudo e cálculo amostral:* participarão deste estudo 34 mulheres. Serão adotados como critérios de inclusão: 1) idade entre 45 e 59 anos; 2) apresentar IMC entre 25 e 33 kg/m²; 3) ser hipertensa (apresentar valor inicial mínimo de pressão arterial sistólica de 130 mmHg e diastólica de 90 mmHg; 4) apresentar diagnóstico de diabetes; 5) não praticar previamente exercício físico regular; 6) ser menopausadas (não ter menstruação pelo período mínimo de ano corrido); 7) não ter experiência prévia com VGA de qualquer tipo; 8) não ser acometidas por labirintite. E como critérios de exclusão: 1) iniciar o uso de medicamentos betabloqueadores e bloqueadores dos canais de cálcio durante o estudo; 2) apresentar episódios epiléticos; 4) não ter disponibilidade para realizar todas as sessões do protocolo experimental agudo em um período de dois meses; 5) faltar duas sessões seguidas e/ou mais que 5 sessões (cerca de 20%) do número total de sessões do protocolo experimental crônico.

Para determinação do tamanho da amostra foi realizado o cálculo amostral, utilizando o software *Gpower* 3.0, sendo tomado como base para este cálculo, os dados de um estudo piloto com amostra retirada da mesma população. Foi adotado um erro alfa de 5% e beta de 95%, utilizando-se dados de PAS e PAD com *effect size* de 1,01 e 0,57, respectivamente, chegando assim em um tamanho de amostra de 34 participantes que irão participar da intervenção aguda do estudo. Quanto ao tamanho amostral da intervenção crônica do estudo, não foi possível realizar o mesmo cálculo amostral, uma vez que não foi encontrado na literatura um estudo adequado para isso. Assim, serão realizadas as coletas de dados das primeiras seis participantes, sendo realizado o cálculo amostral a partir destes dados.

As voluntárias serão recrutadas em USF’s próximos ao Campus I da Universidade Federal da Paraíba, a partir da entrada autorizada da pesquisadora, por parte da Secretaria de Saúde da Prefeitura de João Pessoa, uma vez que após aprovado no Comitê de Ética em Pesquisa, a pesquisadora entrará com um processo de solicitação de autorização para este recrutamento. Este projeto será submetido ao Comitê de Ética e após serem esclarecidos todos os procedimentos, as participantes serão orientadas a assinar o Termo de Consentimento Livre e Esclarecido (TCLE) de acordo com a resolução 466/12 do Conselho Nacional de Saúde (apêndice A).

*6.3 Desenho do estudo:* as voluntárias serão inicialmente avaliadas para PA clínica e ambulatorial, modulação autonômica cardíaca (MAC) e variáveis bioquímicas por meio de coletas sanguíneas, além disso, realizarão um teste ergoespirométrico. Em um intervalo entre 48h e cinco dias após a visita inicial, elas realizarão protocolos de jogos de vídeo game ativo e sedentário, dois protocolos de exercícios de caminhada/corrida em esteira e um procedimento controle. Estas sessões serão realizadas em dias separados com intervalo mínimo de 48h e a ordem de realização será randomizada, adotando-se o modelo *crossover*, onde todas as participantes realizarão todas as sessões. Nestes procedimentos, elas serão monitoradas quanto a gasto energético/deslocamentos, sensação de prazer e esforço. Medidas de PA, frequência cardíaca (FC), MAC serão realizadas antes e/ou durante e/ou em um período de recuperação após os procedimentos. Concluída esta fase, as participantes que demonstrarem interesse serão randomicamente divididas em grupo VGA e controle para uma intervenção com duração de oito semanas. Em cada sessão será mensurado gasto energético/deslocamentos. Medidas de PA clínica e MAC serão realizados a cada semana e coletas sanguíneas a cada quatro semanas. Ao final, elas realizarão um novo teste ergoespirométrico e uma nova medida de pressão arterial ambulatorial.

*6.4 Preparação dos sujeitos:* ao aceitarem participar do estudo, será aplicada uma anamnese para a aquisição de informações pessoais e sobre fármacos utilizados e serão coletadas informações antropométricas (estatura, massa corporal e circunferência da cintura) para caracterização dos sujeitos (apêndice B) e será aplicado o questionário IPAQ (*International physical Activity Questionnaire*), utilizado para mensurar o nível de atividade física das participantes (apêndice C). Além disso, será realizado um inquérito nutricional e as participantes receberão uma lista contendo alimentos e medicamentos ricos em cafeína (apêndice D) e serão orientadas a não ingerir os componentes desta lista por 24h, e bebida alcoólica por pelo menos 48 horas antes das sessões do protocolo de intervenção aguda, e antes das medidas de MAC e MAPA durante o protocolo de intervenção crônica.

*6.4.1 International physical Activity Questionnaire (IPAQ):* para determinar o nível de atividade física das participantes será utilizado o Questionário Internacional de Atividade Física/IPAQ-versão curta (MATSUDO et al., 2001), considerado um instrumento que demonstra ter validade e reprodutibilidade similares a de outros instrumentos usados internacionalmente para medir o nível de atividade física (BENEDETTI et al., 2007). Seguindo as recomendações da Organização Mundial de Saúde (OMS, 1995), as mulheres que serão recrutadas deveram apresentar classificação de fisicamente inativas (aquelas que acumulam menos de 150 minutos de atividades leves, moderadas ou intensas por semana).

*6.4.1 Inquérito nutricional:* o consumo alimentar foi avaliado através do Recordatório de 24 horas (apêndice E) e do Questionário de Frequência de Consumo Alimentar (anexo A) aplicados no início e durante o estudo.

O Recordatório de 24 horas consiste em definir e quantificar todos os alimentos e bebidas ingeridas no período anterior ao da entrevista, que pode ser de 24 horas precedentes ou, mais comumente, o dia anterior (GIBSON, 1990). Esse será aplicado três vezes com cada indivíduo, sendo dois representativos da alimentação referente a dias da semana, e um indicativo do consumo alimentar do final de semana. As análises serão feitas mediante a utilização da média dos três valores para investigar o consumo de macro e micronutrientes, com ênfase na presença de substâncias antioxidantes da dieta. Para análise de adequação alimentar, os valores obtidos serão comparados com o preconizado pelas *Dietary Reference Intakes* (DRI’s,) (2002) (OTTEN; HELLWIG; MEYERS, 2006). Esse instrumento será aplicado e avaliado por nutricionista, utilizando-se o software *Avanutri Revolution* versão 4.0 (Avanutri Informática Ltda, Rio de Janeiro, Brasil).

O Questionário de Frequência de Consumo Alimentar é considerado o método de avaliação da ingestão dietética, mais prático e informativo, uma vez que permite a obtenção de dados retrospectivos por períodos mais longos (FISBERG et al., 2005). Será aplicado para avaliar o consumo habitual de alimentos fontes de substâncias antioxidantes nos meses anteriores ao início dos protocolos de intervenção e durante o estudo. Esse questionário será aplicado e avaliado por nutricionista, utilizando-se o software *Avanutri* versão 4.0 (AVANUTRI- RJ, Brasil).

*6.5 Descrição das vídeo games utilizados*

*6.5.1 Descrição do vídeo game ativo:* o console será o *XboX* *360* com sensor *Kinect* (Foxconn®, Nova Taipé, China) e o jogo utilizado será o *Just Dance Summer Party* (Ubisoft®, Montreiul, França). Inicialmente, o sensor *Kinect* capta os sinais de movimento de todos os segmentos corporais do jogador. A partir daí, durante os jogos executados, será necessário que o jogador realize movimentos corporais considerados ativos para manipular o *avatar* gerado na tela. O nível de dificuldade do jogo adotado será o iniciante, visto que os participantes não terão experiência prévia com VGA. No caso do jogo *Just Dance Summer Party*, as movimentações corporais são semelhantes a passos de dança, com a utilização de diferentes músicas, como mostrado no anexo B.

*6.5.2 Descrição do vídeo game sedentário:* a sessão será semelhante as sessões de VGA, com o mesmo tempo de duração e as mesmas medidas de FC, PSE, acelerometria, PA e MAC. Nesta sessão será utilizado o *Dance Dance Revolution®* (SSD Company Ltd., Shiga, Japão) para *Playstation 2®* (San Mateo, CA, EUA), onde o praticante simulará uma dança sincronizada, entretanto utilizando o *joystick* tradicionalmente conhecido e as participantes permanecerão por toda a sessão em posição sentada. O estilo das músicas utilizadas no jogo é semelhante às usadas em academias de ginástica.

*6.6 Protocolos de adaptação*

*6.6.1 Adaptação ao jogo*: as participantes realizarão um período de adaptação ao jogo com duração de três sessões, onde a primeira sessão terá duração de 10 minutos e a última, de 40 minutos que será alcançado de maneira gradual. Durante as sessões, as participantes serão orientadas da maneira correta de como manusear e utilizar o jogo.

*6.6.2 Adaptação ao exercício em esteira:* nos mesmos dias em que ocorrerão as sessões de adaptação ao jogo, as participantes realizarão sessões de caminhada em esteira, sendo no primeiro dia, com duração de 30 minutos, no segundo dia com duração de 40 minutos e o terceiro dia com duração de 60 minutos. A intensidade adotada nestas sessões será livre, no entanto as participantes serão incentivadas a atingir a intensidade moderada (60% a 85% da FCM).

*6.7 Procedimentos para as sessões agudas:* em cada sessão, ao chegar ao local de coleta, as participantes permanecerão em repouso por um período de 20 minutos, em seguida serão realizadas as medidas de repouso de FC, PA e MAC, logo após elas serão instrumentadas com o acelerômetro, o aparelho portátil para consumo de oxigênio para mensuração do gasto energético/deslocamentos. Será iniciada a sessão escolhida de forma randomizada com duração de 60 minutos. A FC, consumo de oxigênio e acelerometria serão mensurados durante toda a sessão e PSE será questionada a cada 10 minutos. Imediatamente ao final da sessão será mensurada novamente a PA e MAC e se repetirão durante 60 minutos, a cada 10 minutos, com as participantes permanecendo em repouso. Durante este período será aplicada a *Enjoyment Scale.* Durante a sessão EIM, onde as participantes deverão atingir intensidade do exercício moderada, será prescrito o percentual de frequência cardíaca de 60 e 85% da FCM (intensidade moderada) e esta será realizada a partir dos dados obtidos no teste ergoespirométrico. Além disso, na EIJ, onde o percentual de frequência cardíaca adotado será semelhante ao atingido na sessão VGA, não será predeterminada a intensidade adotada. Nas sessões VGA e VGS não serão adotadas intensidades previamente estabelecidas.

*6.8 Protocolo de treinamento:* as participantes serão distribuídas de maneira randomizada em dois grupos (VGA e controle). O treinamento terá duração de 8 semanas, com frequência de 3x/semana, totalizando 24 sessões. As sessões semanais acontecerão em dias intercalados, considerando pelo menos 48h de intervalo entre elas e aos domingos não acontecerão sessões de treinamento. As sessões ocorrerão em domicílio, com a presença do pesquisador para sua realização. Inicialmente as participantes permanecerão em repouso por 10 minutos, logo em seguida serão mensuradas a PA e a FC de repouso. Será iniciada a sessão do treinamento com o VGA, onde serão mensurados o gasto energético/deslocamentos, a FC e a PSE durante o jogo que terá duração de 40 minutos. Imediatamente ao final da sessão de jogo, será novamente mensurada a PA e será aplicada a *Enjoyment Scale.* O grupo controle não realizará atividade alguma e terá visitas semanais para a mensuração de PA e MAC.

*6.9 Descrição das variáveis mensuradas*

*6.9.1 Medidas de frequência cardíaca e Percepção subjetiva de esforço:* os sujeitos serão instrumentados com o monitor cardiofrequêncímetro da marca Polar, modelo RS800cx (Polar ElectroOy, Kempele, Finland) e no início de cada sessão, permanecerão sentados por 10 minutos para que seja mensurada a FC de repouso. Esta variável será monitorada durante toda a sessão.

Durante as sessões de adaptação ao VGA e ao exercício em ergômetro a Escala de Percepção Subjetiva de Esforço (PSE) de Borg (NOBLE et al., 1983) será apresentada às participantes com índices de 6 a 20 para que as mesmas se familiarizem com os estágios de fadiga que vão de muito leve a exaustivo. A percepção subjetiva de esforço será mensurada a cada 10 minutos durante as sessões.

*6.9.2 Protocolo para medidas de pressão arterial em local de coleta:* as participantes serão solicitadas a permanecer 10 minutos em repouso e após este período será verificada a PA basal. Nas sessões do protocolo de intervenção aguda, novas medidas serão tomadas em repouso, imediatamente ao final da sessão de vídeo game ativo e a cada 10 minutos durante um período de recuperação de 60 minutos. Durante o protocolo de intervenção crônica, estas medidas serão realizadas na residência das participantes. Estas medidas serão realizadas pelo método auscultatório seguindo as V Diretrizes Brasileiras de Hipertensão Arterial (2010).

*6.9.3 Protocolo de Monitorização Ambulatorial da Pressão Arterial (MAPA):* a Monitoração Ambulatorial da Pressão Arterial (MAPA) será realizada na semana da visita inicial do estudo, bem como, ao final do protocolo de intervenção crônica. PA e FC serão monitorados por um período de 24 horas, usando um aparelho modelo Dyna-MAPA+ da marca *Cardios®* (São Paulo, Brasil). O monitor será programado para realizar as medidas a cada 15 minutos, durante o período de vigília, e a cada 30 minutos, durante o sono, de forma que ao final das 24 horas obtenha-se, ao menos, 16 medidas válidas no período da vigília e oito durante o sono, de acordo com as recomendações da V Diretrizes Brasileiras de Monitorização Ambulatorial da Pressão Arterial (MAPA V) e III Diretrizes Brasileiras de Monitorização Residencial da Pressão Arterial (MRPA III) (2011).

O protocolo de instalação do aparelho ocorrerá conforme o preconizado pelas Diretrizes Brasileiras MAPA V e MRPA III (2011). Inicialmente, será mensurada a circunferência do braço a fim de selecionar o manguito com largura e comprimento adequado. Uma medida clínica da PA será previamente realizada com o voluntário sentado após 10 minutos de repouso, em ambos os membros superiores, utilizando esfigmomanômetro de coluna de mercúrio, antes de instalar o aparelho. O manguito será colocado de 2 a 3 cm acima da fossa cubital, no braço não dominante. Após a colocação do equipamento será comparada a medida obtida pelo monitor de MAPA com a medida obtida previamente com o esfigmomanômetro de coluna de mercúrio, certificando-se de que as diferenças não sejam superiores a 5 mmHg. Será entregue para cada participante um diário de atividades para registro das atividades realizadas durante as 24 horas, além de conter todas as instruções para a realização do exame (anexo C).

*6.9.4 Protocolo de teste ergoespirométrico:* a avaliação da capacidade aeróbia máxima será realizada através da medida direta do consumo de oxigênio no pico do exercício (VO_2_ pico). O protocolo utilizado será o de rampa, onde será acrescida a inclinação da rampa a cada dois minutos de duração do teste. Simultaneamente ao teste de esforço, o indivíduo será conectado a um ergoespirômetro computadorizado (Vmax, USA®) através de um sistema de válvula e sensor onde a ventilação pulmonar (VE) será medida a cada expiração. Através de sensores de oxigênio (O_2_) e de dióxido de carbono (CO_2_) serão analisadas as concentrações de O_2_ e CO_2_, respectivamente a cada ciclo respiratório. A partir das análises da VE e das concentrações dos gases expirados, serão calculados o VO_2_ e a produção de CO_2_. Será considerado como VO_2_ de pico o consumo de O_2_ obtido no pico do exercício, quando o indivíduo não mais conseguir sustentar a intensidade da corrida na esteira. Além da determinação da capacidade funcional máxima do indivíduo, serão determinados o limiar anaeróbio (LA) e o ponto de descompensação respiratória (PDR) (SKINNER, 1980). O LA será considerado no minuto em que o indivíduo apresentar valores mais baixos do equivalente ventilatório O_2_ (VE/VO_2_) e da pressão parcial de O_2_ no final da expiração (PetO_2_), antes destes terem um aumento progressivo e de haver um incremento não linear do valor de razão de troca respiratória (RER). O ponto de compensação respiratório será considerado no minuto em que o indivíduo apresentar valores mais baixos de equivalente ventilatório de CO_2_ (VE/VCO_2_) antes de haver um aumento progressivo deste e do valor máximo de pressão parcial de CO_2_ no final da expiração (PetCO_2_) antes de começar a apresentar uma queda progressiva.

*6.9.5 Registro da Modulação Autonômica Cardíaca (MAC):* será avaliada por meio da variabilidade da frequência cardíaca, uma medida simples e não invasiva dos impulsos autonômicos originados no bulbo e direcionados para o coração, o que indica uma medida da atividade do sistema nervoso autônomo na regulação cardiovascular. Esta medida será feita por meio de avaliação das oscilações no intervalo entre batimentos cardíacos consecutivos (intervalos R-R) da frequência cardíaca (VANDERLEI et al., 2009).

A MAC será determinada por meio do registro da variabilidade dos intervalos R-R de frequência cardíaca, através de um monitor de frequência cardíaca da marca *Polar*, modelo RS800CX (PolarElectroOy, Kempele, Finland). Este instrumento foi validado perante registro com eletrocardiograma em repouso e durante o exercício (NUNAN et al., 2008; PORTO; JUNQUEIRA, 2009). Os sujeitos ficarão em repouso por 10 minutos, após os quais se iniciará o registro dos intervalos R-R. Este registro será feito, com os voluntários sentados, por um período mínimo de cinco minutos para que se obtenha um registro de pelo menos 300 batimentos. Os dados serão transferidos para um computador provido do software do mesmo fabricante e, em seguida, transferidos para o software *Kubios HRV*, versão 2.0 (University of Kuopio, Finlândia). Os dados serão analisados no domínio do tempo, sendo considerados a média e o desvio padrão dos intervalos individuais do R-R. No domínio da frequência, serão consideradas as bandas de baixa frequência (0,04 a 0,15 Hz) e alta frequência (0,15 a 0,4 Hz), como medidas de atividade elétrica proveniente de impulsos simpático e parassimpático respectivamente. Será adotada ainda a razão baixa frequência/ alta frequência como balanço autonômico.

*6.9.6 Acelerometria:* durante todas as sessões, será utilizado um acelerômetro da marca *Actigraph®* GT3SX (Pensacola, EUA) para avaliação da aceleração do corpo, calibrado de acordo com as especificações do fabricante. O intervalo de tempo adotado será em minutos e a saída de dados expressa em contagens médias por minuto. O aparelho será fixado na altura da cintura, ao lado direito, por uma cinta de elásticos e fivela ajustável ou na roupa das participantes. Ao final dos registros, os dados serão transferidos para um computador e será analisado através do software *SAS* 9.2 (SAS Institute Inc., Cary , NC 25513). Os valores de referêcia adotados serão: 0 a 99 contagens/min-1, será considerada atividade sedentária, 100 a 1951 contagens/min-1, atividade moderada e de 1952 a 5723 contagens/min-1, será considerada atividade vigorosa.

*6.9.7 Consumo de oxigênio através do analisador de gases pulmonares portátil K4b2:* este equipamento portátil possibilita a verificação, em cada respiração, dos parâmetros respiratórios, medindo o consumo de oxigênio e a produção de dióxido de carbono durante o exercício físico. O instrumento utilizado será o analisador de gases pulmonares portátil *K4 b2* (Cosmed Copyritgh©, Roma, Itália) validado no estudo realizado por McLaughlin et al. (2001) e será utilizado durante as sessões de intervenção aguda.

*6.9.8 Enjoyment Scale (avaliação do prazer/gozo):* esta escala é composta por 18 itens que iram avaliar o nível de prazer/gozo que a atividade realizada pode proporcionar, onde cada item possui sete níveis, o nível um corresponde a “Eu gosto muito” e o nível sete corresponde a “Eu odeio” (apêndice F). Esta escala foi validada para utilização na população adulta de acordo com Graves et al. (2010) e sua pontuação é calculada através da média dos valores obtidos em cada item e quanto maior a média, mais prazer/gozo a atividade realizada proporcionou.

*6.9.9 Protocolo de coleta sanguínea:* durante a visita inicial ao estudo bem como na quarta semana de intervenção crônica e ao final do estudo, uma enfermeira experiente coletará 10 mL de sangue venoso, retirados da veia antecubital de cada voluntário, após jejum de 12 horas. As amostras serão centrifugadas a 3000 rpm por 15 minutos e o sobrenadante (soro ou plasma) transferido para microtubos e refrigerado a -20°C ou 4°C até as análises.

*6.10 Dosagens bioquímicas*

*6.10.1 Perfil glicêmico e lipídico:* Análises do perfil glicêmico e lipídico serão realizadas em amostras de soro, através de kits comerciais da marca *Labtest* (Minas Gerais- Brasil), seguindo as recomendações do fabricante e em analisador automático *Labmax 240 premium* (Lagoa Santa-MG, Brasil). As concentrações de glicose sanguínea serão determinadas através do método enzimático colorimétrico da glicose oxidase proposto por Trinder (1969). A absorbância será obtida no comprimento de onda 505nm.

Colesterol total será determinado por método enzimático proposto por Trinder (1969) a 500nm. HDL-c será quantificado por método manual. Para este procedimento, um volume de 0,25 µL de substância precipitante será adicionado a 0,25 µL de amostra em microtubos e misturados vigorosamente por 30 segundos. Em seguida, centrifugou-se a 3.500 rpm por 15 minutos, sendo o sobrenadante retirado e colocado em alíquotas contendo 1 µL do reagente 1 do *kit Colesterol Liquiform*, e posto no banho-maria por 10 minutos. Por fim, será feita a leitura em espectrofotômetro ultravioleta (Biospectro, modelo SP-220/Brasil), a 500 nm.

Os valores de triglicérides serão determinados através do método enzimático proposto por Trinder (1969), e a absorbância será obtida no comprimento de onda 505nm. Os valores das lipoproteínas de baixa densidade (LDL-c) e lipoproteínas de muito baixa densidade (VLDL-c) serão estimados pela equação de Friedewald [LDL-C= (CT – HDL-C) – (TG /5)](FRIEDEWALD, LEVY e FREDRICKSON, 1972).

Os valores de referência para as variáveis do perfil lipídico e glicêmico de adultos encontram-se dispostos na tabela 1.

Tabela 1 – Valores de referência para as variáveis do perfil lipídico e glicêmico.

|  | Baixo | Desejável | Limítrofe | Elevado | Muito elevado |
| --- | --- | --- | --- | --- | --- |
| **Glicose**  **(mg/dL)** |  | 70-99 |  |  |  |
| **CT**  **(mg/dL)** |  | < 200 | 200-239 | ≥ 240 |  |
| **HDL-c**  **(mg/dL)** | < 40 (Homens)  < 50 (Mulheres) | ≥ 60 | 40-59 (Homens)  50-59 (Mulheres) |  |  |
| **LDL-c**  **(mg/dL)** |  | < 100 | 130-159 | 160-189 | ≥190 |
| **TG**  **(mg/dL)** |  | < 150 | 150-199 | 200-499 | ≥500 |

Fonte: American Association of Clinical Endocrinologists (2012)

*6.10.2 Análise da hemoglobina glicada (HbA1):* sua concentração será quantificada em amostras de plasma que poderão ser armazenados por até 8 dias. A quantificação será determinada por meio do kit comercial da marca *Labtest* (Minas Gerais, Brasil) conforme instruções do fabricante através do método de microcromatografia. Os valores entre 4% e 6% estão dentro da faixa normal de concentração de HbA1. Valores e torno de 7% é o valor adequado para pessoas acometidas por Diabetes. Valores acima de 8% estão em faixa de risco para complicações crônicas.

*6.10.3 Análise de Proteína c-reativa ultrassensível (PCR-us):* a concentração de PCR-us será quantificada por imunoturbidimetria em amostras de soro. As concentrações de PCR-us serão determinadas por meio do kit comercial da marca *Labtest* (Minas Gerais, Brasil) conforme instruções do fabricante. Para calibração será utilizado o calibrador da série Calibra da *Labtest* (Calibra Plus PCR-ultra – Ref-345). A absorbância será obtida no analisador automático *Labmax 240 premium* (Lagoa Santa-MG, Brasil), no comprimento de onda 540nm. Os valores séricos de referência da PCR-us serão baseados nos pontos de corte para risco de eventos cardiovasculares na população adulta: < 1,0 mg/L (baixo risco), 1,0-3,0mg/L (médio risco) e > 3,0 mg/L (alto risco) de acordo com Pearson et al. (2003).

*6.10.4 Análise de Alfa-1-glicoproteína ácida (A1GPA):* A concentração de A1GPA será quantificada por imunoturbidimetria em amostras de soro, por meio do kit comercial (Labtest, Minas Gerais, Brasil) conforme instruções do fabricante. Para calibração será utilizado o calibrador da série Calibra da *Labtest* (Calibra Plus Proteína – Ref-346). A absorbância será obtida no analisador automático *Labmax 240 premium* (Lagoa Santa-MG, Brasil), no comprimento de onda 340nm.

*6.10.5 Atividade Antioxidante pelo DPPH (Radical 2,2-difenil-1-picril-hidrazila): a* quantificação dos níveis de antioxidantes no soro será realizada de acordo com a metodologia descrita por Chrzczanowicz et al. (2008). Ocorrerá por meio de um ensaio espectrofotométrico utilizando-se uma solução de 2,2 difenil-1-picril-hidrazila (DPPH) 0,004% em metanol (MeOH), misturada à solução da amostra em análise em diferentes concentrações. Após 30 minutos de reação, as absorbâncias das soluções serão determinadas em 540 nm e em espectrofotômetro (Bioespectro, modelo SP 22, Brasil). A molécula radicalar DPPH apresenta absorção máxima a 540 nm e coloração violeta que se transforma em amarela quando se reduz. Essa forma reduzida corresponde à molécula do radical livre DPPH pareado com um hidrogênio do antioxidante (DPPH-H). A descoloração resultante é estequiométrica, com o número de moléculas radicalares sequestradas (MOLYNEUX, 2004).

A desproteinização do soro será otimizada, realizando-se testes com acetonitrila (CH3 CN) 9,5 M em H2O, CH3CN 100% e metanol (CH3OH) 100%. Ao final, será adicionado 200mL de CH3CN em 200 mL de soro e a mistura incubada por dois minutos à temperatura ambiente e centrifugada por dez minutos em 11.000 rpm  à 4ºC, sendo retirado 25 mL do sobrenadante, que corresponde ao soro desproteinizado. A esta fração será adicionada 970 mL de CH3OH e 5 mL de solução de DPPH. A mistura será agitada em vórtex, colocada em repouso à temperatura ambiente por 20 minutos, centrifugada por dez minutos e, então,submetida a 11.000 rpm a 4ºC. 200 mL do sobrenadante de cada amostra transferida para microplaca de 96 poços, sendo realizada leitura em 540 nm. A solução de referência (branco) será constituída por 25µL de H2O em substituição ao volume do soro sanguíneo (amostra).

*6.10.6 Análise da atividade antioxidante (SOD):* a atividade antioxidante será determinada por meio da atividade da enzima superóxido dismutase (SOD), de acordo com Sun et al. (1988). Esta será avaliada através da sua capacidade em inibir a redução fotoquímica do azul de nitro-tetrazolio (NBT). Os resultados serão calculados como a quantidade de SOD necessária para inibir a taxa de redução do NBT em 50%. As amostras serão centrifugadas por 10 minutos a 3600 rpm a 4°C. O sobrenadante será retirado e centrifugado novamente por 20 min a 12000 rpm a 4°C. Em uma câmara escura serão misturados 1mL do meio de reação (tampão fosfato 50mM, EDTA 100nM e L-metionina 13mM pH 7,8) com 30 μL da amostra, 150μL do NBT 75μM e 300 μL riboflavina 2μM. Os tubos contendo a solução obtida serão expostos a lâmpadas fluorescentes (15W) por 15 minutos. Ao final, o material será lido em espectrofotômetro a um comprimento de onda de 560nm.

*6.10.7 Análise de Malondialdeído (MDA):* a atividade oxidante será quantificada por meio da reação do ácido tiobarbitúrico (TBARS) com os produtos de decomposição dos hidroperóxidos. Para isto, 250 µl de amostra será incubada em banho-maria a 37° C por 60 minutos. Em seguida, a amostra será precipitada com ácido perclórico AA 35% e centrifugada a 14000 rpm por 20 minutos à 4°C . O sobrenadante será transferido para novos microtubos onde será adicionado 400µl de ácido tiobarbitúrico a 0,6% e incubado a 60° C por 60 minutos. Após o resfriamento, o material será lido em espectrofotômetro da marca Biospectro (SP-220/Brasil) a um comprimento de onda de 532nm.

*6.10.8 Análise de Oxido Nítrico (NO) pela quantificação de nitrito plasmático:* A produção endógena de NO será determinada em função da concentração plasmática do seu metabólito nitrito. A concentração de nitrito foi determinada pela reação de Griess que quantifica o nitrito na amostra através da reação de diazotizerção formando um cromóforo de cor rósea. O reagente foi preparado utilizando partes iguais de ácido fosfórico 5%, sulfanilamida 1% em ácido fosfórico a 5%, N-(1-Naphtyl)-ethylenediaminedihydrochloride (NEED) a 0,1% e água destilada. Segue-se a detecção do nitrito/nitrato com a adição de 500 µL do reagente de Griess a 500 µL do plasma. Após 10 minutos, a absorbância será medida em um espectrofotômetro (Biospectro, SP-220/Brasil) a um comprimento de onda de 532nm. As concentrações de nitrito serão calculadas por extrapolação para uma curva padrão de NaNO2 e os dados expressos em micromoles (GREEN, TANNERNBAUM e GOLDMAN, 1981).

Todas as análises bioquímicas serão realizadas no Laboratório de Estudos em Treinamento Físico Aplicado ao Desempenho à Saúde (Departamento de Educação Física – Universidade Federal da Paraína), sendo as variáveis bioquímicas perfil glicêmico e lipídico e inflamação sistêmica realizada no analisador automático LABMAX 240 Premiun (Labteste Diasgnóstica S/A, Minas Gerais, Brasil) variáveis bioquímicas relacionadas ao estresse oxidativo serão realizadas no mesmo local, no entanto utilizando o método manual de análises.

*6.11 Análise estatística:* aos dados serão aplicados os testes de Shapiro-Wilk para verificar a normalidade e Levene para verificar a homogeneidade. Caso os dados sejam distribuídos de maneira normal, os resultados serão apresentados como média e erro padrão da média. O teste estatístico utilizado para comparação entre os valores de repouso, bem como comparações entre mensurações durante as diferentes sessões do protocolo de intervenção aguda será o ANOVA *one way*. Para comparações entre as mensurações durante o período de recuperação das sessões do protocolo de intervenção aguda, bem como para comparações entre os momentos pré, durante e após as sessões do protocolo de intervenção crônica, será utilizado o ANOVA two way. Para comparações entre os dois diferentes grupos da intervenção crônica, uma vez comparados os momentos pré – pré e pós – pós, será utilizado o Teste t de *Student* independente e para comparação pré - pós de cada um dos grupos do protocolo de intervenção crônica, será utilizado o Test t de *Student* dependente. Os dados serão analisados por meio do *software* *Instat 3.0* (GraphPad, San Diego, CA, USA), adotando significância de p<0,05.

1. **REFERÊNCIAS**

ALVES, L. L; FORJAZ, C. L. Influência da intensidade e do volume do treinamento aeróbico na redução da pressão arterial de hipertensos. **Revista Brasileira de Ciências do Movimento,** v.15, p.115-122, 2007.

ANUNCIAÇÃO, P. G; POLITO, M. D. A review on post-exercise hypotension in hypertensives individuals. **Arquivos Brasileiros de Cardiologia**, v.5, n.96, p.100-109, 2011.

ARAÚJO, C. G. Fisiologia do exercício físico e hipertensão arterial: uma breve discussão. **Revista Hipertensão,** v.4, p. 78-83, 2001.

BACCHI, E. et al. Metabolic effects of aeróbic training and resistance training in type 2 diabetic subjects. **Diabetes Care,** v.35, abr. 2012.

BALDI, J.C; SNOWLING, N. Resistance training improves glycaemic control in obese type 2 diabetic men. **Int J Sports** **Med**, v.24, p. 419–423, 2003.

BASSUK, S. S; MANSON, J. E. Epidemiological evidence for the role of physical activity in reducing risk of type 2 diabetes and cardiovascular disease. **J**.**Appl**. **Physiol**. n. 99, p. 1193-1204, 2005.

BENEDETTI, T. R. B. et al. Reprodutibilidade e validade do Questionário Iternacional de Atividade Física (IPAQ) em homens idosos. **Revista brasileira de Medicina do Esporte,** v.13, n.1, jan/fev. 2007.

BERMUDES, A. M. L. M.. et al. Monitoração ambulatorial da pressão arterial em indivíduos normotensos submetidos a duas sessões univer de exercícios: resistido e aeróbio. **Arquivos Brasileiros de Cardiologia,** v.82, n.1, p.57-64, 2003.

Boulé NG, Haddad E, Kenny GP, Wells GA, Sigal RJ Effects of exercise on glycemic control and body mass in type 2 diabetes mellitus: a meta-analysis of controlled clinical trials. **JAMA**, v.286, p. 1218-1227, 2001.

BRITO, A. F. et al. High-intensity exercise promotes postexercise hypotension greater tha moderate intensity in elderly hypertive individuals. **Clinical Physiology and Functional Imaging,** v.2, n.34, p.126-132, 2014.

BROEREN, Virtual rehabitation in an activity centre for community-dwelling persons with stroke. 9:24 he possibilities of 3-dimensional computer games. **Cerebrovascular Diseases,** v.3, n.26, p.289-296, 2008.

CASONATTO, J; POLITO, M. D. Hipotensão pós-exercício aeróbio: uma revisão sistemática. **Revista Brasileira de Medicina do Esporte,** v.15, n.2, p.151-157, mar/abr, 2009.

CASTANEDA, C. et al. A randomized controlled trial of resistance exercise training to improve glycemic control in older adults with type 2 diabetes. **Diabetes Care**, v.25, p.2335-2341, 2002.

COLBERG, S. R. et al. Exercise and type 2 diabetes: the American College of Sports Medicine and the American Diabetes Association: joint position statement. **Diabetes Care,** v.33, p. 147-167, 2010.

CORNELISSEN, V. A; FAGARD, R. H. Effect of resistance training on resting blood pressure: a meta-analysis of randomized controlled trials. **Journal of Hypertension,** v.23, n.2, p.251-259, 2005.

DALL, C. H. et al. Effect of high-intensity training versus moderate trainig on peak oxygen uptake and chronotropic response in hearth transplant recipientes: a randomized crossover trial. **American Journal of Transplantation,** aug, 2014.

DELA, F. et al. Insulin-stimulated muscle glucose clearance in patients with NIDDM. Effects of one-legged physical training. **Diabetes**, v. 44, p.1010-1020, 1995.

DORNAS, W. C; OLIVEIRA, T. T; NAGEM, T. J. Exercício físico e diabetes mellitus tipo 2. **Arquivos de Ciências da Saúde UNIPAR,** v.15, n.1, p.95-107, jan./abr. 2011.

DUCLOS, M; VIRALLY, M. L; DEJAGER, S. “Exercise in the management of type 2 diabetes mellitus: what are the benefits and how does it work?” **Physician and Sportsmedicine,** v.39, n.2, p.98–106, 2011.

FEX, A. et al. Effect of elliptical high intensity interval training on metabolic risk fator in pre-and type 2 diabetes patients: a pilot study. **Journal of Physical Activity & Heath,** aug. 2014.

FISBERG, R. M. et al. **Inquéritos alimentares**: **métodos e bases científicos**. Barueri, São Paulo: Manole, 2005, p.334.

FISBERG, R. M. et al. **Inquéritos alimentares**: **métodos e bases científicos**. Barueri, São Paulo: Manole, 2005, p.334.

FLORINDO, A. A. et al. Epidemiology of leisure, transportation, occupational, and household physical activity: prevalence and associated factors, **Journal od physical activity & Health**, v.6, p.625-632, 2009.

FLORINDO, A.A. et al. Epidemiology of leisure, transportation, occupational, and household physical activity: prevalence and associated factors. **J Phys Act Health,** v. 6, p. 625-632, 2009.

FORJAZ, C. L. M. et al. A duração do exercício determina a magnitude e a duração da hipotensão pós-exercício. **Arquivos Brasileiros de Cardiologia,** v.70, n.2, p.99-104, 1998.

FORJAZ, C. L. M. et al. Postexercise hypotension and hemodynamics: the role of exercise intesity. **The Journal os Sports Mecidine and Physical Fitness,** v.44, p.54-62, 2004.

FRIEDEWALD, W. T.; LEVY, R. I.; FREDRICKSON, D. S. Estimation of the concentration of low-density lipoprotein cholesterol in plasma, without use of the preparative ultracentrifuge. **Clinical Chemistry**, v. 18, n. 6, p. 499-502, 1972.

GARCIA, A. N. et al. Influência de um sistema de vídeo game (Nitendo Wii) no tratamento fisioterápico de pacientes pós-acidente vascular encefálico. **Brazilian Journal od Physical Therapy,** v.14, n.2, p.48, 2010.

GIBSON, R. S. Food consumption of individuals. In: **Principles of nutritional assessement**. New York: Oxford University Press, 1990.

GILLEN, J. B, et al. Acute high-intensity interval exercise reduces the postprandial glucose response and prevalence of hyperglycaemia in patients with type 2 diabetes. **Diabetes Obes Metab,** v.14, p.575-577, 2012.

GILLEN, J.B. et al. Acute high-intensity interval exercise reduces the postprandial glucose response and prevalence of hyperglycaemia in patients with type 2 diabetes. **Diabetes, Obesity and Metabolism,** v.6, n.14, p.575–577, 2012.

GRAVES, L. E. F. et al. The physiological cost and enjoyment of Wii Fit in adolescentes, Young adults, and older adults. **Journal of Physical and Hearth,** v.7, n.3, p. 393-401, 2010.

GRAVES, L. E. F; RIDGERS, N. D; STRATTON, G. The contribution of upper limb and total body movement to adolescents’energy expenditure whilst playing Nintendo Wii. **European Journal of Applied Physiology,** v. 4, n.104, p.617-623, 2008.

GREEN, L. C; RUIZ DE LUZURIAGA, K; WAGNER, D. A; RAND, W; ISTFAN, N; YOUNG, V. R; TANNENBAUM, S. R.Nitrate biosynthesis in man.**Proceedings of the National Academy of Sciences of the United States of America,**v.12, n.78, p.7764–7768, 1981.

GUIDRY, M. A. et al. The influence os short and long duration on the blood pressure response to na acute bout of dynamic exercise. **American Heart Journal,** v. 151, n.6, p. 1322.e6-1322.e12, 2006.

HAGBERG, J. M; PARK, J. J; BROWN, M. D. The role of exercise training in the treatment of hypertension: na update. **Sports of Medicine,** v.30, p.193-206, 2000.

HALBERT, J. A. et al. The effectivess of exercise training in lowering blood pressure: a meta-analysis of randomized controlled trials of 4 weeks or longer. **Journal of Human Hypetension,** v.11, p.641-649, 1997.

HALLIWILL, J. R; TAYLOR, A; ECKBERG, D. L. Impaired sympathetic vascular regulation in humans after acute dynamic exercise. **Journal of Physiology,** v.1, n.495, p.279-288, 1996.

HAMER, M. The anti-hypertensive effects of exercise. **Sports of Medicine,** v.2, n.36, p.109-116, 2006.

HANGINS, M. et al. Effectiveness of yoga for hypertension:systematic review and meta-analysis. **Evidence-Based Complementary and Alternative Medicine,** p.1-13, 2013.

HASKELL, W. L; LEE, J; PATE, R. R. Physical activity and public health: updared recommendation for adults from American College os Sports Medicine and the American Heart Association. **Medicine & Science in Sports & Exercise,** p. 1423-1434, 2007.

HENRIQUES, M. Atividade física para a saúde: recomendações. **Revista Factores de Risco,** n.29, p.36-44, abr-jun 2013.

HOLMES, H. et al. Xbox Kinect™ representes high intesity exercise for adults with cystic fibrosis. **Journal of Cystic Fibrosis,** p. 1-5, 2013.

HUNG, J. et al. Effect of 12 week tai chi chuan exercise on peripheral nerve modulation in patients with type 2 diabetes mellitus. **Journal of Rehabilitation Medicine,** v.41, p.924-929, 2009.

JENG, C. et al. Effects of arm exercise on sérum glucose response in type 2 DM patients. **Journal of Nursing Research,** v.10, n.3, p.187, 2002.

JONES, H. et al. Is the magnitude of acute post-exercise hypotension mediated by exercise intensity or total work done? **Eur J Appl Physiol,** v.102, p.102-133, 2007

KENNEY, M. J; SEALS, D. R. Posexercise hypotension. **Hypertension,** v.22, n.5, p.653-664, 1993.

KEVIN, C. et al. The effects of sigle bouts of aerobic exercise, exergamin, and videogame play on cognitive control. **Clinical Neurophysiology,** v. 122, p.1518-1525, 2011.

LAMARCHE, B. et al. Is body fat loss a determinant factor in the improvement of carbohydrate and lipid metabolism following aerobic exercise training in obese women? **Metabolism.** V.41, p.1249-1256, 1992.

LANNINGHAM-FOSTER, L; JENSEN, T.B; FOSTER, R.C; REDMOND, A.B; WALKER, B.A; HEINZ,D; LEVINE, J.A. Energy expenditure of sedentary screen time compared with active screen time for children. **Pediatrics,** v. 118, n. 6, p.1831-1835, 2006.

LATERZA, M. C; RONDON, M. U. P. B; NEGRÃO, C. E. Efeito anti-hipertensivo do exercício. **Revista Brasileira de Hipertensão,** v.2, n.14, p.104-111, 2007.

LEELAYUWAT, N. **Beneficial effects of alternative exercise in patients with diabetes type II.** June, 2013.

LIU, S. et al. Blood pressure responses to acute and chronic exercise are related in prehypertension. **Medicine & Science in Sports & Exercise,** p. 1644-1652, 2012.

MACDONALD, J. R. et al. Post exercise hypotension is not mediated by the serotonergic system in borderline hypertensive individuals. **J Hum Hypertens**, v.16, p.33-39, 2002

MACDONALD, J. R. Potential Causes, Mechanisms, and impication of post exercise hypotension. **Journal of Human Hypertension,** v.16, p.225-236, 2002.

MADDISON, R. et al. A.energy expended playing video console games: an opportunity to increase children's physical activity? **Pediatric Exercise Science,** v.3, n.19, p. 334-343, 2006.

MADSEN, K. A. et al. Feasibility of a Dance Videogame to promote weight loss among overweight children and adolescents. **Archives of Pediatrics and Adolescents Medicine,** v.161, p. 105-107, 2007.

MANJERI, F. et al. A stanrd ballroom and latin dance program to improve fitness and adherence to physical activity in individuals with type 2 diabetes and in obesity. **Diabetology & Metabolic Syndrome,** v. 74, n.6, p.1-8, 2014.

MARWICK, T. H. et al. Exercise Training for Type 2 Diabetes Mellitus: Impact on Cardiovascular Risk:A Scientific Statement From the American Heart Association. **Circulation**, v.25, n.119, p. 3244-3262, 2009.

MATSUDO, S. Questionário internacional de atividade física (IPAQ): Estudo de validade e reprodutibilidade no Brasil. **Revista Brasileira de Atividade Física & Saúde,** v.6, n.2, 2001.

MELLECKER, R. R; MCMANUS, A. M. Active vídeo games and physical activity recommendations: a comparison of the gamercize stepper, XBOX Kinect and XaviX J-Mat. **Journal os Science and Medicine in Sport,** p. 1-5, 2013.

MERCURI, N; ARRECHEA, V. Atividade física e diabetes mellitus. **Diabetes Clínica,** v.4, p.347-349, 2001.

MILLS, A. et al. The effect of exergaming on vascular function in children. **The Journal of Pediatrics,** v. 163, n.3, p.806-810, 2013.

MINISTÉRIO DA SAÚDE. **VIGITEL Brasil 2011: vigilância de fatores de risco para doenças crônicas por inquérito telefônico**. Brasília: Ministério da Saúde; 2012.

MONTEIRO, L. Z. et al. Redução da pressão arterial, do IMC e da glicemia após treinamento aeróbio em idosas com diabete tipo 2. **Arquivos Brasileiros de Cardiologia,** v.5, n.95, p.563-570, 2010.

MORAIS, P. K. et al. Acute resistance exercise is more effective than aerobic exercise for 24 h blood pressure control in type 2 diabetics. **Diabetes & Metabolism**, Paris, v. 37, p. 112–117, 2011.

MUNIZ, T. S; MANUCHAQUIAN, L. M; ANDRADE, T. S. Hipotensão pós exercício de caráter aeróbio submetidos a indivíduos medicados, nos períodos matutino e vespertino. **Revista Brasileira de Prescrição e Físiologia do exercício,** v.4, n. 19, p.24-35, jan/fev. 2010.

NASCIMENTO, D. C. et al. Sutained effect of resistance training on blood pressure and hand grip strengh following a detraing period in elderly hypertensive women: a pilot study. **Clinical Interventions in Aging,** v.9, n.4, p. 219-225, 2014.

NI MHURCHU, C. et al. Couch potatoes to jumping beans: a pilot study of the effect of active video games on physical activity in children. **Int J Behav Nutr Phys Act,** v.5, n.8, 2008.

NOBLE, B. J. et al. A category-ratio perceived exertion scale: relationship to blood and muscle lactates and heart rate. **Medicine & Science in Sports & Exercise**, v. 15, p.523– 528, 1983.

NOBREGA, Thereza Karolina Sarmento da et al. Caminhada/corrida ou uma partida de futebol recreacional apresentam efetividade semelhante na indução de hipotensão pós-exercício. **Rev Bras Med Esporte,** São Paulo , v. 19, n. 1, fev. 2013.

NUNAN, D. et al. Levels of agreement for RR intervals and short-term heart rate variability obtained from the Polar S810 and an alternative system. **European Journal of Applied Physiology**, v. 103, n. 5, p. 529-37, 2008.

O’DONOVAN, C. O. et al. Energy expended playing Xbox Kinect™ and Wii™ games: a preliminar study comparing sigle and multiplayer modes. **Physiotherapy,** v.98, p.224-229, 2012.

O’DONOVAN, C. O; HUSSEY, J. Active vídeo games as a form o exercise and the effect of gaming experience: a preliminar study in health Young adults. **Physiotherapy,** v.98, p. 205-210, 2012.

O’HAGAN, C; DE VITO, G; BOREHAM, C. A. Exercise prescription in the treatment of type 2 diabetes mellitus: current practices, existing guidelines and future directions. **Sports Medicine,** v.1, n.43, p.39-49, jan. 2013.

OBERLIN, D. J. et al. One Bout of Exercise Alters Free-Living Postprandial Glycemia in Type 2 Diabetes. **Medicine & Science in Sports & Exercise,** v.2, n.46, p.232-238, 2014.

ORGANIZAÇÃO MUNDIAL DA SAÚDE- OMS. **Physical status:** the use and interpretation of anthropometry. Genebra, 1995.

OTTEN, J.J.; HELLWIG, J.P.; MEYERS, L.D. **Dietary Reference Intake: the essential gide to nutrient. Requirements**. Washington: The National Academies Press, 2006.

53

PENG, W. et al. Using active vídeo game for physical activity promotion: a systematic review of the current state of researsh. **Health Education & Behavior,** v.2, n.40, p.171-192, 2012.

PEREIRA, J. C. P. et al. Exergames como alternativa para o aumento do dispêndio energético: uma revisão sistemática. **Revista Brasileira de Atividade Física e Saúde,** v.5, n.17, p.332-340, 2012.

PESCATELLO, L. S. et al. Short term effect of dynamic exercise on arterial blood pressure. **Circulation,** v.83, p. 1557-1561, 1991.

PESCATELLO. L.S. et al. Exercise and hypertension. American College of Sports Medicine Position Stand. **Med Sci Sports Exerc***,* v.3, n.36, p. 533-553, 2004.

PIEPOLI, M. et al. Load dependence of changes in forearm and peripheral vascular resistance after acute leg exercise in man. **Journal of Physiology,** v.2, n.478, p.357-362, 1994.

PERRIER-MELLO, R. J. Video games ativos, equilíbrio e gasto energético em idoso: uma revisão sistemática. **ConScientiae Saúde,** v.2, n.13, p.289-297, 2014.

PITANGA, F. J. G; LESSA, I. Prevalência e fatores associados ao sedentarismo no lazer em adultos. **Cadernos de Saúde Pública*,*** Rio Janeiro, v. 21, n. 3, p.870-877, mai/jun. 2005.

PRAET, S. et al. Influence of Acute Exercise on Hyperglycemia in Insulin-Treated Type 2 Diabetes. **Medicine & Scince in Sports & Exercise,** v.38, p.2037–2044, 2006.

QUEIROZ, A. C. C. et al. Prescrição de caminhada não supervisionada, risco cardiovascular e aptidão física. **Revista Brasileira de Educação Física e Esporte,** v.3, n.27, p.377-386, jul/set. 2013.

RAUBER, S. B. Variáveis cardiovasculares durante e após a prática do VÍDEO GAME ativo “*Dance Dance Revolution”* e televisão. **Motriz,** v.19, n.2, p.358-367, abr/jun, 2013.

REZK, C. C. et al. Post-resistance exercise hypotension, hemodynamics, and heart rate variability: influence of exercise intensity. **European Journal of Applied Physiology,** v.98, p.105-112, 2006.

SAHAY, B. K. Role of yoga in diabetes. **JAPI,** v.55, p.121-126, feb. 2007.

SELL, K; LILLIE, T; TAYLOR. Energy expenditure during physically interactive vídeo game playing in male college students with diferente playing experience.. **Journal of American College Health,** v.5, n.56, p.505-512, 2013.

SIEGEL, S. R. et al. Active video/arcade games (exergaming) and energy expenditure in college students.**International Journal of Exercisescience,** v. 3,n.2, p.165-174, 2009.

SIGAL, R. J. et al. Effects of aerobic training, resistence training, or both on glycemin control in type 2 diabetes. **Annals of Interbal Medicine,** v.147, n.6, p.357-369, sep, 2007.

SIGAL, R.. J. et al. Physical activity/exercise and type 2 diabetes: a consensus statement from the American Diabetes Association**. Diabetes Care,** v.29, p.1433-1438, 2006.

SILVA, C. A; LIMA, W. C. Efeito benéfico do exercício físico no controle metabólico do diabetes mellitus tipo 2 à curto prazo. **Arquivos Brasileiros de Endocrinologia e Metabologia,** v.46, n.5, out. 2002.

SOCIEDADE BRASILEIRA DE CARDIOLOGIA, SOCIEDADE BRASILEIRA DE HIPERTENSÃO, SOCIEDADE BRASILEIRA DE NEFROLOGIA. V Diretrizes brasileiras de hipertensão arterial. Hipertensão. 2006; 9 (4): 121-56.

SOCIEDADE BRASILEIRA DE CARDIOLOGIA; SOCIEDADE BRASILEIRA DE HIPERTENSÃO; SOCIEDADE BRASILEIRA DE NEFROLOGIA. VI DIretrizes Brasileiras de Hipertensão. **Revista Hipertensão,** v.3, n.13, p.139-202, 2010.

SOCIEDADE BRASILEIRA DE DIABETES. **Diretrizes da Sociedade Brasileira de Diabetes.** Rio de Janeiro, 2013-2014.

STAIANO, A. E; CALVERT, S. L. The promise of exergames as tools to measure physical health. **Entretainment Computing,** v.2, p.17-21, 2011.

STRATTON, I. M. et al. Additive effects of glycaemia and bloos pressure exposure on risk of complications in type 2 diabetes: aprospective observation study (UKPDS 75). **Diabetologia,** v.8, n.49, p.1761-1769, may. 2006.

STRATTON, I. M. et al. Association of glycaemia with macrovascular and microvascular complications of type 2 diabetes (UKPDS 35): prospective observational study. **BMJ,** v.321, p. 405-412, 2000.

Tavares, L. F. et al. Síndrome metabólica em crianças e adolescentes brasileiros: revisão sistemática. **Caderno de Saúde Coletiva,** v. 4, n.18, p..469-476, 2010.

TREMBLAY, M. S. et al. Canadian sedentary behaviour guidelines for children and youth/Directives canadiennes en matiere de comportement sedentaire a l'intention des enfants et des jeunes.(Report). **Applied Physiology, Nutrition, and Metabolism***,*v.1, n.36, 2011.

TRINDER P. Determination of glucose in blood using glucose oxidase with an alternative oxygen receptor. **Annals of Clinical Biochemistry**, v. 6, p.24-27, 1969.

TSAI, J. et al. The beneficial effects of Tai Chi Chuan on blood pressure and lipid profile and anxiety status in radomized controlled trial. **The Journal Of Alternative and Complementary Medicine,** v.9, n.5, p.747-754, 2003.

TYAGI, A; COHEN, M. Yoga and hypertension: a systematic review. **Alternative Therapies in Health and Medicine,** v.2, n.20, p.32-59, 2014.

[UNNITHAN, V.B](http://www.ncbi.nlm.nih.gov/pubmed?term=Unnithan%20VB%5BAuthor%5D&cauthor=true&cauthor_uid=17006803); [HOUSER, W](http://www.ncbi.nlm.nih.gov/pubmed?term=Houser%20W%5BAuthor%5D&cauthor=true&cauthor_uid=17006803);[FERNHALL, B](http://www.ncbi.nlm.nih.gov/pubmed?term=Fernhall%20B%5BAuthor%5D&cauthor=true&cauthor_uid=17006803). Evaluation of the energy cost of playing a dance simulation video game in overweight and non-overweight children and adolescents*.***International Journal of Sports Medicine,** v.10, n.27, p.804-809, 2006.

VAN DIJK, J. W. et al. Both resistance- and endurance-type exercise reduce the prevalence of hyperglycaemia in individuals with impaired glucose tolerance and in insulintreated and non-insulin-treated type 2 diabetic patients. **Diabetologia,** v.55, p.1273-1282, 2012.

VAN DIJK, J. W. et al. Exercise and 24-h glycemic control: equal effects for all type 2 diabetes patients? **Medicine & Science in Sports & Exercise,** v.45, p.628-635,2013.

VANDERLEI, L. C. M. et al. Noções básicas de variabilidade da frequência cardíaca e sua aplicabilidade clínica. **Revista Brasileira de Cirurgia Cardiovascular**, v.24, n.2, p.205-217, 2009.

WHELTON, S. P. et al. Review: aerobic exercise reduces systolic and diastolic blood pressure in adults. **Annals of Internal Medicine,** v.136, n.2, p.493-503, 2002.

WILLIAMS, B. The year in hypertension, **JACC**, v.1, n.55, p.66-73, 2010.

WOLFF, M. et al. Impacto f yoga on blood pressure and quality of life in patients with hypertension – a controlled trial in primary care, matched for systolic blood pressure. **BMC Cardiovascular Dissorders,** v.13, n.111, p.2-9, 2013.

**APÊNDICES**

**APÊNDICE A –** Termo de consentimento livre e esclarecido


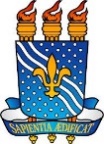

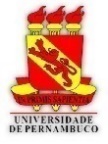


Prezado (a) Senhor (a)

Esta pesquisa é sobre o **EFICÁCIA DO VÍDEO GAME ATIVO NA PRESSÃO ARTERIAL E GLICEMIA EM HIPERTENSAS DIABÉTICAS: EFEITOS AGUDOS E CRÔNICOS** e está sendo desenvolvida por TAÍS FEITOSA DA SILVA*,* aluna do Curso de Pós-Graduação em Educação Física UFPB/UPE, sob a orientação do Prof. Dr. ALEXANDRE SÉRGIO SILVA. O objetivo do estudo é verificar a eficácia de um programa de treinamento com vídeo game ativo (VGA) (vídeo game que para jogar, é necessário realizar movimentos corporais considerados fisicamente ativos) sobre a pressão arterial e a glicemia. Juntamente com a pressão arterial e a glicemia, o seu colesterol, triglicerídeos, marcadores do estresse oxidativo e inflamação, peso e gordura corporal e também parte do seu sistema nervoso que controla o seu coração podem ser modificados pela pratica do VGA.

Para este estudo serão coletados dados sobre o seu consumo alimentar, medidas de peso corporal, altura, circunferência da cintura e pressão arterial. Será medido ainda como o seu sistema nervoso está controlando o seu coração através de um teste chamado de atividade autonômica cardíaca. Para isso, você ficará sentado (a) e uma cinta será colocada abaixo do peito e ligada a um relógio diferenciado. Será realizada coleta sanguínea para análises do seu colesterol, triglicerídeos, glicose, estresse oxidativo e inflamação. Esta coleta será realizada por uma enfermeira devidamente qualificada.

A realização do VGA será inicialmente apenas uma sessão e será avaliada agudamente e será realizada no Laboratório de Estudos em Treinamento Físico Aplicado ao Desempenho e à Saúde, na UFPB. Em seguida você será avaliado por 8 semanas, podendo fazer parte do grupo experimental ou controle. O grupo experimental realizará 24 sessões de VGA com duração de 40 minutos, cada. O grupo controle não realizará as sessões de VGA, enquanto o estudo estiver sendo realizado, mas ao final das coletas, caso tenho interesse, poderá realizar as sessões se forma semelhante ao grupo experimental. Antes, após quatro semanas e após 8 semanas, estes procedimentos, será realizada inquérito nutricional (peso e gordura corporal, altura, circunferência da cintura e consumo alimentar) e da atividade autonômica cardíaca, aferição da pressão arterial e coletas sanguíneas.

Solicitamos a sua colaboração e autorização para coletar os dados necessários para esse estudo, assim como, apresentar os resultados obtidos em eventos da área de saúde e publicar em revistas científicas garantindo que seus dados pessoais serão mantidos em absoluto sigilo. Informamos que essa pesquisa não oferece riscos, previsíveis, para a sua saúde e que a sua participação no estudo é voluntária, portanto, o (a) senhor (a) não é obrigado (a) a fornecer as informações e/ou colaborar com as atividades solicitadas pelo pesquisador (a). Caso decida não participar do estudo ou resolver a qualquer momento desistir do mesmo, não sofrerá nenhum dano, nem haverá modificação na assistência que vem recebendo na Instituição.

Os pesquisadores estarão a sua disposição para qualquer esclarecimento que considere necessário em qualquer etapa da pesquisa. Diante do exposto, declaro que fui devidamente esclarecido (a) e dou o meu consentimento para participar da pesquisa e para publicação dos resultados. Estou ciente que receberei uma cópia desse documento.

__________________________________________

Assinatura do Participante da Pesquisa

ou Responsável Legal

Impressão datiloscópica

_______________________________________________

Assinatura da Testemunha

Contato com o Pesquisador (a) Responsável:

Caso necessite de maiores informações sobre o presente estudo, favor contatar o (a) pesquisador (a) TAÍS FEITOSA DA SILVA. Endereço: Rua Severina Crispim Veras, nº 571, CEP 58065-075, Planalto Boa Esperança, telefones: (83) 8808-0748 e (83) 9939-6441. Email: taisfsilva2@hotmail.com ou taisfs92@gmail.com.

Ou

Comitê de Ética em Pesquisa do Centro de Ciências da Saúde – Universidade Federal da Paraíba. Endereço: Centro de Ciências da Saúde – 1º andar/ Campus I/ Cidade Universitária/ CEP: 58051-900 – Bairro Castelo Branco – João Pessoa – PB. CNPJ: 24098477/007-05 – Telefone/Fax: (83) 3216-7791. Email: eticaccs@ccs.ufpb.br.

Atenciosamente,

______________________________________________

Assinatura do Pesquisador Responsável

Obs.: O participante e o pesquisador responsável deverão rubricar todas as folhas do TCLE apondo suas assinaturas na última página do referido Termo.

**APÊNDICE B**- Ficha individual para coleta de dados

DATA:____/____/_____.

**I- DADOS PESSOAIS**

Nome:________________________________________________________________

Endereço:_____________________________________________________________

Idade:_______________ Data de Nasc:_____/_____/_______

Profissão:__________________________ Tel. ________________________________

Escolaridade:___________________________________________________________

Local de pesquisa: ______________________________________________

Horários disponíveis para coleta de dados: ___________________________________

**II – HISTÓRIA CLÍNICA:**

Você é hipertenso? ( ) Sim ( ) Não

Você é diabético? ( ) Sim ( ) Não

Você é menopausadas? ( ) Sim ( ) Não A quanto tempo?_____________________

Faz uso de alguma medicação? ( ) Sim ( ) Não

Qual(is) medicamentos utiliza?

| Medicamento | Quantidade- dose | Horário-turno |
| --- | --- | --- |
|  |  |  |
|  |  |  |
|  |  |  |
|  |  |  |

É fumante? ( ) Sim ( ) Não. Há quanto tempo?_______ Quantos cigarros/dia?_____

Ingere bebidas alcoólicas? ( ) Sim ( ) Não. Frequência?______________________

Pratica exercício físico? ­( ) Sim ( ) Não.

Qual(is):___________________________

Frequência de prática:______________________

Tempo (min):________________________

Utiliza algum suplemento alimentar? ( ) Sim ( ) Não

Qual(is):____________________________________________________________________

Apresenta outras patologias? ( ) Sim ( ) Não. Qual(is):____________________________

Utiliza algum medicamento com ação no emagrecimento ou na inflamação?

( ) Sim ( ) Não. Qual(is):____________________________________________________

**III-** **MENSURAÇÃO DE MEDIDAS ANTROPOMÉTRICAS**

| Peso atual (kg) |  |
| --- | --- |
| Estatura (m) |  |
| % G |  |
| Índice de Massa Corpórea (IMC) |  |
| Circunferência da Cintura (cm) |  |

**IV- MENSURAÇÃO CLÍNICA DA PRESSÃO ARTERIAL**

| PAR-1 (mmHg) | PAR-2 (mmHg) |
| --- | --- |
|  |  |

V- VALORES DE VARIÁVEIS BIOQUÍMICAS DA TRIAGEM

| Glicemia (mg/dl) | Colesterol total (mg/dl) | LDL – c (mg/dl) | HDL – c (mg/dl) | Triglicerídeos (mg/dl) |
| --- | --- | --- | --- | --- |
|  |  |  |  |  |

APÊNDICE C – IPAQ – versão curta

**QUESTIONÁRIO INTERNACIONAL DE ATIVIDADE FÍSICA – VERSÃO CURTA –**

**Nome: _____________________________________________________________**

**Data: ______/ _______ / ______ Idade: ______ Sexo: F ( ) M ( )**

Nós estamos interessados em saber que tipos de atividade física as pessoas fazem como parte do seu dia a dia. Este projeto faz parte de um grande estudo que está sendo feito em diferentes países ao redor do mundo. Suas respostas nos ajudarão a entender que tão ativos nós somos em relação às pessoas de outros países. As perguntas estão relacionadas ao tempo que você gasta fazendo atividade física na **ÚLTIMA** semana. As perguntas incluem as atividades que você faz no trabalho, para ir de um lugar a outro, por lazer, por esporte, por exercício ou como parte das suas atividades em casa ou no jardim.

Suas respostas são MUITO importantes. Por favor responda cada questão mesmo que considere que não seja ativo. Obrigado pela sua participação!

Para responder as questões lembre-se que:

Atividades físicas **VIGOROSAS**são aquelas que precisam de um grande esforço físico e que fazem respirar MUITO mais forte que o normal;

Atividades físicas **MODERADAS**são aquelas que precisam de algum esforço físico e que fazem respirar UM POUCO mais forte que o normal;

Para responder as perguntas pense somente nas atividades que você realiza **por pelo menos 10 minutos contínuos**de cada vez:

**1ª**Em quantos dias da última semana você caminhou por pelo menos 10 minutos contínuos em casa ou no trabalho, como forma de transporte para ir de um lugar para outro, por lazer, por prazer ou como forma de exercício?

Dias ____por **SEMANA**( ) Nenhum

**1b**Nos dias em que você caminhou por pelo menos 10 minutos contínuos quanto tempo no

total você gastou caminhando **por dia**?

Horas: ____ Minutos: ____

**2ª.**Em quantos dias da última semana, você realizou atividades **MODERADAS**por pelo menos 10 minutos contínuos, como por exemplo pedalar leve na bicicleta, nadar, dançar, fazer ginástica aeróbica leve, jogar vôlei recreativo, carregar pesos leves, fazer serviços domésticos na casa, no quintal ou no jardim como varrer, aspirar, cuidar do jardim, ou qualquer atividade que fez aumentar **moderadamente**sua respiração ou batimentos do coração **(POR FAVOR NÃO INCLUA CAMINHADA)**

dias _____por **SEMANA**( ) Nenhum

**2b**. Nos dias em que você fez essas atividades moderadas por pelo menos 10 minutos

contínuos, quanto tempo no total você gastou fazendo essas atividades **por dia**?

Horas: ______ Minutos: _____

**3ª**Em quantos dias da última semana, você realizou atividades **VIGOROSAS**por pelo

menos 10 minutos contínuos, como por exemplo correr, fazer ginástica aeróbica, jogar

futebol, pedalar rápido na bicicleta, jogar basquete, fazer serviços domésticos pesados em casa, no quintal ou cavoucar no jardim, carregar pesos elevados ou qualquer atividade que fez aumentar **MUITO**sua respiração ou batimentos do coração.

Dias _____por **SEMANA**( ) Nenhum

**3b**Nos dias em que você fez essas atividades vigorosas por pelo menos 10 minutos

contínuos quanto tempo no total você gastou fazendo essas atividades **por dia**?

Horas: ______ Minutos: ____

Estas últimas questões são sobre o tempo que você permanece sentado, no trabalho, na escola ou faculdade, em casa e durante seu tempo livre. Isto inclui o tempo sentado

estudando, sentado enquanto descansa, fazendo lição de casa, visitando um amigo, lendo, sentado ou deitado assistindo TV. Não inclua o tempo gasto sentando durante o transporte de ônibus, trem, metrô ou carro.

**4a.**Quanto tempo, no total, você gasta sentado durante um **dia de semana de semana**?

______horas ____minutos

**4b.**Quanto tempo, no total, você gasta sentado durante um **dia de final de semana**?

______horas ____minutos

Fonte: MATSUDO et al. (2001).

APÊNDICE D – Recordatório Alimentar de 24 horas

1°( ) 2°( ) 3°( )

Nome:_______________________________________________ ____ Data___ /___ /_____

| Refeição/horário | Preparação e/ou alimentos | Medida caseira | Quantidade  (g/ml) | Observações |
| --- | --- | --- | --- | --- |
| Desjejum |  |  |  |  |
|  |  |  |  |  |
|  |  |  |  |  |
|  |  |  |  |  |
| Lanche |  |  |  |  |
|  |  |  |  |  |
|  |  |  |  |  |
| Almoço |  |  |  |  |
|  |  |  |  |  |
|  |  |  |  |  |
|  |  |  |  |  |
|  |  |  |  |  |
|  |  |  |  |  |
| Lanche |  |  |  |  |
|  |  |  |  |  |
|  |  |  |  |  |
|  |  |  |  |  |
| Jantar |  |  |  |  |
|  |  |  |  |  |
|  |  |  |  |  |
|  |  |  |  |  |
|  |  |  |  |  |
|  |  |  |  |  |
| Colação |  |  |  |  |
|  |  |  |  |  |
|  |  |  |  |  |

Preferências:___________________________________________________________

Aversões:_**_____________________________________________________________**

REGISTRO DE SINTOMAS: GASTROINTESTINAIS, ALERGIA E/OU INTOLERÂNCIA

| Constipação | **SIM ( ) NÃO ( )** | Diarréia | **SIM ( ) NÃO ( )** |
| --- | --- | --- | --- |
| Azia | **SIM ( ) NÃO ( )** | Náuseas | **SIM ( ) NÃO ( )** |
| Flatulência | **SIM ( ) NÃO ( )** | Alergias ou intolerâncias | **SIM ( ) NÃO ( )** |

**APÊNDICE E** – Lista de alimentos e medicamentos ricos em cafeína

63

| **Alimentos** | **Medicamentos** |
| --- | --- |
| Café de todos os tipos | Analgex |
| Achocolatado pronto | Antigripine |
| Bebidas a base de cacau | Antitermin |
| Chá branco | Benegrip |
| Cholocate ao leite | Beserol |
| Chá verde | Cafergot |
| Chocolate em pó | Cefalium |
| Refrigerante a base de cola | Cefaliv |
| Chá mate | Cibalena A |
| Bebida energética | Coristina D |
| Refrigerante a base de guaraná | Coristina R |
| Chocolate amargo | Dorflex |
| Xarope de chocolate | Doribel |
|  | Doril |
|  | Engov |
|  | Euforin |
|  | Excedrin |
|  | Fontol |
|  | Fontol 650 |
|  | Melhoral |
|  | Neosaldina |
|  | Optalidon |
|  | Ormigrein |
|  | Paceflex |
|  | Parcel |
|  | Resfriol |
|  | Sanacol |
|  | Saridon |
|  | Sedalex |
|  | Sedalgina |
|  | Sedilax |
|  | Sinutab |
|  | Sulindor |
|  | Tanderalgin |
|  | Tandriax |
|  | Tensilax |
|  | Tonopan |
|  | Torsilax |
|  | Trilax |
|  | Tylenol DC |

**APÊNDICE F** – *Enjoynment Scale*


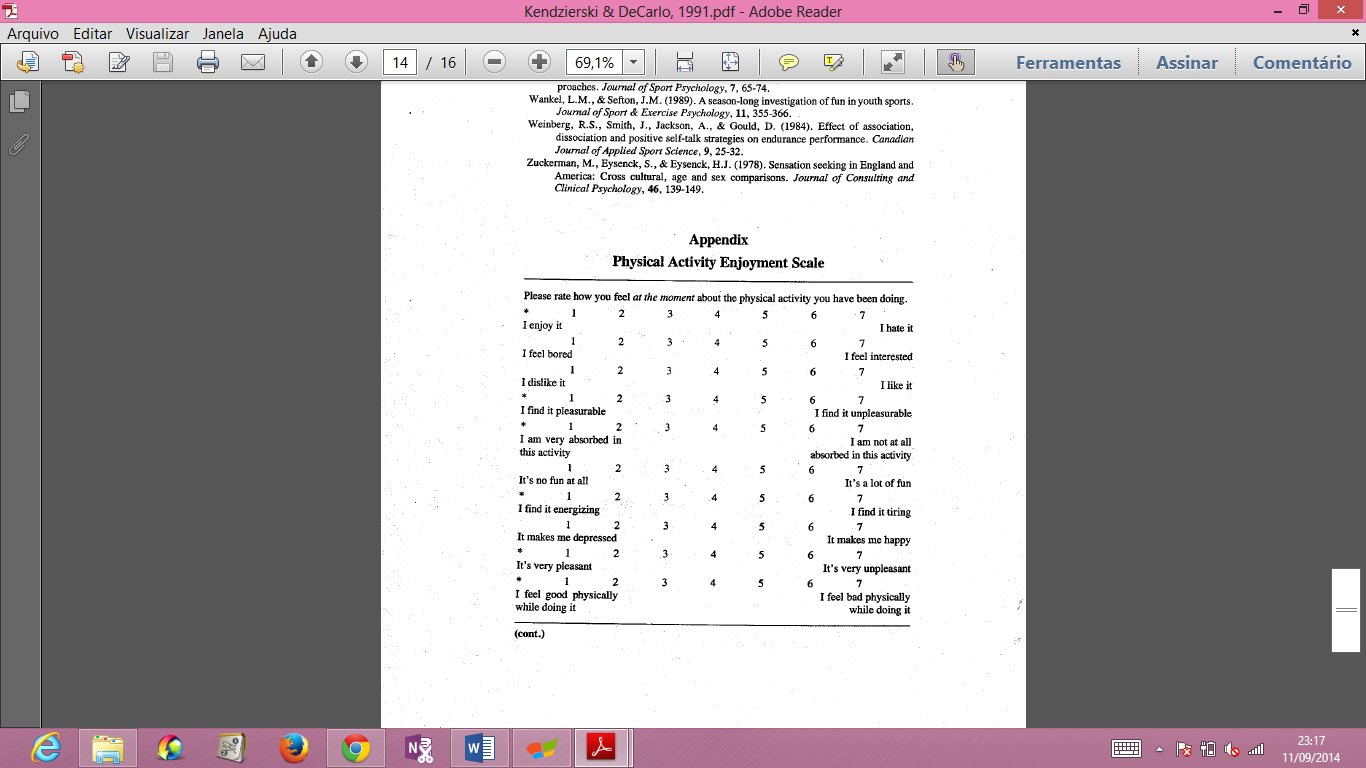

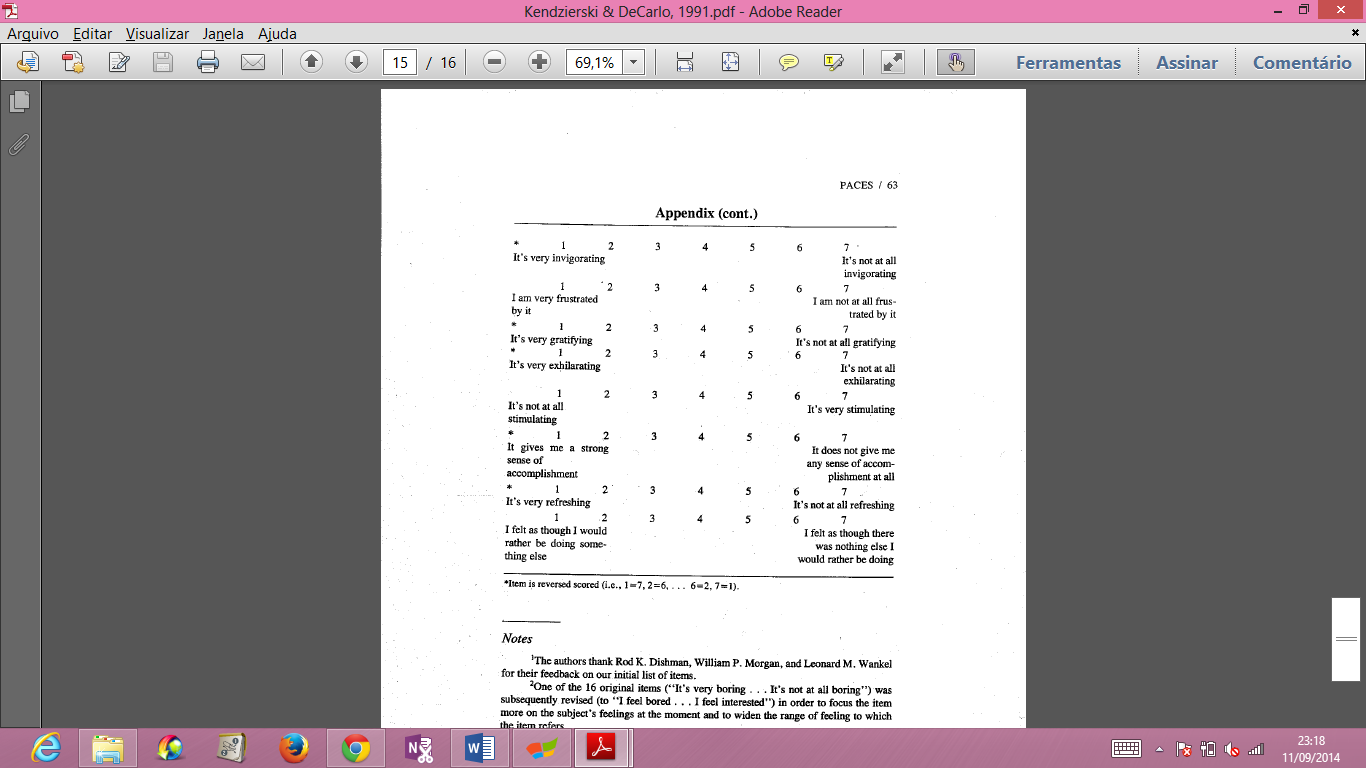


**ANEXOS**

ANEXO A – Questionário de Frequência de Consumo Alimentar

(Assinalar com X, N = nas refeições e E = entre as refeições (lanches, onde existir).

| **SOPAS e MASSAS** | **QUANTAS VEZES VOCE COME** | | | | | | | | | | | | | | | | | | | | **UNIDADE**  **1 2 3 4** | | **PORÇÃO MÉDIA(M)** | **SUA PORÇÃO**  **1 2 3 4** | 🡯não escrever aqui🡮 |
| --- | --- | --- | --- | --- | --- | --- | --- | --- | --- | --- | --- | --- | --- | --- | --- | --- | --- | --- | --- | --- | --- | --- | --- | --- | --- |
| Sopas (de legumes, canja, cremes etc.). | **N**  O | **1**  O | **2**  O | | **3**  O | | **4**  O | | **5**  O | | **6**  O | | **7**  O | | **8**  O | | **9**  O | | **10**  O | | | D S M A  O O O O | 2 conchas médias (260ml) | P M G E  O O O O | __ __ __ __ |
| Macarronada, lasanha. | **N**  O | **1**  O | **2**  O | | **3**  O | | **4**  O | | **5**  O | | **6**  O | | **7**  O | | **8**  O | | **9**  O | | **10**  O | | | D S M A  O O O O | 1 escumadeira rasa ou  ½ prato (75 g) | P M G E  O O O O | __ __ __ __ |
| Pizza. | **N**  O | **1**  O | **2**  O | | **3**  O | | **4**  O | | **5**  O | | **6**  O | | **7**  O | | **8**  O | | **9**  O | | **10**  O | | | D S M A  O O O O | 1 pedaço médio (130g) | P M G E  O O O O | __ __ __ __ |
| Pastelaria, empada, esfiha, pastel, kibe, coxinha. | **N**  O | **1**  O | **2**  O | | **3**  O | | **4**  O | | **5**  O | | **6**  O | | **7**  O | | **8**  O | | **9**  O | | **10**  O | | | D S M A  O O O O | 1 unidade ou 1 pedaço médio (60g) | P M G E  O O O O | __ __ __ __ |
| **CARNES E PEIXES** | QUANTAS VEZES VOCE COME | | | | | | | | | | | | | | | | | | | | **UNIDADE**  **1 2 3 4** | | **PORÇÃO MÉDIA(M)** | **SUA PORÇÃO**  **1 2 3 4** | 🡯não escrever aqui🡮 |
| Peixe cozido, assado ou grelhado. | **N**  O | **1**  O | | **2**  O | | **3**  O | | **4**  O | | **5**  O | | **6**  O | | **7**  O | | **8**  O | | **9**  O | | **10**  O | D S M A  O O O O | | 1 unidade grande ou 3 pedaços (100g) | P M G E  O O O O | __ __ __ __ |
| Peixe frito. | **N**  O | **1**  O | | **2**  O | | **3**  O | | **4**  O | | **5**  O | | **6**  O | | **7**  O | | **8**  O | | **9**  O | | **10**  O | D S M A  O O O O | | 1 posta média ou 1 filé médio (120 g) | P M G E  O O O O | __ __ __ __ |
| Carne de boi cozida, assada, grelhada, churrasco. | **N**  O | **1**  O | | **2**  O | | **3**  O | | **4**  O | | **5**  O | | **6**  O | | **7**  O | | **8**  O | | **9**  O | | **10**  O | D S M A  O O O O | | 3 fatias/pedaços ou 1 bife médio (100 g) | P M G E  O O O O | __ __ __ __ |
| Bife. | **N**  O | **1**  O | | **2**  O | | **3**  O | | **4**  O | | **5**  O | | **6**  O | | **7**  O | | **8**  O | | **9**  O | | **10**  O | D S M A  O O O O | | 1 unidade grande (150g) | P M G E  O O O O | __ __ __ __ |
| Carne de charque, carne de sol. | **N**  O | **1**  O | | **2**  O | | **3**  O | | **4**  O | | **5**  O | | **6**  O | | **7**  O | | **8**  O | | **9**  O | | **10**  O | D S M A  O O O O | | 1 pedaço grande (60g) | P M G E  O O O O | __ __ __ __ |
| Lingüiça, salsicha, presunto, outros frios. | **N**  O | **1**  O | | **2**  O | | **3**  O | | **4**  O | | **5**  O | | **6**  O | | **7**  O | | **8**  O | | **9**  O | | **10**  O | D S M A  O O O O | | 1 unidade, 1 gomo ou 2 fatias(40g) | P M G E  O O O O | __ __ __ __ |
| Frango frito, à milanesa, nuggets. | **N**  O | **1**  O | | **2**  O | | **3**  O | | **4**  O | | **5**  O | | **6**  O | | **7**  O | | **8**  O | | **9**  O | | **10**  O | D S M A  O O O O | | 2 pedaços ou 1 filé médio (90g) | P M G E  O O O O | __ __ __ __ |
| Frango guisado, grelhado,assado, espeto. | **N**  O | **1**  O | | **2**  O | | **3**  O | | **4**  O | | **5**  O | | **6**  O | | **7**  O | | **8**  O | | **9**  O | | **10**  O | D S M A  O O O O | | 3-4 pedaços médios (120g) | P M G E  O O O O | __ __ __ __ |
| Miúdos de frango. | **N**  O | **1**  O | | **2**  O | | **3**  O | | **4**  O | | **5**  O | | **6**  O | | **7**  O | | **8**  O | | **9**  O | | **10**  O | D S M A  O O O O | | 3 pedaços(60g) | P M G E  O O O O | __ __ __ __ |
| Fígado bovino. | **N**  O | **1**  O | | **2**  O | | **3**  O | | **4**  O | | **5**  O | | **6**  O | | **7**  O | | **8**  O | | **9**  O | | **10**  O | D S M A  O O O O | | 1 filé médio (60 g) | P M G E  O O O O | __ __ __ __ |
| **LEGUMINOSAS**  **E OVOS** | **QUANTAS VEZES VOCE COME** | | | | | | | | | | | | | | | | | | | | | **UNIDADE**  **1 2 3 4** | **PORÇÃO MÉDIA (M)** | **SUA PORÇÃO**  **1 2 3 4** | 🡯não escrever aqui🡮 |
| Feijão roxo, carioca, preto, verde. | **N**  O | **1**  O | | **2**  O | | **3**  O | | **4**  O | | **5**  O | | **6**  O | | **7**  O | | **8**  O | | **9**  O | | **10**  O | D S M A  O O O O | | 1 concha média ou  4 colheres de sopa (90g) | P M G E  O O O O | __ __ __ __ |
| Ovos (cozido, cru, frito). | **N**  O | **1**  O | | **2**  O | | **3**  O | | **4**  O | | **5**  O | | **6**  O | | **7**  O | | **8**  O | | **9**  O | | **10**  O | D S M A  O O O O | | 1 unidade média (60g) | P M G E  O O O O | __ __ __ __ |
| Milho verde, ervilha, vagem (fresco, cong. ou enlatado). | **N**  O | **1**  O | | **2**  O | | **3**  O | | **4**  O | | **5**  O | | **6**  O | | **7**  O | | **8**  O | | **9**  O | | **10**  O | D S M A  O O O O | | 2 colheres de sopa (60g) | P M G E  O O O O | __ __ __ __ |
| **ARROZ E TUBÉRCULOS** | **QUANTAS VEZES VOCE COME** | | | | | | | | | | | | | | | | | | | | **UNIDADE**  **1 2 3 4** | | **PORÇÃO MÉDIA**  **(M)** | **SUA PORÇÃO**  **1 2 3 4** | 🡯não escrever aqui🡮 |
| Arroz branco cozido com óleo e temperos. | **N**  O | **1**  O | | **2**  O | | **3**  O | | **4**  O | | **5**  O | | **6**  O | | **7**  O | | **8**  O | | **9**  O | | **10**  O | D S M A  O O O O | | 3-4 colheres de sopa (90g) | P M G E  O O O O | __ __ __ __ |
| Batata frita ou mandioca frita. | **N**  O | **1**  O | | **2**  O | | **3**  O | | **4**  O | | **5**  O | | **6**  O | | **7**  O | | **8**  O | | **9**  O | | **10**  O | D S M A  O O O O | | 2 colheres de sopa (50g) | P M G E  O O O O | __ __ __ __ |
| Batata, mandioca, inhame - assado/cozido. | **N**  O | **1**  O | | **2**  O | | **3**  O | | **4**  O | | **5**  O | | **6**  O | | **7**  O | | **8**  O | | **9**  O | | **10**  O | D S M A  O O O O | | 3 pedaços médios(180g) | P M G E  O O O O | __ __ __ __ |
| Salada de maionese com legumes. | **N**  O | **1**  O | | **2**  O | | **3**  O | | **4**  O | | **5**  O | | **6**  O | | **7**  O | | **8**  O | | **9**  O | | **10**  O | D S M A  O O O O | | 3 colheres de sopa (90g) | P M G E  O O O O | __ __ __ __ |
| Batata doce ou abóbora. | **N**  O | **1**  O | | **2**  O | | **3**  O | | **4**  O | | **5**  O | | **6**  O | | **7**  O | | **8**  O | | **9**  O | | **10**  O | D S M A  O O O O | | 3 pedaços médios ou  1 unidade média (90g) | P M G E  O O O O | __ __ __ __ |
| Farofa, farinha de mandioca. | **N**  O | **1**  O | | **2**  O | | **3**  O | | **4**  O | | **5**  O | | **6**  O | | **7**  O | | **8**  O | | **9**  O | | **10**  O | D S M A  O O O O | | 2 colheres de sopa (30g) | P M G E  O O O O | __ __ __ __ |
| Cuscuz de milho ou com leite, angu, pirão, canjica. | **N**  O | **1**  O | | **2**  O | | **3**  O | | **4**  O | | **5**  O | | **6**  O | | **7**  O | | **8**  O | | **9**  O | | **10**  O | D S M A  O O O O | | 1 pedaço médio (135g) | P M G E  O O O O | __ __ __ __ |

| **LEITE E DERIVADOS, CEREAIS MATINAIS** | **QUANTAS VEZES VOCE COME** | | | | | | | | | | | **UNIDADE**  **1 2 3 4** | **PORÇÃO MÉDIA**  **(M)** | **SUA PORÇÃO**  **1 2 3 4** | 🡯não escrever aqui🡮 |
| --- | --- | --- | --- | --- | --- | --- | --- | --- | --- | --- | --- | --- | --- | --- | --- |
| Leite. Tipo: ( ) integral  ( ) desnat. ( ) semidesnat. | **N**  O | **1**  O | **2**  O | **3**  O | **4**  O | **5**  O | **6**  O | **7**  O | **8**  O | **9**  O | **10**  O | D S M A  O O O O | 1 copo (150 ml) | P M G E  O O O O | __ __ __ __ |
| Açúcar adicionado ao leite.  **( ) N ( ) E** | **N**  O | **1**  O | **2**  O | **3**  O | **4**  O | **5**  O | **6**  O | **7**  O | **8**  O | **9**  O | **10**  O | D S M A  O O O O | 3 colheres de chá (12g) | P M G E  O O O O | __ __ __ __ |
| Neston, aveia. | **N**  O | **1**  O | **2**  O | **3**  O | **4**  O | **5**  O | **6**  O | **7**  O | **8**  O | **9**  O | **10**  O | D S M A  O O O O | 1,5 colher de sopa (18g) | P M G E  O O O O | __ __ __ __ |
| Iogurte ou coalhada tipo:  ( ) natural ( ) com frutas | **N**  O | **1**  O | **2**  O | **3**  O | **4**  O | **5**  O | **6**  O | **7**  O | **8**  O | **9**  O | **10**  O | D S M A  O O O O | 1 copo americano (165ml) | P M G E  O O O O | __ __ __ __ |
| Vitamina de leite ou leite batido com fruta. | **N**  O | **1**  O | **2**  O | **3**  O | **4**  O | **5**  O | **6**  O | **7**  O | **8**  O | **9**  O | **10**  O | D S M A  O O O O | 1 copo (150 ml) | P M G E  O O O O | __ __ __ __ |
| Queijo minas ou ricota, requeijão light. **( )N ( )E** | **N**  O | **1**  O | **2**  O | **3**  O | **4**  O | **5**  O | **6**  O | **7**  O | **8**  O | **9**  O | **10**  O | D S M A  O O O O | 1 fatia peq. ou 1 colher de sopa rasa (20g) | P M G E  O O O O | __ __ __ __ |
| Queijo coalho, mant. prato, mussa, requeijão.  **( ) N ( ) E** | **N**  O | **1**  O | **2**  O | **3**  O | **4**  O | **5**  O | **6**  O | **7**  O | **8**  O | **9**  O | **10**  O | D S M A  O O O O | 2 fatias médias ou 1 colher de sopa (30g) | P M G E  O O O O | __ __ __ __ |
| **VEGETAIS** | **QUANTAS VEZES VOCE COME** | | | | | | | | | | | **UNIDADE**  **1 2 3 4** | **PORÇÃO MÉDIA**  **(M)** | **SUA PORÇÃO**  **1 2 3 4** | 🡯não escrever aqui🡮 |
| Alface. | **N**  O | **1**  O | **2**  O | **3**  O | **4**  O | **5**  O | **6**  O | **7**  O | **8**  O | **9**  O | **10**  O | D S M A  O O O O | 3 folhas médias (30g) | P M G E  O O O O | __ __ __ __ |
| Tomate cru. | **N**  O | **1**  O | **2**  O | **3**  O | **4**  O | **5**  O | **6**  O | **7**  O | **8**  O | **9**  O | **10**  O | D S M A  O O O O | 1 unidade pequena ou  4 fatias (70g) | P M G E  O O O O | __ __ __ __ |
| Couve, espinafre, cozido. | **N**  O | **1**  O | **2**  O | **3**  O | **4**  O | **5**  O | **6**  O | **7**  O | **8**  O | **9**  O | **10**  O | D S M A  O O O O | 3 colheres de sopa (60g) | P M G E  O O O O | __ __ __ __ |
| Beterraba, crua ou cozida. | **N**  O | **1**  O | **2**  O | **3**  O | **4**  O | **5**  O | **6**  O | **7**  O | **8**  O | **9**  O | **10**  O | D S M A  O O O O | 4 fatias ou 2,5 colheres de sopa (50g) | P M G E  O O O O | __ __ __ __ |
| Cenoura crua ou cozida. | **N**  O | **1**  O | **2**  O | **3**  O | **4**  O | **5**  O | **6**  O | **7**  O | **8**  O | **9**  O | **10**  O | D S M A  O O O O | 2 fatias ou 2colheres de sopa (30g) | P M G E  O O O O | __ __ __ __ |
| Pepino, pimentão. | **N**  O | **1**  O | **2**  O | **3**  O | **4**  O | **5**  O | **6**  O | **7**  O | **8**  O | **9**  O | **10**  O | D S M A  O O O O | 2 colheres de sopa (20g) | P M G E  O O O O | __ __ __ __ |
| **MOLHOS** | **QUANTAS VEZES VOCE COME** | | | | | | | | | | | **UNIDADE**  **1 2 3 4** | **PORÇÃO MÉDIA**  **(M)** | **SUA PORÇÃO**  **1 2 3 4** | 🡯não escrever aqui🡮 |
| Óleo, azeite ou vinagrete em saladas. | **N**  O | **1**  O | **2**  O | **3**  O | **4**  O | **5**  O | **6**  O | **7**  O | **8**  O | **9**  O | **10**  O | D S M A  O O O O | 3 colheres de sobremesa (15g) | P M G E  O O O O | __ __ __ __ |
| *Catchup* ou mostarda. | **N**  O | **1**  O | **2**  O | **3**  O | **4**  O | **5**  O | **6**  O | **7**  O | **8**  O | **9**  O | **10**  O | D S M A  O O O O | 1 colher de sopa (10g) | P M G E  O O O O | __ __ __ __ |
| Maionese, molho rosê (também em pães). | **N**  O | **1**  O | **2**  O | **3**  O | **4**  O | **5**  O | **6**  O | **7**  O | **8**  O | **9**  O | **10**  O | D S M A  O O O O | 1 colher de sopa (15g) | P M G E  O O O O | __ __ __ __ |
| **FRUTAS E SUCOS** | **QUANTAS VEZES VOCE COME** | | | | | | | | | | | **UNIDADE**  **1 2 3 4** | **PORÇÃO MÉDIA**  **(M)** | **SUA PORÇÃO**  **1 2 3 4** | 🡯não escrever aqui🡮 |
| Laranja, mexerica. | **N**  O | **1**  O | **2**  O | **3**  O | **4**  O | **5**  O | **6**  O | **7**  O | **8**  O | **9**  O | **10**  O | D S M A  O O O O | 2 unid. pequenas (180g) | P M G E  O O O O | __ __ __ __ |
| Banana. | **N**  O | **1**  O | **2**  O | **3**  O | **4**  O | **5**  O | **6**  O | **7**  O | **8**  O | **9**  O | **10**  O | D S M A  O O O O | 1 unidade média (60g) | P M G E  O O O O | __ __ __ __ |
| Mamão. | **N**  O | **1**  O | **2**  O | **3**  O | **4**  O | **5**  O | **6**  O | **7**  O | **8**  O | **9**  O | **10**  O | D S M A  O O O O | 1 fatia grande ou meio papaya (180g) | P M G E  O O O O | __ __ __ __ |
| Maçã. | **N**  O | **1**  O | **2**  O | **3**  O | **4**  O | **5**  O | **6**  O | **7**  O | **8**  O | **9**  O | **10**  O | D S M A  O O O O | 1 unidade média (130g) | P M G E  O O O O | __ __ __ __ |
| Melancia, melão. | **N**  O | **1**  O | **2**  O | **3**  O | **4**  O | **5**  O | **6**  O | **7**  O | **8**  O | **9**  O | **10**  O | D S M A  O O O O | 1 fatia média (150 g ) | P M G E  O O O O | __ __ __ __ |
| Manga (na época). | **N**  O | **1**  O | **2**  O | **3**  O | **4**  O | **5**  O | **6**  O | **7**  O | **8**  O | **9**  O | **10**  O | D S M A  O O O O | 1 unidade grande (220g) | P M G E  O O O O | __ __ __ __ |
| Abacaxi. | **N**  O | **1**  O | **2**  O | **3**  O | **4**  O | **5**  O | **6**  O | **7**  O | **8**  O | **9**  O | **10**  O | D S M A  O O O O | 2,5 fatias médias (260g) | P M G E  O O O O | __ __ __ __ |
| Goiaba (na época). | **N**  O | **1**  O | **2**  O | **3**  O | **4**  O | **5**  O | **6**  O | **7**  O | **8**  O | **9**  O | **10**  O | D S M A  O O O O | 1 unidade pequena (60g) | P M G E  O O O O | __ __ __ __ |
| Suco de caju(na época).  **( )N ( )E** | **N**  O | **1**  O | **2**  O | **3**  O | **4**  O | **5**  O | **6**  O | **7**  O | **8**  O | **9**  O | **10**  O | D S M A  O O O O | 1 copo (200ml) | P M G E  O O O O | __ __ __ __ |
| Suco de acerola.  **( )N ( )E** | **N**  O | **1**  O | **2**  O | **3**  O | **4**  O | **5**  O | **6**  O | **7**  O | **8**  O | **9**  O | **10**  O | D S M A  O O O O | 1 copo (200ml) | P M G E  O O O O | __ __ __ __ |
| Suco de laranja natural.  **( )N ( )E** | **N**  O | **1**  O | **2**  O | **3**  O | **4**  O | **5**  O | **6**  O | **7**  O | **8**  O | **9**  O | **10**  O | D S M A  O O O O | 1 copo (200ml) | P M G E  O O O O | __ __ __ __ |
| Suco natural de outras frutas | **N**  O | **1**  O | **2**  O | **3**  O | **4**  O | **5**  O | **6**  O | **7**  O | **8**  O | **9**  O | **10**  O | D S M A  O O O O | 1 copo (200ml) | P M G E  O O O O | __ __ __ __ |

| **PÃES E BISCOITOS** | **QUANTAS VEZES VOCE COME** | | | | | | | | | | | **UNIDADE**  **1 2 3 4** | **PORÇÃO MÉDIA**  **(M)** | **SUA PORÇÃO**  **1 2 3 4** | 🡯não escrever aqui🡮 |
| --- | --- | --- | --- | --- | --- | --- | --- | --- | --- | --- | --- | --- | --- | --- | --- |
| Pão francês, pão de forma, integral, pão doce, torrada. | **N**  O | **1**  O | **2**  O | **3**  O | **4**  O | **5**  O | **6**  O | **7**  O | **8**  O | **9**  O | **10**  O | D S M A  O O O O | 1 unidade ou 2 fatias (50g) | P M G E  O O O O | __ __ __ __ |
| Biscoito salgado, Biscoito doce *sem recheio.* | **N**  O | **1**  O | **2**  O | **3**  O | **4**  O | **5**  O | **6**  O | **7**  O | **8**  O | **9**  O | **10**  O | D S M A  O O O O | 5 a 6 unidades (30g) | P M G E  O O O O | __ __ __ __ |
| Biscoito doce *recheado,amanteigado.* | **N**  O | **1**  O | **2**  O | **3**  O | **4**  O | **5**  O | **6**  O | **7**  O | **8**  O | **9**  O | **10**  O | D S M A  O O O O | 3unidades (40g) | P M G E  O O O O | __ __ __ __ |
| Margarina passada no pão  ( ) comum ( ) light. | **N**  O | **1**  O | **2**  O | **3**  O | **4**  O | **5**  O | **6**  O | **7**  O | **8**  O | **9**  O | **10**  O | D S M A  O O O O | 2 pontas de faca (5g) | P M G E  O O O O | __ __ __ __ |
| Manteiga passada no pão. | **N**  O | **1**  O | **2**  O | **3**  O | **4**  O | **5**  O | **6**  O | **7**  O | **8**  O | **9**  O | **10**  O | D S M A  O O O O | 2 pontas de faca (5g) | P M G E  O O O O | __ __ __ __ |
| **BEBIDAS** | **QUANTAS VEZES VOCE COME** | | | | | | | | | | | **UNIDADE**  **1 2 3 4** | **PORÇÃO MÉDIA**  **(M)** | **SUA PORÇÃO**  **1 2 3 4** | 🡯não escrever aqui🡮 |
| Cerveja. | **N**  O | **1**  O | **2**  O | **3**  O | **4**  O | **5**  O | **6**  O | **7**  O | **8**  O | **9**  O | **10**  O | D S M A  O O O O | 1 lata (350 ml) ou 2 copos americanos | P M G E  O O O O | __ __ __ __ |
| Cachaça, whisky, vodka. | **N**  O | **1**  O | **2**  O | **3**  O | **4**  O | **5**  O | **6**  O | **7**  O | **8**  O | **9**  O | **10**  O | D S M A  O O O O | 2 doses (60 ml) | P M G E  O O O O | __ __ __ __ |
| Vinho. | **N**  O | **1**  O | **2**  O | **3**  O | **4**  O | **5**  O | **6**  O | **7**  O | **8**  O | **9**  O | **10**  O | D S M A  O O O O | 2 cálices de vinho ou um copo (120 ml) | P M G E  O O O O | __ __ __ __ |
| Café com açúcar.  **( ) N ( ) E** | **N**  O | **1**  O | **2**  O | **3**  O | **4**  O | **5**  O | **6**  O | **7**  O | **8**  O | **9**  O | **10**  O | D S M A  O O O O | 1 xícara de chá grande  (200 ml) | P M G E  O O O O | __ __ __ __ |
| Café sem açúcar. | **N**  O | **1**  O | **2**  O | **3**  O | **4**  O | **5**  O | **6**  O | **7**  O | **8**  O | **9**  O | **10**  O | D S M A  O O O O | 1 xícara de chá grande  (200 ml) | P M G E  O O O O | __ __ __ __ |
| Adoçante artificial. | **N**  O | **1**  O | **2**  O | **3**  O | **4**  O | **5**  O | **6**  O | **7**  O | **8**  O | **9**  O | **10**  O | D S M A  O O O O | 3 a 4 gotas ou 1 envelope (0,8g) | P M G E  O O O O | __ __ __ __ |
| **DOCES, SOBREMESAS E APERITIVOS** | **QUANTAS VEZES VOCE COME** | | | | | | | | | | | **UNIDADE**  **1 2 3 4** | **PORÇÃO MÉDIA**  **(M)** | **SUA PORÇÃO**  **1 2 3 4** | 🡯não escrever aqui🡮 |
| Chocolates, bombons, brigadeiro. **( ) N ( ) E** | **N**  O | **1**  O | **2**  O | **3**  O | **4**  O | **5**  O | **6**  O | **7**  O | **8**  O | **9**  O | **10**  O | D S M A  O O O O | 2 unidades ou 1 barra (30g) | P M G E  O O O O | __ __ __ __ |
| Doces de frutas. **( ) N ( ) E** | **N**  O | **1**  O | **2**  O | **3**  O | **4**  O | **5**  O | **6**  O | **7**  O | **8**  O | **9**  O | **10**  O | D S M A  O O O O | 1 pedaço médio (60g) | P M G E  O O O O | __ __ __ __ |
| Bolos e tortas. **( ) N ( ) E** | **N**  O | **1**  O | **2**  O | **3**  O | **4**  O | **5**  O | **6**  O | **7**  O | **8**  O | **9**  O | **10**  O | D S M A  O O O O | 1 fatia média (50g) | P M G E  O O O O | __ __ __ __ |
| Sorvete. **( ) N ( ) E** | **N**  O | **1**  O | **2**  O | **3**  O | **4**  O | **5**  O | **6**  O | **7**  O | **8**  O | **9**  O | **10**  O | D S M A  O O O O | 2 picolés ou 1 taça (2 bolas) (120g) | P M G E  O O O O | __ __ __ __ |
| Doce de abóbora ou goiabada (em lata ou caseiro. **( )N ( )E** | **N**  O | **1**  O | **2**  O | **3**  O | **4**  O | **5**  O | **6**  O | **7**  O | **8**  O | **9**  O | **10**  O | D S M A  O O O O | 1 pedaço pequeno (35g) | P M G E  O O O O | __ __ __ __ |
| Pipoca, salgadinhos, *chips,* torresmo. | **N**  O | **1**  O | **2**  O | **3**  O | **4**  O | **5**  O | **6**  O | **7**  O | **8**  O | **9**  O | **10**  O | D S M A  O O O O | 1 porção (45g) | P M G E  O O O O | __ __ __ __ |
| Refrigerantes. Tipo:  ( ) não-dietéticos ( ) dietéticos  **( )N ( )E . Usa canudo ( )** | **N**  O | **1**  O | **2**  O | **3**  O | **4**  O | **5**  O | **6**  O | **7**  O | **8**  O | **9**  O | **10**  O | D S M A  O O O O | 1 copo de 200 ml | P M G E  O O O O | __ __ __ __ |

| Quando você come carne bovina ou de porco, você costuma comer a gordura visível? | | | | |  |  | |  |  |  |
| --- | --- | --- | --- | --- | --- | --- | --- | --- | --- | --- |
| (1) nunca ou raramente (2) algumas vezes (3) sempre (9) não sabe | | | | |  |  | |  |  |  |
| Quando você come carne de frango ou peru, você costuma comer a pele? | | | | |  |  | |  |  |  |
| (1) nunca ou raramente (2) algumas vezes (3) sempre (9) não sabe | | | | |  |  | |  |  |  |
| Condimentos comprados por mês (quantidade): | | | | |  | | |  |  |  |
| Óleo de soja (ml) | | | | |  | | |  |  |  |
| Vinagre (ml) | | | | |  | | |  |  |  |
| Sal (kg) | | | | |  | | |  |  |  |
| Açúcar (kg) | | | | |  | | |  |  |  |
| Por favor, liste qualquer outro alimento ou preparação importante que você costuma comer ou beber pelo menos **UMA VEZ** **POR SEMANA** que não foram citados aqui (por exemplo: fibrax, leite-de-coco, outros tipos de carnes, receitas caseiras, creme de leite, leite condensado, gelatina e outros doces etc. ). | | |  | 🡯não escrever aqui🡮 | | | | | | |
| ALIMENTO | FREQUÊNCIA POR SEMANA | QUANTIDADE CONSUMIDA |  | COD | | | CONS | | | |
|  |  |  |  | ___ ___ | | | ___ ___ ___ __ | | | |

**ANEXO B –** Lista de músicas componentes do jogo *Just Dance Summer Party*

| Austin Mahone - 'What About Love' | Robin Thicke ft. Pharrell Williams - 'Blurred Lines Alt' | Chris Brown - "Fine China" | Gloria Gaynor - "I Will Survive" |
| --- | --- | --- | --- |
| Pitbull ft. Ke$ha - 'Timber' | Macklemore & Ryan Lewis ft. Ray Dalton - "Can’t Hold Us" | Ray Parker Jr. - "Ghostbusters" | Jessie J Ft. Big Sean - "Wild" |
| Ke$ha - 'Die Young' | Avicii - "’Wake Me Up’’ | George Michael - "Careless Whisper" | ABBA - "Gimme! Gimme! Gimme! (A Man After Midnight)" |
| Lady Gaga ft. Colby O'Donis - 'Just Dance' (Alt. Version) | Pink - 'Funhouse' | Katy Perry - "I Kissed A Girl" | Bob Marley - "Could you be loved" |
| Katy Perry - "Roar" | Katy Perry - 'Part of Me' | Gwen Stefani ft. Eve - "Rich Girl" | Ricky Martin - "Maria" |
| Avril Lavigne - 'Rock N Roll' | Lady Gaga - "Applause" | Mick Jackson - "Blame It On The Boogie" | Bog Bog Orkestar - "Isidora" |
| MainStreet - 'My Main Girl' | Lady Gaga ft. Colby O'Donis - "Just Dance" | The Village People - "YMCA" | Mungo Jerry - "In The Summertime" |
| Swedish House Mafia ft. John Martin - 'Don't You Worry Child' | Jason Derulo - "The Other Side" | David Guetta ft. Sia - "She Wolf (Falling to Pieces)" | Disney's Aladdin - "Prince Ali" |
| Calvin Harris ft. Ellie Goulding - 'I Need Your Love' | Ariana Grande ft. Mac Miller - "The Way" | Pitbull ft. Christina Aguilera - "Feel This Moment" | The Girly Team - "Flashdance...What A Feeling" |
| Becky G ft. Pitbull - 'Can't Get Enough' | Daft Punk ft. Pharrell Williams - "Get Lucky" | Nicki Minaj - "Pound The Alarm" | The Sunlight Shakers - "Aquarius/Let The Sunshine In" |
| Lady Gaga - 'Applause (Official Choreography)' | Robin Thicke ft. Pharrell Williams - "Blurred Lines" | One Direction - "Kiss You" | Duck Sauce - "It's You" |
| PSY - 'Gangnam Style' | Daddy Yankee - "Limbo" | Psy - "Gentleman" | Dancing Bros.- "Moskau" |
| Will.i.am ft. Justin Bieber - '#thatPOWER (Alt. Choreography)' | Wisin & Yandel ft. Jennifer Lopez - "Follow the Leader" | Nicki Minaj - "Starships" | Frankie Bostello - "The Love Boat" |
| Bonnie McKee - 'American Girl' | Rihanna - "Where Have You Been" | Olly Murs ft. Flo Rida - "Trouble Maker" | Imposs Ft. Konshens - "Feel So Right" |
| One Direction - 'One Way or Another' | Will.i.am ft Justin Bieber - "#thatPower" | Far East Movement ft. Cover Drive - "Turn Up The Love" | Ivete Sangalo - "Dançando" |
| LMFAO - 'Sexy and I Know It' | Kesha - "C'mon" | Robbie Williams - "Candy" | Rutschen Planeten - "99 Luftballons" |
| Sammie - "Miss Understood " | Sentai Express - "Nitro Bot" | Louis Prima - "Just A Gigolo" |  |

**Anexo C –** Diário de atividades (MAPA)


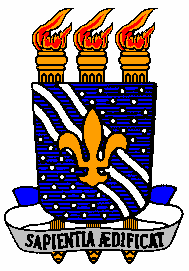
**UNIVERSIDADE FEDERAL DA PARAÍBA**

**CENTRO DE CIÊNCIAS DA SAÚDE**

**PROGRAMA DE PÓS-GRADUAÇÃO EM CIÊNCIAS DA NUTRIÇÃO**

LABORATÓRIO DE ESTUDOS DO TREINAMENTO FÍSICO APLICADO AO DESEMPENHO E À SAÚDE**- LETFADS**

**DIÁRIO DE ATIVIDADES (MAPA)**

Nº exame ________ ( )PRÉ ( )PÓS

Instalado por: ______________________________________________________

Data: _____/______/ ______

Nome do paciente: _________________________________________________

Telefone: ( ) __________________________

Endereço: ________________________________________________________

**Em caso de dúvida telefonar para:**

**Taís Feitosa da Silva – 8808-0748/ 9939-6441**

**ORIENTAÇÕES AO PACIENTE - MAPA**

1. **A cada medida** da pressão arterial:

- **Manter o braço relaxado** e estendido ao longo do corpo. Se estiver sentado, apoiar o antebraço em um suporte qualquer (mesa, cadeira, etc).

- Evitar movimentos de abrir e fechar a mão ou movimentar os dedos.

1. **Não retire o manguito** do braço durante o período de exame.
2. Não realizar exercício físico no dia do exame, nem no dia anterior. **Manter as atividades normais**, com o cuidado de permanecer parado no momento da medição**.**
3. Tome banho antes de vir colocar o aparelho, porque **você não poderá tomar banho** durante as 24 horas do exame. O equipamento não deverá ser molhado e nem queimado.
4. Use blusa ou camisa com manga larga ou sem manga, frouxa e fácil de vestir.
5. Quando for dormir, procure colocar o aparelho ao lado do travesseiro, para que ele não atrapalhe seu sono. Cuidado para não deitar por cima da borracha do manguito.
6. **Não dormir durante o dia**.
7. Cuidado para não deitar sobre o braço que está com o manguito.
8. **Preencha o diário** nos momentos de: almoço, jantar, sono (hora em que dormiu e acordou), trabalhou. Se sentir alguma coisa fora do normal, anotar nas observações e seu horário.
9. Anotar os horários que consumiu bebidas alcoólicas, café e cigarros.
10. Se você está usando medicamentos, não se esquecer de tomá-los nos horários certos e de anotar no diário (nome dos remédios e hora em que tomou).
11. Caso aconteça falha na medição, o aparelho fará outra medida 3 minutos após.

*Anote na tabela ao lado as atividades realizadas por você e o horário em que elas ocorreram.*

***OBS: utilizar a hora mostrada no aparelho***

| **HORÁRIO** | **ATIVIDADE REALIZADA** |
| --- | --- |
|  |  |
|  |  |
|  |  |
|  |  |
|  |  |
|  |  |
|  |  |
|  |  |
|  |  |
|  |  |
|  |  |
|  |  |
|  |  |
|  |  |
|  |  |
|  |  |
|  |  |
|  |  |
|  |  |
|  |  |
|  |  |
|  |  |
|  |  |
|  |  |
|  |  |
| Observações: | |

**TRADUTION**

FEDERAL UNIVERSITY OF PARAIBA

PROGRAMA ASSOCIADO DE PÓS-GRADUAÇÃO EM EDUCAÇÃO FÍSICA

UPE/UFPB

Efficacy of active video game on blood pressure and glucose in diabetic hypertensive patients: acute and chronic effects

JOÃO PESSOA

2014

TAÍS FEITOSA DA SILVA

Project presented to the Research Ethics Committee, under the guidance of Professor Dr. Alexandre Sérgio Silva for the development of a research project in the Laboratory of Studies of Physical Training Applied to Performance and Health - LETFADS, Department of Physical Education

Efficacy of active video game on blood pressure and glucose in diabetic hypertensive patients: acute and chronic effects

Master’s Advisor: Alexandre Sergio Silva

JOÃO PESSOA

2014

1. INTRODUCTION

It is a consensus in the literature on the importance of practicing physical activity performed on a regular basis for human health (Haskell et al., 2007; SBC, SBH, SBN, 2010; HENRIQUES, 2013). However, the development of technological resources has contributed to the practice of sedentary behavior, including in the area of leisure, where activities such as playing video games replace activities as sports (PITANGA and LESSA, 2005). Nonetheless, a market trend has developed for the development of active video games, a tool that can be considered as an alternative to the practice of sedentary leisure activities (STAIANO and CALVERT, 2011).

Active video game (AVG), also called exergame or video game physically interactive, is conceptualized as a game in which the practitioner replaces the traditional joystick with a sensor that leads him to perform the movements that he wants to be performed by the avatar on the screen (STAIANO and CALVERT, 2011). The energy expenditure resulting from the practice of these games is significantly increased when compared to sedentary video game (VGS) as it has been demonstrated in children, adolescents (GRAVES et al., 2008; PEREIRA et al., 2012), young and elderly adults (Graves et al., 2010).

Despite the higher energy demand of the VGA when compared to SVG and its similarity to physical exercise, this demand is still below or below the minimum limits recommended by the main associations that study physical exercise (Haskell et al., 2007). While these institutions recommend moderate to vigorous exercise, which would be around 60% and 85% of maximal heart rate (MHR), the AVG’s impose, in most studies, an intensity below 55% of the MHR. And MILLS et al., 2013) and only in some studies has the intensity reached above 60% of the MHR, But not more than 75% of FCM (SIEGEL et al., 2009, O'DONOVAN and HUSSEY, 2012).

Because of the low physiological demands of these games, studies have been conducted to test the therapeutic power of AVG’s with people with low physical fitness. In studies by Broeren et al. (2008) and Garcia et al. (2010) improved motor performance when AVG was adopted in physical therapy rehabilitation after encephalic vascular accident in adults and elderly. In addition, a study by Maddison et al. (2011) who evaluated the practice of these games for 24 weeks on body composition in overweight and obese adolescents, showed a reduction in body mass index (BMI) and body fat, and the study by Madsen et al. (2007) who also evaluated overweight adolescents during the same period of practice did not find a reduction in BMI.

From the point of view of the treatment of hypertension and diabetes, it is known that the intensity is a variable of great importance. It is understood that the best blood pressure lowering (BP) effects are observed in exercises performed initially with intensity between 60% and 80% of the MHR, whereas for glycemic control exercises performed with intensity between 50% and 80 % Of FCM according to the treatment guidelines for these diseases (SBC, SBH, SBN, 2010, SBD, 2013/2014).

Although the recommendations for the treatment of hypertension propose a minimum intensity of 60% of the MHR (SBC, SBH, SBN, 2010), there are studies that demonstrate that exercises with lighter intensities are sufficient to promote BP reduction, as it is pointed out in the A study by Anunciação and Polito (2010), which stated that aerobic physical exercise performed at approximately 30% intensity of the MHR promoted post exercise exercise hypotension (PEH). Regarding diabetes, it should be noted that it is possible to find AVG games that impose the minimum demand necessary to promote blood glucose reduction. These assumptions provide the basis for the hypothesis that a AVG session is capable of promoting HPE and a AVG-based training program is capable of promoting BP and blood glucose reduction in hypertensive and diabetic women.

It is known that several markers influence the control of these diseases, such as autonomic nervous system, which is related to the cardiac output and peripheral vascular resistance (DE ANGELIS et al., 2004; MOSTARDA et al., 2009), besides being responsible By the action of vital organs like the pancreas that produces insulin. Another influencer is the occurrence of oxidative stress that is characterized as an unbalance in the redox system that leads to damage to blood vessels and important organs (Gottling et al., 2010). Like these, systemic inflammation is a common point of chronic diseases, since they originate from an inflammation in the body (VOLP et al., 2008).

When related to the practice of physical exercise to these markers, one can find studies that show a modulation of cardiac autonomic activity after exercise (LUNZ et al., 2013), as well as regulation of blood glucose independent of insulin production (ARSA) (Almeida et al., 2009), and a reduction in oxidative stress, with the increase of circulating antioxidant substances (ZANELLA et al., 2007) and reduced markers of systemic inflammation. Thus, in this study we will also test the hypothesis that the AVG can modify the behavior of these markers, being able to explain possible PEH and reduction of glycemia in response to sessions or training with AVG.

Therefore the purpose of this study is to evaluate the efficacy of a session and a training program with AVG on BP, cardiac autonomic modulation, glycemic and lipid profiles, systemic inflammation and oxidative stress in diabetic hypertensive women.

2. OBJECTIVES

2.1 General

To investigate the efficacy of a session and a training program with AVG on blood pressure and glycemic profile of diabetic hypertensive patients evaluating some guiding aspects, being, physiological demand, cardiac autonomic modulation, lipid profile, systemic inflammation and oxidative stress.

2.2 Specific

• Evaluate the energy demand and the sense of pleasure in a session with VGA and the beginning, middle and end of a training program;

• Evaluate the response of clinical blood pressure to a AVG session;

• To analyze the clinical and outpatient pressure response of diabetic hypertensive patients to AVG training;

• Analyze the response of cardiac autonomic modulation of diabetic hypertensive patients after an AVG session and at the beginning, middle and end of a training program;

• Analyze glycemic response and glycated hemoglobin of diabetic hypertensives at the beginning, middle and end of a AVG training program;

• Analyze the effect of AVG training on systemic inflammatory markers;

• Analyze the effect of AVG training on oxidative stress markers.

3. QUESTION OF THE STUDY

Can a session and / or a training protocol with AVG be able to promote BP reduction, autonomic cardiac modulation, reduction of glycemia, systemic inflammation and modify markers of oxidative stress in diabetic hypertensive patients?

4. HYPOTHESES

H0: A session with AVg is NOT able to promote changes in energy demand or pleasure sensation or HPE or autonomic cardiac modulation in diabetic hypertensive patients.

A AVG training program is NOT able to promote BP reduction at rest or autonomic cardiac modulation or glycemic control or inflammatory modifications of diabetic hypertensives.

H1: A session with AVG is capable of promoting changes in energy demand or pleasure sensation or PEH or autonomic cardiac modulation in diabetic hypertensive patients.

A AVG training program is capable of promoting BP reduction at rest or autonomic cardiac modulation or glycemic control or inflammatory modifications of diabetic hypertensives.

5. LITERATURE REVIEW

5.1 Effectiveness of physical exercise for the treatment of hypertension

Hypertension is a condition in which there is a permanent increase in systolic and diastolic blood pressure values (SBC, SBH, SBN, 2006), with a prevalence of approximately 7.6 million deaths worldwide (WILLIAMS, 2010). The treatment of this disease involves pharmacological and non-pharmacological measures (MORAIS et al., 2011). Among the non-pharmacological methods, physical exercise has been indicated as the most important intervention for the prevention, treatment and control of high BP (MacDonald et al., 2002; MORAIS et al., 2011).

The practice of physical exercise has important action on the control of BP to maintain values considered normal. This action occurs at the end of a physical exercise session, where the pressure values decrease and remain below the pre-exercise values, and this reduction can last for up to 22 hours, being called in the literature as post-exercise hypotension (PEH) (LATERZA, RONDON and NEGRÃO, 2007). Blood pressure reduction can be observed in normotensive individuals, but mainly in hypertensive individuals, as found in the literature review of Casonatto and Polito (2009), which collected 53 articles resulting from the literature confirming PEH in hypertensive and prehypertensive patients and with lower Magnitude in normotensive individuals.

The literature shows that the reduction of systolic (SBP) and diastolic (DBP) pressure in hypertensive patients after a physical exercise session can reach from 18 to 20 mmHg and from 7 to 9 mmHg, respectively, for SBP and DBP (KENNEY et al. SEALS, 1993), although more recent studies have shown lower values, as found in the review study, a reduction of SBP of 3.84 mmHg and DBP of 2.58 mmHg (ASH et al., 2013).

The reduction of BP can be influenced by several factors related to physical exercise, such as initial BP, modality, intensity and duration (MACDONALD, 2002). When it comes to initial BP values, it is clear in the literature that the higher the BP value at rest, the greater the pressure drop after exercise, as observed in the study by Pescatello et al. (1991), who evaluated hypertensive and normotensive individuals after aerobic exercise, where the hypertensive group reduced 6 mmHg of SBP and 9 mmHg of DBP after exercise, whereas the normotensive group increased SBP by 5 mmHg and reduced DBP by 2 MmHg.

As for the type of physical exercise, aerobes are consensually recognized about their effects on BP reduction, being the most used as a non-pharmacological treatment of hypertension (BERMUDES et al., 2003; PESCATELO et al., 2004). The efficacy of aerobic exercise is found in the study by Muniz, Manuchaquian and Andrade (2010) who evaluated 11 middle-aged hypertensive patients who underwent a 30-minute aerobic exercise session and were evaluated for 30 minutes after exercise. The results were a reduction of SBP of 10 mmHg and DBP of 6 mmHg at the end of the recovery period.

Another type of physical exercise is strength that has been studied regarding the reduction of BP over hypertension (CORNELISSEN and FAGARD, 2005). In a study by Melo et al (2006), with medicated hypertensive patients, who underwent bodybuilding exercises, in which the BP was evaluated for up to 21 hours, a mean SBP reduction of 12.0 ± 3.0 mmHg and a DBP of 6,0 ± 2.0 mmHg, respectively, in the first 120 minutes of recovery, and mean BP over 21 h was lower in the exercise group (123.0 ± 4.0 mmHg) than in the control group (128, 0 ± 5.0 mmHg).

In addition to these modalities, there are other less studies that may influence BP control, such as the recreational practice of a football match, which promoted a reduction of 13.8 ± 11.0 mmHg (SBP) and 8.8 ± 5, 0 mmHg (DBP) in middle-aged hypertensive patients (Nóbrega et al., 2013). Another modality that has been well studied is yoga, being approached as effective in the reduction of hypertensive BP (TYAGI and COHEN, 2014). However, among the studies found, the evaluation of this modality is performed in a chronic manner, showing a reduction of SBP of 4.17 mmHg and DBP of 3.26 mmHg (HAGINS et al., 2013). The study by Wolff et al. (2013), where 24-hour BP monitoring was performed after a yoga session, showed a reduction of DBP (4.4 mmHg), showing no changes in SBP.

An activity that has been associated with maintenance of BP is the practice of AVG. However, only two studies were found in the literature, the first study was carried out with normotensive children, bringing a tendency to reduce BP after performing a AVG session (RAUBER et al., 2013), which leads us to think That this reduction may be more evident in the hypertensive population. The second study was performed with healthy youths (21.0 ± 1.6 years) and did not investigate PEH, but compared the BP values obtained immediately after four types of games with rest, resulting in more cardiovascular system demand, varying The SBP of 110 to 140 mmHg and the DBP of 60 to 80 mmHg for the, among the game types (PERRIER-MELLO et al., 2014).

Regarding the intensity and duration of exercise, the most recommended for antihypertensive treatment are the moderate intensity exercises that, according to the VI Brazilian Hypertension Guidelines, are between 60% and 80% of the MHR (SBC, SBH, SBN, 2010), and duration of 30 to 60 minutes (FORJAZ et al., 2004). However, review articles show that intensities as diverse as 40% to 90% of maximal capacity and durations between 10 and 170 minutes, as shown in the review study by Casonatto and Polito (2009), have led to the occurrence of HPE. Another review study found that intensities between 30% and 75% of maximal capacity and duration of 15 to 50 minutes led to reduction of SBP and / or DBP (ANNUNCIATION and POLITO, 2010).

However, the magnitude and duration of BP reduction may vary according to the intensity and duration of the exercise. As for intensity, some studies show that exercises performed at higher intensities lead to a reduction in BP for a longer period of time when compared to low and moderate intensity exercises (PIEPOLI et al., 1994; FORJAZ et al., 2004). Regarding this theme, there is a tendency to perform high-intensity exercise sessions, where Brito et al. (2014) showed that a session of high-intensity muscle strengthening exercises (80% of 1 RM) led to a significant reduction of 33 mmHg (SBP) and 15 mmHg (DBP), while the moderate intensity session (50% of 1RM) reduced 23 mmHg (SBP) and 7 mmHg (DBP).

Regarding the influence of exercise duration, some authors affirm that short-term exercises generate a smaller BP reduction for less time, when compared to the longer exercise (FORJAZ, 1998). However, Guidry et al. (2006), who carried out their study with hypertensive men (43.4 ± 1.5 years), found a reduction in SBP of 5.6 ± 2.0 mmHg and 4.3 ± 1.6 mmHg, respectively, in the (15 minutes) and long duration (30 minutes) and intensity of 40% of maximal oxygen consumption and 4.1 ± 1.6 mmHg and 4.9 ± 1.9 mmHg, in the same sessions, but with intensity of 60 % of maximum oxygen consumption. For DBP the reductions were 2.1 ± 1.0 mmHg (15 minutes) and 3.6 ± 1.4 mmHg (30 minutes) in 60% and 2.4 ± 1.0 mmHg (30 minutes) in 40%. Therefore, the intensity vs. Duration seems to be more determinant in PEH than the isolated action of such variables (JONES et al., 2007).

Considering all these evidences that a physical exercise session is effective in the reduction of BP, when a physical training is performed, the effect of the exercise is in the reduction of the BP of rest, with the continuous accomplishment of the practice of physical exercise, being used as a non-pharmacological treatment of hypertension (ARAÚJO, 2001; HAMER, 2006). It has been demonstrated that physical training causes autonomic and hemodynamic changes such as the reduction of sympathetic nervous activity, peripheral vascular resistance and cardiac output, factors that influence BP reduction (HALLIWILL et al., 1996; REZK et al., 2006.

The literature shows important evidence of the efficacy of physical training of different modalities on BP, describing resting systolic reductions of 3.8 to 11 mmHg, and diastolic reductions of 2.6 to 8 mmHg in hypertensive patients, after aerobic training of at least two weeks (HALBERT et al., 1997; HAGBERG et al., 2000; WHELTON et al., 2002). In a study by LIU et al. (2012), where an eight-week aerobic training was conducted, with weekly frequency of four times and sessions of 30 minutes to 65% of maximal oxygen consumption, a reduction of 7.0 ± 1.4 mmHg (SBP) and 5.2 ± 1.2 mmHg (DBP) in prehypertensive patients.

5.2 Efficacy of physical exercise for the treatment of type II diabetes mellitus

Type II diabetes mellitus (DMII) is one of the most frequent chronic diseases that has had an increase in prevalence worldwide (DORNAS, OLIVEIRA and NAGEM, 2011). In 2013, Brazil was the fourth country in the world in number of cases, with almost 13.4 million cases (SBD, 2013-2014). When glucose ingestion occurs, the action of insulin is to bind to receptors to mediate the entry of glucose into cells. When insulin is not produced or when its receptors become desensitized, leading to hyperinsulinemia and consequent insulin resistance, this action does not occur and glucose accumulates in the bloodstream, generating hyperglycemia. (SBD, 2013-2014).

There is evidence that changes in lifestyle, including reduced levels of physical activity and practice of sedentary behavior are associated with a marked increase in the prevalence of IBD, in addition, this disease is usually accompanied by other chronic pathologies such as hypertension and obesity. Primary prevention and treatment programs for IBD are based on interventions in routine eating and physical activity. (SDB, 2013/2014). Results of the Diabetes Prevention Program show a 58% reduction in the incidence of cases of IBD by stimulating a healthy diet and practicing physical activity, being more effective than the use of drugs (BARCELÓ et al., 2003)

Physical exercise has some important effects that influence insulin resistance, such as reduction of accumulated lipids, performance in inflammatory mediators, and glucose transport and reduction of oxidative stress (BASSUK and MANSON, 2005). Thus, exercise practice stabilizes blood glucose, reduces insulin resistance, and glycated hemoglobin in patients with DMII, (Dela et al., 1995; Boulé et al., 2001; O'Hagan, De Vito and Boreham, 2013). It is known that the reduction of 1% of glycated hemoglobin was associated with a 37% reduction in the risk of microvascular complications and reduced 21% of deaths (STRATTON et al., 2006), so physical exercise is considered the key element for This condition (COLBERG et al., 2010).

The type of physical exercise most recommended by the health guidelines is the aerobic ones that involve large muscle groups, of moderate intensity (50% of the MHR) to vigorous (> 70% of the MHR) and weekly frequency of three to seven days . Muscle strengthening (resisted) muscles should be multi-articular, with two to four sets of eight to ten repetitions, ranging from one to two minute series, in a session containing five to ten exercises directed at the main muscle groups, with an intensity of eight to ten maximum repetitions. The weekly frequency should be two to three times / week non-consecutive. Flexibility exercises should be performed in a complementary way to these modalities (Martber et al., 2009, COLBERG et al., 2010, SBD, 2014).

It is possible to perceive the action of the exercise on DMII markers already after a physical exercise session, with increased glucose consumption that generates a healthy hypoglycemic effect (MERCURI and ARRECHEA, 2001). The results of Van Dijk et al. (30% of the maximum capacity), a reduction in mean blood glucose (-16.2 mg / dL) and a non-occurrence of hyperglycemia in 31% of the cases. The study by Oberlin et al. (2014) who evaluated nine diabetics after a 60-minute session of aerobic exercise at 75% of the MHR, showed a reduction in plasma glucose during 24 hours and a reduction of post-prandial glucose during 48 hours after the session.

Van Dijk et al. (2012) also evaluated 30 diabetics after a strength session (45 minutes) that significantly reduced blood glucose (from 133.2 mg / dL to 122.4 mg / dL) and a prevalence of hyperglycemia of 35% To 33%, when compared to the control group. A combined exercise session (aerobic and strength) resulted in reduction of capillary glycemia (179.03 ml / dL to 148.04 ml / dL) (SILVA and LIMA, 2002). Figueira et al. (2013), brought a mean 24h blood glucose reduction after an aerobic exercise session (from 151.0 ± 8.0 mg / dL to 124.0 ± 9.0 mg / dL) as well as after a combined exercise session (Aerobic and strength) (from 147.0 ± 9.0 mg / dL to 125.0 ± 6.0 mg / dL).

However, for exercise to be considered an effective tool in the treatment of this disease, the results should be added in training sessions (DUCLOS et al., 2011). The literature confirms the efficacy of aerobic training for the management of IBD. Monteiro et al. (2010) evaluated the effect of 13 weeks of aerobic treadmill training with a duration of 50 minutes / session, a three-fold weekly frequency in intensity of 60% to 80% of MHR in diabetic elderly women and showed a reduction in capillary glycemia of 175.3 ± 73.8 mg / dL to 105.3 ± 25.8 mg / dL).

Another study that investigated the practice of aerobic training in diabetics was by Bacchi et al. (-0.40%) and glycemia (-15.2 mg / dL), respectively. The results obtained in the present study were: DL). Similarly, a study by Sigal et al. (2007) evaluated the response to six-month aerobic training, with a frequency of four times a week, in type II diabetic patients aged 39 to 70 years, achieving a reduction in glycated hemoglobin concentrations (-0.38%).

Regarding strength exercises, it is also known of its effectiveness, but as a type of exercise complementary to aerobes (SBD, 2013/2014). However, there are studies in the literature that show its effects on variables related to diabetes, without being accompanied by aerobic exercises. Although the literature shows that 12-week interventions are effective in improving glycemic control, studies show that in eight weeks of resistance training, a reduction in glycated hemoglobin (O'HAGAN, DE VITO and BOHERAM, 2013).

Baldi et al. (2003) evaluated nine diabetics during the ten-week resistance training, three sessions / week, with ten exercises per session, and progressive intensity from 10 RM to 15 RM, and found a non-significant reduction of 8.9 ± 0, 8% for 8.4 ± 0.6% of glycated hemoglobin. However, longer studies also show interesting results, as can be seen in the study by Castaneda et al. (2002), where 31 participants underwent resistance training for 16 weeks, three sessions per week, five exercises per session between 60% and 80% of 1RM, which also reduced glycated hemoglobin (from 8.7 ± 0.3% to 7,6 ± 0.2%).

Although the results shown in the above studies have relevance, it is worth mentioning that these exercises may be more effective if performed in a combined way (PRAET et al., 2006). In a more recent study, Sigal et al. (2007) compared three types of training (aerobic, resisted and combined) for six months, resulting in a reduction of glycated hemoglobin from 7.46% to 6.99% at three months and 6.56% at six months of combined training. The aerobic training resulted in a reduction from 7.41% to 7.00% at three months and 6.98% at six months and resistance training from 7.48% to 7.35% at three months and 7.18% % At six months of training.

In addition to these more studied modalities, there are the less studied modalities of exercise that can be effective as a treatment tool for IBD, such as yoga, where a review study shows changes in blood glucose after a month of pranayma practice, a yoga branch that (From 148.19 ± 43.13 mg / dL to 108.19 ± 21.05 mg / dL), in addition to other studies showing reduction of glycated hemoglobin after three months of yoga practice (-1.96% ) And improvement of insulin receptors (3.74 ng to 5.81 ng) (SAHAY, 2007). A study by Leelayuwat (2013) suggests the importance of practicing alternative modalities in the treatment of IBD, such as tai-chi-chuam, and dance.

As for these modalities, Hung et al. (2009), who evaluated 28 diabetics for 12 weeks (3 sessions / week) practicing tai-chi-chuam, obtained fasting blood glucose reduction (-18.0 mg / dL) after the intervention. While Manjeri et al. (2014) evaluated 47 diabetic subjects for six months of a dance class program (individual and salon), showing a reduction of glycated hemoglobin (-0.36%) at the end of the study.

Other important aspects about the performance of physical exercise as a form of treatment of IBD are the intensity used and the duration of exercise, where the literature shows different intensities addressed. A study by Jeng et al. (2002) evaluated the glycemic response after exercise sessions with different intensities (40%, 60% and 80% of maximal effort) and different durations (10, 20, 30 and 40 minutes), resulting in a significant intensity vs. Duration (F = 11,756). Silveira et al. (2014) evaluated the acute effect (10, 20 and 30 minutes after exercise) at 60% intensity and 80% of 1RM, resulting in similar blood glucose reduction.

A meta-analysis by Boulé et al. (2003) who evaluated training programs of nine studies with a mean duration of 20 weeks, 3.4 weekly sessions with distinct intensities (50% and 75% of maximum oxygen consumption), stated that aerobic exercises of higher intensities generate additional benefits To DMII patients such as cardiorespiratory improvement and glycated hemoglobin concentrations. About this importance, Fex et al. (2014) evaluated high-intensity interval training in diabetics and noted a significant reduction in blood glucose after 12 weeks. In addition, the study by Gillen et al. (2012) who evaluated seven diabetics for 24 h after a high-intensity interval exercise session (60 seconds at 89% of maximum oxygen consumption and 60 seconds of recovery), which led to the reduction of postprandial glycemia when compared to control.

Having as parameters all the information about the influence of physical exercise in the DMII, physical exercise is assumed to be increasingly a fundamental part of the treatment and control of this disease, however it must be practiced regularly to have the continued benefits.

5.3 The evolution of video game and its function as a therapeutic treatment

Technological advancement and the practicality of modern life provide contemporary man with a sedentary lifestyle (FLORINTO, GUIMARÃES and CESAR, 2009). Sedentary behavior, also called sitting time (TREMBLAY et al., 2011) has been associated with the onset of chronic-degenerative diseases (Queiroz et al., 2013). In the present study, a group of patients with a history of dyslipidemia and hypertension (Tavares et al., 2010;

On the other hand, the practice of physical activity has been recommended to prevent and treat these diseases (SIGAL et al., 2006; ALVEZ et al., 2007), however, only 30.3% of Brazilian adults report reaching Physical activities recommended in leisure activities (MINISTÉRIO DA SAÚDE, 2012). Technology preoccupied with the increase of diseases related to sedentary behavior and physical inactivity has provided great evolution in the world of video game games (UNNITHAN, 2006).

Recently active games have emerged, which may also be called active exergames, interactive games and video games and have been investigated by researchers who assess their potential for health promotion (PENG et al., 2012). Active video games (AVG's) are conceptualized as games in which the practitioner replaces the traditional joystick with a sensor that leads him to perform the movements that he wants performed by the avatar on the screen, where participants are required to move body segments or The entire body in front of the camera to control the game (STAIANO and CALVERT, 2011; PEREIRA et al., 2012).

There are several types of VGA, such as Dance Dance Revolution (SSD Company Ltd., Shiga, Japan) which uses an electronic dance mat with a selection of songs (LANNINGHAM-FOSTER et al., 2006), EyeToy Kinetic (Sony Computer EntertainmentEurope (SSD Company Ltd., Shiga, Japan), which provides sporting activities such as golf, tennis, boxing and boxing. Bowling and includes a game rug (XaviX J-Mat) allowing for walking or running in the virtual world (LANNINGHAM-FOSTER, 2006). In addition to these, there is also the Nintendo Wii ™ (Consolidated financial high lights) system, a device composed of a motion sensor and Wii Remote ™ that resembles a wireless remote control and allows the performance of sports activities (SERIOUS, STRATTON and RIDGERS, 2007).

Other examples of AVG are Your Shape Fitness, Just Dance, Zumba, among others, which use the Xbox Kinect ™ console (Foxconn®, New Taipei, China), the most advanced technology, Of the body of animated virtual characters (BAO et al., 2013), because it captures movements to project the image of the player on a screen located in a virtual game environment (O'DONOVAN et al., 2012; HOLMES et al., 2013) , As well as being preferred by users, probably due to the amount of movement provided by their sensors (ACSM, 2013; PERRIER-MELO, 2013).

In the study by Maddison et al. (2011), which evaluated the practice of these games for 24 weeks on body composition in overweight and obese adolescents, and found reductions in body mass index (BMI) and body fat. However, the study by Madsen et al. (2007), who evaluated 30 overweight children and adolescents between the ages of 9 and 18, during the same practice period with Dance Dance Revolution did not find a reduction in BMI.

In the case of cognitive control, Kevin et al. (2011) investigated the efficacy of a 20-minute session with Wii Fit on congnitive control in young adults (18 to 25 years), finding that AVG's are not effective in cognitive control through increased attention resource and greater control of Interference during cognitively demanding activities, as you can see in a treadmill exercise session. Regarding balance, it is possible to find a review article by Perrier-Melo et al. (2013) on balance in the elderly, where when evaluating eight studies that used the AVG as a physical therapy tool, concluded that the AVG's are able to improve the balance in this population.

About cardiac rehabilitation, in a study by Broeren et al. (2008), where 11 patients with a mean age of 68 years after stroke had a four-week intervention, three sessions / week with AVG, achieving an improvement in motor performance. Like this one, Garcia et al. (2010) who evaluated adults and elderly, also obtained motor performance improvement after physical therapy intervention with AVG.

Only one study investigated the influence of Dance Dance Revolution (30-minute session) on BP in children with a mean age of 9.8 years, where there was a significant increase of SBP and mean BP during the game compared to the session (SBP - 116.9 ± 6.9 mmHg vs. 102.8 ± 7.2 mmHg; mean BP - 89.6 ± 4.6 mmHg vs. 81.1 ± 4.9 mmHg) and one (103.5 ± 4.7 mmHg at rest and 99.5 ± 3.7 mmHg at 40 minutes post AVG) (RAUBER et al., 2013).

According to a study that evaluated the influence of AVG on BP, it used the XboX kinect console and the Dance Central and Kinect Sports games (boxing, volleyball, tennis and dance) played 10 minutes each. Eight young adults were evaluated, and it was found that the four types of AVG altered the cardiovascular system, with BP varying from 110 to 140 mmHg (systolic) and from 60 to 80 mmHg (diastolic) (PERRIER-MELLO et al., 2014).

However, when studying a large number of studies using the various AVG’s, it can be seen that most of the studies used use the populations of children, adolescents and young adults. Few studies address the elderly population, and no study was conducted with the middle-aged adult population, especially when it comes to people suffering from chronic diseases such as hypertension and diabetes.

6. METHODOLOGICAL PROCEDURES

6.1 Type of research: According to the objectives outlined, this study can be characterized as being of the almost experimental type, according to Gaya, 2008.

6.2 Study Volunteers and Sample Calculus: 34 women will participate in this study. The inclusion criteria will be: 1) age between 45 and 59 years; 2) present BMI between 25 and 33 kg / m²; 3) be hypertensive (present a minimum initial systolic blood pressure of 130 mmHg and diastolic blood pressure of 90 mmHg; 4) present a diagnosis of diabetes; 5) not to practice regular physical exercise; 6) to be menopause (not having menstruation for the minimum period of a given year); 7) have no prior experience with AVG of any kind; 8) not be affected by labyrinthitis. And as exclusion criteria: 1) initiate the use of beta-blockers and calcium channel blockers during the study; 2) present epileptic episodes; 4) not be willing to perform all the sessions of the acute experimental protocol in a period of two months; 5) missing two consecutive sessions and / or more than 5 sessions (about 20%) of the total number of sessions of the chronic experimental protocol.

To determine the sample size, a sample calculation was performed using the Gpower 3.0 software. Data from a pilot study with a sample drawn from the same population were taken as the basis for this calculation. A 5% alpha error and a 95% beta error were used, using SBP and DBP with effect size of 1.01 and 0.57, respectively, thus reaching a sample size of 34 participants who will participate in the study. As to the sample size of the chronic intervention of the study, it was not possible to perform the same sample calculation, since an adequate study was not found in the literature. Thus, the data from the first six participants will be collected, and the sample calculation will be performed from these data.

The volunteers will be recruited at near Campus I of the Federal University of Paraíba, from the authorized entry of the researcher, by the Health Department of the Municipality of João Pessoa, once approved by the Research Ethics Committee, the researcher You will enter a process for applying for authorization for this recruitment. This project will be submitted to the Ethics Committee and after all procedures have been clarified, participants will be asked to sign the Free and Informed Consent Form (TCLE) in accordance with National Health Council resolution 466/12 (appendix A).

6.3 Study design: volunteers will initially be evaluated for clinical and outpatient PA, cardiac autonomic modulation (CAM) and biochemical variables through blood collection, in addition, will perform an ergospirometric test. In an interval between 48h and 5 days after the initial visit, they will perform protocols for active and sedentary video game games, two treadmill exercise / treadmill exercise protocols and one control procedure. These sessions will be performed on separate days with a minimum interval of 48h and the order of accomplishment will be randomized, adopting the crossover model, where all participants will perform all the sessions. In these procedures, they will be monitored for energy expenditure / displacements, feeling of pleasure and effort. BP measurements, heart rate (HR), CAM will be performed before and / or during and / or during a recovery period following the procedures. At the end of this phase, participants who demonstrate an interest will be randomly divided into AVG group and control for an eight week intervention. In each session will be measured energy expenditure / displacements. Clinical and CAM measurements will be performed every week and blood collections every four weeks. At the end, they will perform a new ergospirometric test and a new ambulatory blood pressure measure.

6.4 Preparation of the subjects: When accepting to participate in the study, an anamnesis will be applied to the acquisition of personal information and drugs used and anthropometric information (stature, body mass and waist circumference) will be collected for characterization of subjects (appendix B). The International Physical Activity Questionnaire (IPAQ) was used to measure participants' level of physical activity (Appendix C). In addition, a nutritional survey will be conducted and participants will be given a list containing caffeine-rich foods and medications (Appendix D) and will be instructed not to ingest the components of this list for 24 hours and alcohol for at least 48 hours before Acute intervention protocol, and prior to MAC and MAPA measurements during the chronic intervention protocol.

6.4.1 International Physical Activity Questionnaire (IPAQ): the International Physical Activity Questionnaire / IPAQ-short version (MATSUDO et al., 2001) is used to determine the level of physical activity of the participants, considered to be valid and Reproducibility similar to other instruments used internationally to measure the level of physical activity (BENEDETTI et al., 2007). Following the recommendations of the World Health Organization (WHO, 1995), women who will be recruited should be classified as physically inactive (those who accumulate less than 150 minutes of mild, moderate or intense activities per week).

6.4.2 Nutritional survey: food consumption was assessed through the 24-hour Reminder (appendix E) and the Food Consumption Frequency Questionnaire (Annex A) applied at the beginning and during the study.

The 24-hour reminder consists of defining and quantifying all food and beverages ingested in the period prior to the interview, which may be 24 hours earlier or, more commonly, the previous day (GIBSON, 1990). This will be applied three times with each individual, being two representative of the food referring to days of the week, and an indicative of the food consumption of the weekend. The analyzes will be done using the average of the three values to investigate the consumption of macro and micronutrients, with emphasis on the presence of antioxidant substances of the diet. For food adequacy analysis, the values obtained will be compared with those recommended by the Dietary Reference Intakes (DRI's) (2002) (OTTEN; HELLWIG; MEYERS, 2006). This instrument will be applied and evaluated by a nutritionist, using Avanutri Revolution software version 4.0 (Avanutri Informática Ltda, Rio de Janeiro, Brazil).

The Food Consumption Frequency Questionnaire is considered to be the most practical and informative dietary intake assessment method, since it allows obtaining retrospective data for longer periods (FISBERG et al., 2005). It will be applied to evaluate the habitual consumption of food sources of antioxidant substances in the months before the start of the intervention protocols and during the study. This questionnaire will be applied and evaluated by a nutritionist using the software Avanutri version 4.0 (AVANUTRI- RJ, Brazil).

6.5 Description of video games used

6.5.1 Description of the active video game: the console will be the XBOX 360 with Kinect sensor (Foxconn®, New Taipei, China) and the game used will be Just Dance Summer Party (Ubisoft®, Montreiul, France). Initially, the Kinect sensor captures the movement signals of all body segments of the player. From there, during the games played, it will be necessary for the player to perform body movements considered active to manipulate the avatar generated on the screen. The level of difficulty of the game adopted will be the beginner, since the participants will not have previous experience with AVG. In the case of the game Just Dance Summer Party, body movements are similar to dance steps, with the use of different songs, as shown in Annex B.

6.5.2 Description of the sedentary video game: the session will be similar to the VGA sessions, with the same duration duration and the same measures of HR, SEP, accelerometry, BP and MAC. In this session Dance Dance Revolution® (SSD Company Ltd., Shiga, Japan) will be used for Playstation 2® (San Mateo, CA, USA), where the practitioner will simulate a synchronized dance, however using the traditionally known joystick and participants will remain Throughout the session in a sitting position. The style of the songs used in the game is similar to those used in gyms.

6.6 Adaptive Protocols

6.6.1 Adaptation to the game: Participants will perform a three-session adaptation period, where the first session lasts 10 minutes and the last one, a 40-minute period that will be reached gradually. During the sessions, participants will be guided in the correct way to handle and use the game.

6.6.2 Adaptation to exercise on a treadmill: on the same days that the sessions of adaptation to the game will occur, the participants will perform treadmill sessions, being the first day, lasting 30 minutes, on the second day lasting 40 minutes And the third day lasting 60 minutes. The intensity adopted in these sessions will be free, however the participants will be encouraged to reach the moderate intensity (60% to 85% of the FCM).

6.7 Procedures for acute sessions: at each session, upon arrival at the collection site, the participants will remain in rest for a period of 20 minutes, then the resting measures of HR, BP and MAC will be performed, after which they will be instrumented With the accelerometer, the portable device for oxygen consumption to measure the energy expenditure / displacements. The chosen session will be started in a randomized fashion with a duration of 60 minutes. HR, oxygen consumption and accelerometry will be measured throughout the session and SEP will be questioned every 10 minutes. Immediately at the end of the session, the BP and MAC will be measured again and repeated for 60 minutes, every 10 minutes, with participants remaining at rest. During this period the Enjoyment Scale will be applied. During the EIM session, where the participants are expected to achieve moderate exercise intensity, the heart rate percentage of 60 and 85% of the MHF (moderate intensity) will be prescribed and this will be performed from the data obtained in the ergospirometric test. In addition, in the EIG, where the adopted heart rate will be similar to that reached in the AVG session, the intensity adopted will not be predetermined. In the AVG and SVG sessions, no pre-established intensities will be adopted.

6.8 Training protocol: Participants will be randomized into two groups (AVG and control). The training will last 8 weeks, with a frequency of 3x / week, totaling 24 sessions. The weekly sessions will take place on intercalary days, considering at least 48h intervals between them and on Sundays there will be no training sessions. The sessions will take place at home, with the presence of the researcher for its realization. Initially the participants will remain at rest for 10 minutes, then the BP and resting HR will be measured. The training session will begin with the AVG, where energy expenditure / displacements, HR and SEP will be measured during the 40-minute game. Immediately at the end of the game session, the BP will be re-measured and the Enjoyment Scale will be applied. The control group will not perform any activity and will have weekly visits for BP and MAC measurement.

6.9 Description of the variables measured

6.9.1 Heart Rate Measurements and Subjective Effort Perception: subjects will be instrumented with the Polar brand heart rate monitor, model RS800cx (Polar ElectroOy, Kempele, Finland) and at the beginning of each session, will be seated for 10 minutes to be Measured the resting HR. This variable will be monitored throughout the session.

During the sessions of VGA adaptation and ergometer exercise, Borg's Subjective Effort Perception Scale (SEP) (NOBLE et al., 1983) will be presented to the participants with indices of 6 to 20 so that they become familiar with the Stages of fatigue ranging from very mild to exhaustive. The subjective perception of effort will be measured every 10 minutes during the sessions.

6.9.2 Protocol for blood pressure measurements at the collection site: participants will be asked to remain at rest for 10 minutes and after this period the baseline BP will be checked. In the acute intervention protocol sessions, new measures will be taken at rest, immediately at the end of the active video game session, and every 10 minutes during a 60-minute recovery period. During the chronic intervention protocol, these measures will be performed at the participants' residence. These measurements will be performed by the auscultatory method following the V Brazilian Guidelines for Hypertension (2010).

6.9.3 Ambulatory Blood Pressure Monitoring Protocol (ABPM): Ambulatory Blood Pressure Monitoring (ABPM) will be performed the week of the initial study visit, as well as at the end of the chronic intervention protocol. BP and HR will be monitored for a 24-hour period using a Cardios® model Dyna-MAPA + device (São Paulo, Brazil). The monitor will be programmed to perform the measurements every 15 minutes during the wake-up period and every 30 minutes during sleep, so that at the end of the 24 hours, at least 16 measures valid during the And seven during sleep, according to the recommendations of the V Brazilian Guidelines for Ambulatory Blood Pressure Monitoring (MAPA V) and III Brazilian Guidelines for Residential Blood Pressure Monitoring (MRPA III) (2011).

6.9.4 Ambulatory Blood Pressure Monitoring Protocol (ABPM): Ambulatory Blood Pressure Monitoring (ABPM) will be performed the week of the initial study visit, as well as at the end of the chronic intervention protocol. BP and HR will be monitored for a 24-hour period using a Cardios® model Dyna-MAPA + device (São Paulo, Brazil). The monitor will be programmed to perform the measurements every 15 minutes during the wake-up period and every 30 minutes during sleep, so that at the end of the 24 hours, at least 16 measures valid during the And seven during sleep, according to the recommendations of the V Brazilian Guidelines for Ambulatory Blood Pressure Monitoring (MAPA V) and III Brazilian Guidelines for Residential Blood Pressure Monitoring (MRPA III) (2011).

The installation protocol of the device will be in accordance with the Brazilian MAPA V and MRPA III Guidelines (2011). Initially, the circumference of the arm will be measured to select the cuff of adequate width and length. A clinical measure of BP will be previously performed with the volunteer sitting after 10 minutes of rest in both upper limbs using a mercury column sphygmomanometer before installing the device. The cuff will be placed 2 to 3 cm above the cubital fossa in the non-dominant arm. After the placement of the equipment, the measurement obtained by the MAPA monitor will be compared with the measurement obtained previously with the mercury column sphygmomanometer, making sure that the differences do not exceed 5 mmHg. Each participant will be given an activity diary to record the activities performed during the 24 hours, as well as containing all instructions for the examination (Annex C).

6.9.5 Ergospirometric test protocol: the assessment of maximal aerobic capacity will be performed through the direct measurement of oxygen consumption at peak exercise (peak VO2). The protocol used will be the ramp, where the incline of the ramp will be increased every two minutes of the test duration. Simultaneously with the stress test, the individual will be connected to a computerized ergospirometer (Vmax, USA®) through a valve and sensor system where pulmonary ventilation (LV) will be measured with each expiration. O2 and CO2 (CO2) sensors will analyze the concentrations of O2 and CO2, respectively, for each respiratory cycle. From the analyzes of the LV and the concentrations of expired gases, VO2 and CO2 production will be calculated. It will be considered as peak VO2 the consumption of O2 obtained at the peak of the exercise, when the individual can no longer sustain the intensity of the race on the treadmill. In addition to determining the individual's maximum functional capacity, the anaerobic threshold (LA) and the respiratory decompensation point (PDR) will be determined (SKINNER, 1980). LA will be considered the minute that the individual has lower O2 (VE / VO2) and O2 partial pressure at the end of the expiration (PetO2) before they have a progressive increase and a nonlinear increment Of the respiratory exchange rate (RER) value. The respiratory compensation point will be considered the minute that the individual presents lower values of CO2 ventilatory equivalent (VE / VCO2) before there is a progressive increase of this and the maximum value of CO2 partial pressure at end of expiration (PetCO2) Before beginning to show a progressive decline.

6.9.6 Cardiac Autonomic Modulation (CAM) recording: it will be evaluated by means of heart rate variability, a simple and non-invasive measure of the autonomic impulses originating in the bulb and directed to the heart, indicating a measure of the activity of the nervous system Autonomous in cardiovascular regulation. This measure will be done by means of evaluation of the oscillations in the interval between consecutive heart beats (R-R intervals) of the heart rate (VANDERLEI et al., 2009).

The CAM will be determined by recording the variability of the heart rate R-R intervals through a Polar heart rate monitor, model RS800CX (PolarElectroOy, Kempele, Finland). This instrument was validated before recording with electrocardiogram at rest and during exercise (NUNAN et al., 2008; PORTO; JUNQUEIRA, 2009). The subjects will be in rest for 10 minutes, after which the recording of the R-R intervals will begin. This record will be made, with the volunteers seated, for a minimum period of five minutes to obtain a record of at least 300 beats. The data will be transferred to a computer equipped with software from the same manufacturer and then transferred to Kubios HRV software, version 2.0 (University of Kuopio, Finland). The data will be analyzed in the time domain, considering the mean and standard deviation of the individual R-R intervals. In the frequency domain, bands of low frequency (0.04 to 0.15 Hz) and high frequency (0.15 to 0.4 Hz) will be considered as measures of electrical activity from sympathetic and parasympathetic impulses, respectively. The low frequency / high frequency ratio will also be adopted as an autonomic balance.

6.9.7 Accelerometer: Actigraph® GT3SX (Pensacola, USA) Accelerometer for Body Acceleration Assessment, calibrated according to manufacturer's specifications, will be used during all sessions. The time interval adopted will be in minutes and the data output expressed in average counts per minute. The device will be fixed at waist height, on the right side, by an elastic strap and adjustable buckle or in the participants' clothing. At the end of the records, the data will be transferred to a computer and will be analyzed using SAS 9.2 software (SAS Institute Inc., Cary, NC 25513). The reference values adopted will be: 0 to 99 counts / min-1, it will be considered a sedentary activity, 100 to 1951 counts / min-1, moderate activity and from 1952 to 5723 counts / min-1, it will be considered vigorous activity.

6.9.8 Oxygen consumption through the portable pulmonary gas analyzer K4b2: this portable equipment enables the verification of respiratory parameters at each breath by measuring oxygen consumption and carbon dioxide production during physical exercise. The instrument used will be the portable lung gas analyzer K4 b2 (Cosmed Copyritgh ©, Rome, Italy) validated in the study by McLaughlin et al. (2001) and will be used during the acute intervention sessions.

6.9.8 Enjoyment Scale: This scale is composed of 18 items that will evaluate the level of pleasure / enjoyment that the activity performed can provide, where each item has seven levels, level one corresponds to "I I like it a lot "and level seven corresponds to" I hate "(appendix F). This scale was validated for use in the adult population according to Graves et al. (2010) and its score is calculated through the average values obtained in each item and the higher the average, the more pleasure / enjoyment the activity performed provided.

6.9.9 Blood collection protocol: During the initial visit to the study as well as in the fourth week of chronic intervention and at the end of the study, an experienced nurse will collect 10 mL of venous blood taken from the antecubital vein of each volunteer after a fast of 12 Hours. Samples will be centrifuged at 3000 rpm for 15 minutes and the supernatant (serum or plasma) transferred into microtubes and refrigerated at -20øC or 4øC until analysis.

6.10 Biochemical Dosages

6.10.1 Glycemic and lipid profile: Analyzes of the glycemic and lipid profile will be performed on serum samples using commercial kits from the Labtest brand (Minas Gerais, Brazil), following the recommendations of the manufacturer and on a Labmax 240 premium automatic analyzer (Lagoa Santa -MG, Brazil). Blood glucose concentrations will be determined by the enzymatic colorimetric method of glucose oxidase proposed by Trinder (1969). The absorbance will be obtained at wavelength 505nm.

Total cholesterol will be determined by the enzymatic method proposed by Trinder (1969) at 500nm. HDL-c will be quantified by manual method. For this procedure, a volume of 0.25 μL of precipitating substance will be added to 0.25 μL of sample in microtubes and vigorously mixed for 30 seconds. It was then centrifuged at 3,500 rpm for 15 minutes, The supernatant being withdrawn and placed into aliquots containing 1 μl of reagent 1 from the Cholesterol Liquiform kit, and placed in the water bath for 10 minutes. Finally, the ultraviolet spectrophotometer (Biospectro, model SP-220 / Brazil) will be read at 500 nm.

The triglyceride values will be determined by the enzymatic method proposed by Trinder (1969), and the absorbance will be obtained at wavelength 505nm. The values of low density lipoproteins (LDL-c) and very low density lipoproteins (VLDL-c) will be estimated by the Friedewald equation (LDL-C = (CT-HDL-C) - (TG / 5)) (FRIEDEWALD, LEVY and FREDRICKSON, 1972).

The reference values for the lipid and glycemic profile variables of adults are shown in table 1.

|  | Low | desirable | Limít | High | Very High |
| --- | --- | --- | --- | --- | --- |
| **Glucose**  **(mg/dL)** |  | 70-99 |  |  |  |
| **TC**  **(mg/dL)** |  | < 200 | 200-239 | ≥ 240 |  |
| **HDL-c**  **(mg/dL)** | < 40 (Men)  < 50 (Women) | ≥ 60 | 40-59 (Men)  50-59 (Women) |  |  |
| **LDL-c**  **(mg/dL)** |  | < 100 | 130-159 | 160-189 | ≥190 |
| **TG**  **(mg/dL)** |  | < 150 | 150-199 | 200-499 | ≥500 |

Source: American Association of Clinical Endocrinologists (2012)

6.10.2 Analysis of glycated hemoglobin (HbA1): its concentration will be quantified in plasma samples that can be stored for up to 8 days. The quantification will be determined by the commercial kit of the Labtest brand (Minas Gerais, Brazil) according to the manufacturer's instructions through the microchromatography method. Values between 4% and 6% are within the normal range of HbA1 concentration. Values and around 7% is the appropriate value for people affected by Diabetes. Values above 8% are in the range of risk for chronic complications.

6.10.3 Analysis of ultra-sensitive c-reactive protein (hs-CRP): the concentration of hs-CRP will be quantified by immunoturbidimetry in serum samples. Concentrations of hs-CRP will be determined using the commercial kit of the Labtest brand (Minas Gerais, Brazil) as instructed by the manufacturer. Calibration will use the Calibra Calibrator from Labtest (Calibra Plus PCR-ultra - Ref-345). The absorbance will be obtained in the Labmax 240 premium automatic analyzer (Lagoa Santa-MG, Brazil), at wavelength 540nm. Serum reference values for hs-CRP will be based on cut-off points for risk of cardiovascular events in the adult population: <1.0 mg / L (low risk), 1.0-3.0 mg / L (medium risk) and > 3.0 mg / L (high risk) according to Pearson et al. (2003).

6.10.4 Analysis of Alfa-1-acid glycoprotein (A1GPA): The concentration of A1GPA will be quantified by immunoturbidimetry in serum samples using the commercial kit (Labtest, Minas Gerais, Brazil) as instructed by the manufacturer. For calibration, the Calibra Calibrator from Labtest (Calibra Plus Protein - Ref-346) will be used. The absorbance will be obtained in the Labmax 240 premium automatic analyzer (Lagoa Santa-MG, Brazil), at wavelength 340nm.

6.10.5 Antioxidant activity by DPPH (Radical 2,2-diphenyl-1-picryl-hydrazyl): the quantification of serum antioxidant levels will be performed according to the methodology described by Chrzczanowicz et al. (2008). It will be carried out by means of a spectrophotometric test using a solution of 2,2-diphenyl-1-picryl-hydrazila (DPPH) in methanol (MeOH), mixed to the solution of the sample under analysis in different concentrations. After 30 minutes of reaction, the absorbances of the solutions will be determined at 540 nm and in a spectrophotometer (Bioespectro, model SP 22, Brazil). The DPPH radical molecule exhibits maximum absorption at 540 nm and violet coloration that turns yellow when reduced. This reduced form corresponds to the DPPH free radical molecule paired with an antioxidant hydrogen (DPPH-H). The resulting discoloration is stoichiometric, with the number of radical molecules sequestered (MOLYNEUX, 2004).

Deproteinization of the serum will be optimized by testing with 9.5 M acetonitrile (CH 3 CN) in H 2 O, 100% CH 3 CN and methanol (CH 3 OH) 100%. At the end, 200mL of CH 3 CN will be added in 200mL of serum and the mixture incubated for two minutes at room temperature and centrifuged for ten minutes at 11,000rpm at 4 ° C, 25mL of the supernatant, which corresponds to the deproteinized serum, is withdrawn. To this fraction will be added 970 mL of CH3OH and 5 mL of DPPH solution. The mixture will be vortexed, allowed to stand at room temperature for 20 minutes, centrifuged for ten minutes and then subjected to 11,000 rpm at 4 ° C. 200 mL of the supernatant from each sample transferred to a 96-well microplate, reading at 540 nm. The reference solution (blank) will consist of 25μL H2O instead of the blood serum volume (sample).

6.10.6 Analysis of antioxidant activity (SOD): the antioxidant activity will be determined by the activity of the enzyme superoxide dismutase (SOD), according to Sun et al. (1988). This will be assessed by its ability to inhibit the photochemical reduction of nitro-tetrazolium blue (NBT). The results will be calculated as the amount of SOD required to inhibit the NBT reduction rate by 50%. Samples will be centrifuged for 10 minutes at 3600 rpm at 4 ° C. The supernatant will be withdrawn and centrifuged again for 20 min at 12,000 rpm at 4 ° C. In a dark chamber, 1mL of the reaction medium (50mM phosphate buffer, 100nM EDTA and 13mM L-methionine pH 7.8) will be mixed with 30 μL of the sample, 150μL of 75μM NBT and 300μL 2μM riboflavin. The tubes containing the obtained solution will be exposed to fluorescent lamps (15W) for 15 minutes. At the end, the material will be read in a spectrophotometer at a wavelength of 560nm.

6.10.7 Analysis of Malondialdehyde (MDA): Oxidizing activity will be quantified by the reaction of thiobarbituric acid (TBARS) with the products of decomposition of hydroperoxides. For this, 250 μl of sample will be incubated in a water bath at 37 ° C for 60 minutes. The sample will then be precipitated with 35% AA perchloric acid and centrifuged at 14,000 rpm for 20 minutes at 4 ° C. The supernatant will be transferred to new microtubes where 400μl of 0.6% thiobarbituric acid will be added and incubated at 60 ° C for 60 minutes. After cooling, the material will be read in a Biospectro (SP-220 / Brazil) spectrophotometer at a wavelength of 532nm.

6.10.8 Nitric Oxide (NO) analysis by quantification of plasma nitrite: The endogenous NO production will be determined as a function of the plasma concentration of its nitrite metabolite. The concentration of nitrite was determined by the Griess reaction that quantifies the nitrite in the sample through the diazotizing reaction forming a pink color chromophore. The reagent was prepared using equal parts of 5% phosphoric acid, 1% sulfanilamide in 5% phosphoric acid, 0.1% N-1-Naphtyl) -ethylenediaminedihydrochloride (NEED) and distilled water. Nitrite / nitrate detection is followed by the addition of 500 μL Griess reagent to 500 μL of the plasma. After 10 minutes, the absorbance will be measured in a spectrophotometer (Biospectro, SP-220 / Brazil) at a wavelength of 532nm. The nitrite concentrations will be calculated by extrapolation to a standard NaNO2 curve and the data expressed in micromoles (GREEN, TANNERNBAUM and GOLDMAN, 1981).

All the biochemical analyzes will be performed in the Laboratory of Studies in Physical Training Applied to Performance to Health (Department of Physical Education - Federal University of Paraína), the biochemical variables being the glycemic and lipid profile and systemic inflammation performed in the LABMAX 240 Premiun automatic analyzer (Labteste Diasgnóstica S / A, Minas Gerais, Brazil) biochemical variables related to oxidative stress will be performed in the same place, however using the manual method of analysis.

6.11 Statistical analysis: the data will be applied Shapiro-Wilk tests to verify normality and Levene to verify homogeneity. If the data is distributed in a normal way, the results will be presented as mean and standard error of the mean. The statistical test used to compare resting values, as well as comparisons between measurements during the different sessions of the acute intervention protocol, will be ANOVA one way. For comparisons between the measurements during the recovery period of the acute intervention protocol sessions, as well as for comparisons between the moments before, during and after the sessions of the chronic intervention protocol, two way ANOVA will be used. For comparisons between the two different chronic intervention groups, once the pre - and post - post moments were compared, the independent Student 's t test will be used and for pre - post comparison of each of the chronic intervention protocol groups, Student's t-test was used. The data will be analyzed using Instat 3.0 software (GraphPad, San Diego, CA, USA), adopting significance of p <0.05.





Tradution

CERTIFICATE

Certificate of qualification for research at the Health Sciences Center of the Federal University of Paraíba - CEP / CCS unanimously approved at the 1st Meeting held on 02/19/2015, the research project entitled "Efficacy of active video game in Blood pressure and Glycemia in diabetic hypertensives: acute and chronic effects ", by the researcher Taís Feitosa da Silva, Protocol 0621/14. CAAE: 39010114.2.0000.5188.

Furthermore, the authorization for subsequent publication is subject to the submission of the summary of the proposed study to the committee's consideration.
